# Characterization of Cancer Stem Cell Characteristics and Development of a Prognostic Stemness Index Cell-Related Signature in Oral Squamous Cell Carcinoma

**DOI:** 10.1155/2021/1571421

**Published:** 2021-11-09

**Authors:** Jin Feng, Yunxia Li, Ning Wen

**Affiliations:** ^1^Department of Stomatology, Chinese PLA General Hospital, Beijing 100853, China; ^2^Department of Outpatient, Beijing Corps Hospital of People's Armed Police, Beijing 100027, China

## Abstract

**Objective:**

Cancer stem cells (CSCs) with self-renewal and plasticity contribute to tumor initiation and progression. This study developed an mRNA expression-based stemness index- (mRNAsi-) associated signature and validated biological functions of stem cell-related genes in oral squamous cell carcinoma (OSCC).

**Methods:**

Here, mRNAsi was measured for OSCC samples from TCGA cohort, and prognosis and tumor microenvironment (stromal/immune scores, tumor purity) in high- and low-mRNAsi samples were evaluated with survival analyses and ESTIMATE algorithm. Based on prognostic mRNAsi-related genes, a risk score model was constructed by the LASSO method. The predictive accuracy was evaluated by uni- and multivariate Cox analyses and ROC curves. Among the genes in the model, the functions of H2AFZ on proliferation, apoptosis, invasion, and EMT were investigated in OSCC cells.

**Results:**

High mRNAsi was distinctly associated with undesirable prognosis, increased stromal and immune scores, and lowered tumor purity. The mRNAsi-associated signature containing 11 genes was developed, and high-risk score was distinctly related to poor survival outcomes. Moreover, this signature was an independent and robust risk factor. H2AFZ upregulation significantly enhanced proliferative and invasive capacities and facilitated EMT as well as lowered apoptotic levels in Cal-27 and HSC-3 cells.

**Conclusion:**

Our study characterized cancer stem cell characteristics that were closely related to tumor microenvironment and developed a stemness index cell-related signature that could assist prognosis prediction and risk stratification for OSCC. H2AFZ could become a potential therapeutic target against OSCC.

## 1. Introduction

Oral carcinoma represents a common malignancy among humans, with over 90% of cases deriving from oral squamous cell carcinoma (OSCC) [1]. As a multifactorial malignancy, common risk factors contain tobacco, drinking, and human papilloma viruses [2]. Over 300,000 new cases of OSCC occur each year globally, and over 140,000 patients die of OSCC each year [3]. The five-year survival rate is merely 50% [4]. Meanwhile, most metastatic patients die within 12 months [5]. The conventional treatment for OSCC is a combination of curative resection, radiotherapy, and chemotherapy [6]. Nevertheless, in the past 30 years, there has been no distinct progress in the treatment of OSCC. Uncovering the molecular mechanisms underpinning OSCC is important, which may accelerate the development of personalized therapeutic strategies.

Recent advancements have suggested that cancer stem cells (CSCs) that are featured by self-renewal and plasticity exert a key function on OSCC development and growth [7]. Furthermore, CSCs contribute to radio- and chemoresistance as well as are in relation to undesirable survival outcomes and cancer recurrence for OSCC [8]. Stemness characteristics have been measured by new stemness index, such as DNA methylation- and mRNA expression-based stemness index (mRNAsi) [9]. This mDNAsi has been reported as a prognostic indicator in few cancers, thereby helping predict cancer progression and guide clinical therapy. Previously, Zhang et al. proposed an mRNAsi-related signature that might independently predict prognosis and stratify risk for lower-grade glioma [10]. As described by Bai et al., overexpression of mRNAsi was detected in liver cancer as well as was related to tumor pathological grade and poor survival duration [11]. Nevertheless, no study has attempted to explore the characteristics of CSCs in OSCC on the basis of mRNAsi. Herein, we characterized the interactions of mRNAsi with prognosis as well as tumor microenvironment in OSCC. Also, an mRNAsi-related signature was developed as an accurate and independent prognostic factor of OSCC. Furthermore, we investigated the functions of mRNAsi-related gene H2AFZ in OSCC cells. Our data demonstrated that H2AFZ upregulation significantly enhanced proliferative and invasive capacities and facilitated EMT as well as suppressed apoptotic levels for Cal-27 as well as HSC-3 cells, demonstrating that H2AFZ might be a promising therapeutic target against OSCC.

## 2. Materials and Methods

### 2.1. Patients and Datasets

Transcriptome profiles by RNA sequencing (RNA-seq; FPKM values) of 328 OSCC tissues were obtained from The Cancer Genome Atlas (TCGA; https://tcga-data.nci.nih.gov/tcga/) on March 11, 2020. FPKM values were transformed into TPM values. Meanwhile, the matched clinical characteristics including age, gender, grade, stage, T, M, and N as well as survival information were also retrieved from TCGA database (Supplementary Table 1). By the Ensemble database (http://asia.ensembl.org/index.html), the Ensemble IDs were converted into gene symbols.

### 2.2. Acquisition of mRNAsi

As described by Malta et al., mRNAsi of each OSCC sample was calculated using a one-class logistic regression machine learning algorithm (OCLR) [9]. mRNAsi was expressed utilizing *β* values that were ranged from 0 (without mRNA expressions) to 1 (complete mRNA expressions). mRNAsi was retrieved through the multiple platform analyses according to the previous study [9]. Patients were stratified into high- and low-mRNAsi groups on the basis of median mRNAsi. The prognostic value of mRNAsi was determined, and overall survival (OS) between subgroups was analyzed via Kaplan-Meier curves as well as log-rank tests using “survival” and “surviminer” packages.

### 2.3. Estimation of Immune Score, Stromal Score, and Tumor Purity

By employing Estimation of STromal and Immune cells in MAlignant Tumours using Expression data (ESTIMATE) algorithm, the fractions of stromal cells as well as immune cells were inferred in OSCC tissues using gene expression signatures. Based on stromal and immune scores, tumor purity was then estimated. Comparisons of immune/stromal scores and tumor purity in high- and low-mRNAsi groups were achieved with Wilcoxon tests.

### 2.4. Inferring Tumor-Infiltrating Immune Cells

The CIBERSORT method (http://cibersort.stanford.edu/) was utilized to quantify 22 tumor-infiltrating immune cell fractions in each OSCC tissue based on gene expression profiling [12]. The immune cells included naive B cell, memory B cell, plasma cell, CD8+ T cell, naive CD4+ T cell, CD4+ resting memory T cell, CD4+ memory activated T cell, T cell follicular helper, T cell regulator, T cell gamma delta, resting/activated natural killer cell, monocyte, M0/M1/M2 macrophage, resting/activated dendritic cell, resting mast cell, activated mast cell, eosinophil, and neutrophil. Significant results (*p* < 0.05) were screened for further analyses.

### 2.5. Analysis of Differentially Expressed Genes (DEGs)

With “limma” algorithm, DEGs were screened between high and low-mRNAsi samples [13]. Genes with ∣fold change | >1.5 as well as false discovery rate (FDR) < 0.05 were abnormally expressed. These DEGs were visualized into volcano plots.

### 2.6. Functional Annotation Analyses

Using “clusterProfiler” package, functional annotation analyses of mRNAsi-related DEGs were presented for investigating and visualizing biological functions and pathways involving above genes, including Gene Ontology (GO) function annotations as well as Kyoto Encyclopedia of Genes and Genomes (KEGG) [14]. Terms with FDR < 0.05 were considered significantly enriched.

### 2.7. Development of a Prognostic Model

Through univariate Cox regression analysis, DEGs that were closely related to OSCC survival were screened. These prognosis-related key DEGs were optimized through least absolute shrinkage and selection operator (LASSO) analyses with “glmnet” package [15]. The variable selection and the shrinkage of prognosis-related candidate DEGs were then carried out. Afterwards, a risk score model was generated by multivariate Cox regression analysis based on the coefficient and expression of each gene. According to the median value, OSCC patients were clustered into two subgroups. Survival status was observed in two groups. Moreover, OS analyses in patients with high and low risk were achieved by Kaplan-Meier curves. Differences in OS were estimated with log-rank tests. The expression of the mRNAsi-associated genes was visualized into a heat map via pheatmap package. Differences in clinicopathological features (age, sex, grade, and stage) were estimated in high- and low-risk subgroups. The predictive accuracy of the signature was assessed via the area under the curve (AUC) of receiver operator characteristic curve (ROC). Uni- and multivariate Cox analyses were achieved for estimating the predictive independency of this signature. The expression of the mRNAsi-associated genes was detected in 519 OSCC and 44 normal tissues from TCGA dataset. Kaplan-Meier survival curves were conducted for evaluating the prognostic significance of the mRNAsi-associated genes among OSCC patients in TCGA dataset.

### 2.8. Gene Set Enrichment Analysis (GSEA)

The potential molecular mechanisms underlying the mRNAsi-associated signature were carried out via the GSEA method [16]. Enriched KEGG pathways were explored for high- and low-risk samples. Pathways with FDR < 0.05 were significantly enriched.

### 2.9. Development of a Prognostic Nomogram and Assessment of Its Predictive Performance

Factors that were independently predictive of survival were incorporated to construct a prognosis-associated nomogram for investigating the probability of 1-, 3-, and 5-year OS in OSCC patients with glmnet package [15]. The predictive accuracy of the nomogram was estimated by ROC curves. The calibration curves were plotted for observing the nomogram-predicted and observed survival probabilities. Meanwhile, decision curve analyses (DCA) were presented for calculating the clinical net benefit of each factor in comparison to all or none factors [17]. The best model was the one with the highest net benefit as calculated.

### 2.10. Patients and Specimens

Three pairs of fresh OSCC and adjacent normal tissues were collected from OSCC patients who received surgery in the Department of Stomatology, Chinese PLA General Hospital. None of the patients experienced chemoradiotherapy prior to surgery. This study protocol gained the approval of the Ethics Committee of Chinese PLA General Hospital, with written informed consent acquired from each patient (2020-039).

### 2.11. Cell Culture and Transfection

OSCC cells Cal-27 and HSC-3 (Shanghai Cell Bank of Chinese Academy of Sciences (China)) were maintained in DMEM (Gibco, USA) containing 10% fetal bovine serum (FBS). These cells were cultivated in a constant temperature box containing 5% CO_2_ and 37°C. They were cultivated onto 6-well plates (5 × 10^5^ cells/well). SiRNA targeting H2AFZ (si-H2AFZ; 5′-CACCGAGACGCTCGATGACTCCGC-5′ (sense), 5′-AAACGCGGAGTCATCGAGCGTCTC-3′ (antisense); RiboBio, China) and its negative control (si-NC) as well as pcDNA3.1-H2AFZ (RiboBio, China) and empty vector were transfected into cells via Lipofectamine™ 2000 transfection kit (Life Technologies, USA). After 48 h, H2AFZ expression was measured by real-time quantitative polymerase-chain reaction (RT-qPCR) and western blot.

### 2.12. RT-qPCR

Total RNA was extracted from transfected Cal-27 and HSC-3 cells according to the Trizol kit (TIANGEN, USA). The concentration and purity of total RNA were measured. Through GoScript reverse transcription system kit (Promega, USA), total RNA was used to obtain cDNA by reverse transcription reaction. The primer sequences were as follows: H2AFZ: 5′-GGCGGTAAGGCTGGAAAGG-3′ (forward), 5′-TGTCGATGAATACGGCCCAC-3′ (reverse); GAPDH: 5′-GGAGCGAGATCCCTCCAAAAT-3′ (forward), 5′-GGCTGTTGTCATACTTCTCATGG-3′ (reverse). The qPCR reaction was then carried out. The PCR reaction conditions contained predenaturation at 95°C lasting 10 min, denaturation at 95°C lasting 15 s, annealing at 60°C lasting 20 s, and extension at 72°C lasting 20 s, a total of 40 cycles. Afterwards, H2AFZ as well as GAPDH expressions were automatically read and generated in the PCR instrument. The relative expressions of H2AFZ were determined utilizing 2^-*ΔΔ*Ct^ methods.

### 2.13. Cell Counting Kit-8 (CCK-8)

Cal-27 and HSC-3 cells were seeded onto 96-well plates (5000 cells/well). At the indicated time points (0, 24, 48, and 72 h), 10 *μ*L CCK-8 reagent (Dojindo, Japan) was added to cells and incubated for 1 h. The absorbance at 450 nm was detected for reflecting cell viability using a spectrophotometer.

### 2.14. Flow Cytometry Assay

Cal-27 and HSC-3 cells following transfection were seeded onto 6-well plates (1 × 10^5^ cells/well). After 24 h, the cells were trypsinized without ethylenediaminetetraacetic acid. Then, cells were centrifuged and were treated with 5 *μ*L Annexin V-FITC (Beyotime, China) as well as 10 *μ*L PI at room temperature in the dark for 15 min. BD FACSCalibur Flow cytometry was applied to detect cell apoptosis. Finally, results were evaluated with CellQuest software (BD Biosciences).

### 2.15. Transwell Assay

Cell invasion was measured with 8 *μ*m Transwells. The membrane was coated with Matrigel. Cal-27 as well as HSC-3 cells following transfection were seeded onto the upper chambers (1 × 10^5^ cells/well). DMEM plus FBS was added into the lower chambers. Following 24 h, the cells from the upper surface of the membrane were scraped and removed using cotton swab. The invaded cells were stained with 0.4% crystal violet. Five fields were counted under an inverted microscope (Olympus, Japan).

### 2.16. Western Blots

Total proteins were extracted from OSCC and normal tissues as well as Cal-27 and HSC-3 cells. Then, BCA kit (Beyotime, China) was utilized for measuring the extracted protein. Protein was then separated with 10% sodium dodecyl sulfate-polyacrylamide gel electrophoresis gels and transferred to PVDF membrane. After being blocked using 5% nonfat milk, membranes were incubated by primary antibodies targeting H2AFZ (1 : 1000; ab214730, Abcam, USA), N-cadherin (1 : 1000; ab98952, Abcam, USA), Vimentin (1 : 1000; ab137321, Abcam, USA), and GAPDH (1 : 1000; ab8245, Abcam, USA) at 4°C overnight, followed by secondary antibodies. The protein bands were developed using enhanced chemiluminescence kit and were quantified by ImageJ software.

### 2.17. Statistical Analysis

R version 3.6.1 (https://www.r-project.org/) and GraphPad Prism software version 8.0.1 were applied for statistical analysis. The R packages were retrieved from Bioconductor (http://www.bioconductor.org/). Data are displayed as mean ± standard deviation. Wilcoxon test, Student's *t*-test, and one-way analysis of variance were employed for comparing differences between subgroups. *p* < 0.05 was indicative of statistical significance.

## 3. Results

### 3.1. Characterization of Correlation between mRNAsi and Prognosis and Tumor Microenvironment of OSCC

**Figure 1:**
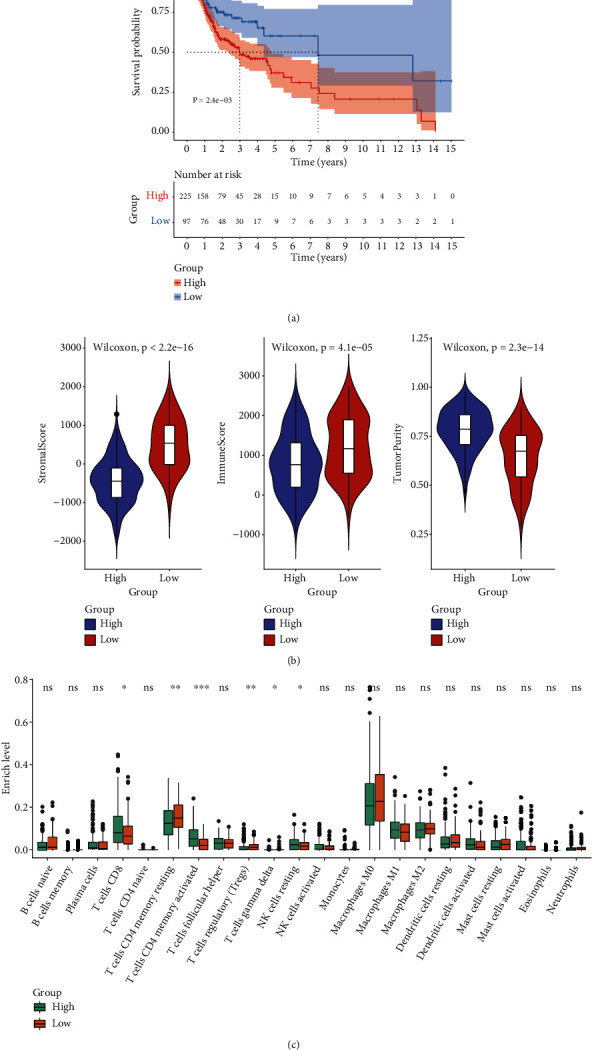
Prognostic value of mRNAsi and its correlation with immune microenvironment in OSCC. (a) Survival analyses of OSCC patients with high and low mRNAsi with Kaplan-Meier curves. *p* values were estimated through log-rank tests. (b) Differences in stromal/immune scores and tumor purity in high- and low-mRNAsi groups. (c) Comparisons of the enrichment levels of immune cells in two groups with the CIBERSORT method. *p* values were determined with Wilcoxon tests. ns: not significant; ^∗^*p* < 0.05; ^∗∗^*p* < 0.01; ^∗∗∗^*p* < 0.001.

This study quantified CSCs by mRNAsi scores for OSCC patients from TCGA cohort (Supplementary Table 2). According to the median value, these patients were clustered into high- and low-mRNAsi groups. Survival analyses were carried out for investigating the survival differences in subjects with high- and low-mRNAsi scores. In Figure 1(a), more undesirable survival outcomes were found in the high-mRNAsi group compared to the low-mRNAsi group. Through the ESTIMATE method, we evaluated stromal/immune scores as well as tumor purity in OSCC specimens. There were increased stromal score and immune score as well as lowered tumor purity in the high-mRNAsi group than the low-mRNAsi group (Figure 1(b)). Tumor-infiltrating immune cells were inferred utilizing the CIBERSORT method. Increased infiltration levels of CD8+ T cell, activated memory CD4+ T cell, and resting NK cell were found in high-mRNAsi specimens (Figure 1(c)). Meanwhile, there were higher infiltration levels of resting memory CD4+ T cell in low-mRNAsi specimens.

### 3.2. Analysis of mRNAsi-Associated Genes and Their Biological Implications

**Figure 2:**
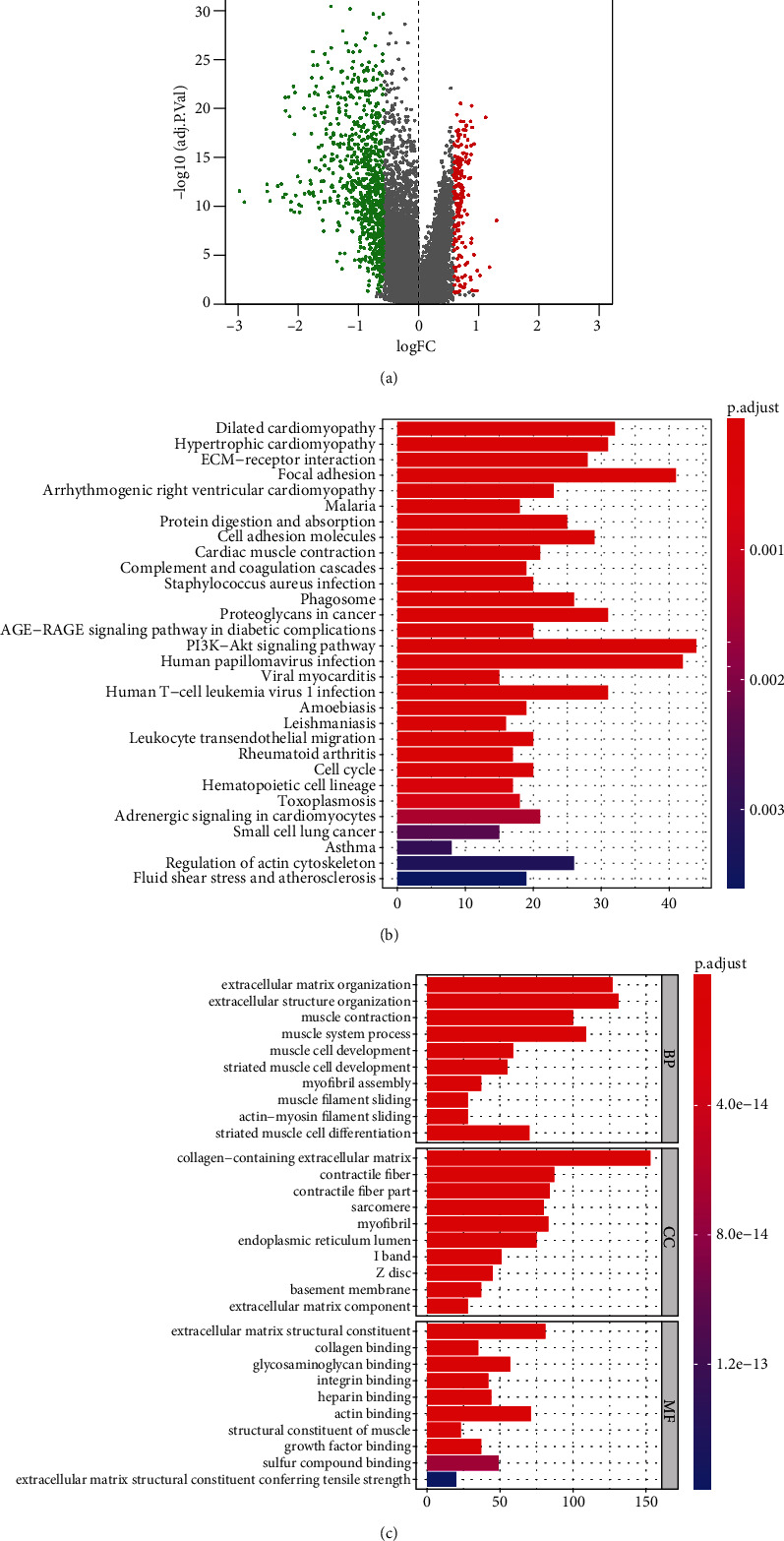
Identification of mRNAsi-related DEGs and their biological implications. (a) Volcano plots of 197 upregulated and 533 downregulated genes in high-mRNAsi samples than low-mRNAsi samples. Red dots represented upregulated genes, and green dots represented downregulated genes. (b) KEGG pathways involving the mRNAsi-related DEGs. The more the color tended to red, the smaller the adjusted *p* value. The longer the bar, the greater the number of enriched DEGs. (c) GO annotation terms of the mRNAsi-associated DEGs.

To identify mRNAsi-associated genes of OSCC, genes with ∣fold change | >1.5 and FDR < 0.05 were screened (Supplementary Table 3). In Figure 2(a), 833 mRNAs displayed upregulation and 197 mRNAs displayed downregulation in high-mRNAsi specimens than low-mRNAsi specimens. Furthermore, these mRNAsi-associated genes were distinctly enriched in tumor-related pathways such as ECM-receptor interaction, proteoglycans in cancer, and PI3K-Akt pathway (Figure 2(b)). GO annotation results demonstrated that these genes were distinctly associated with extracellular matrix organization (Figure 2(c)).

### 3.3. Generation of a Prognostic mRNAsi-Associated Signature for OSCC

**Figure 3:**
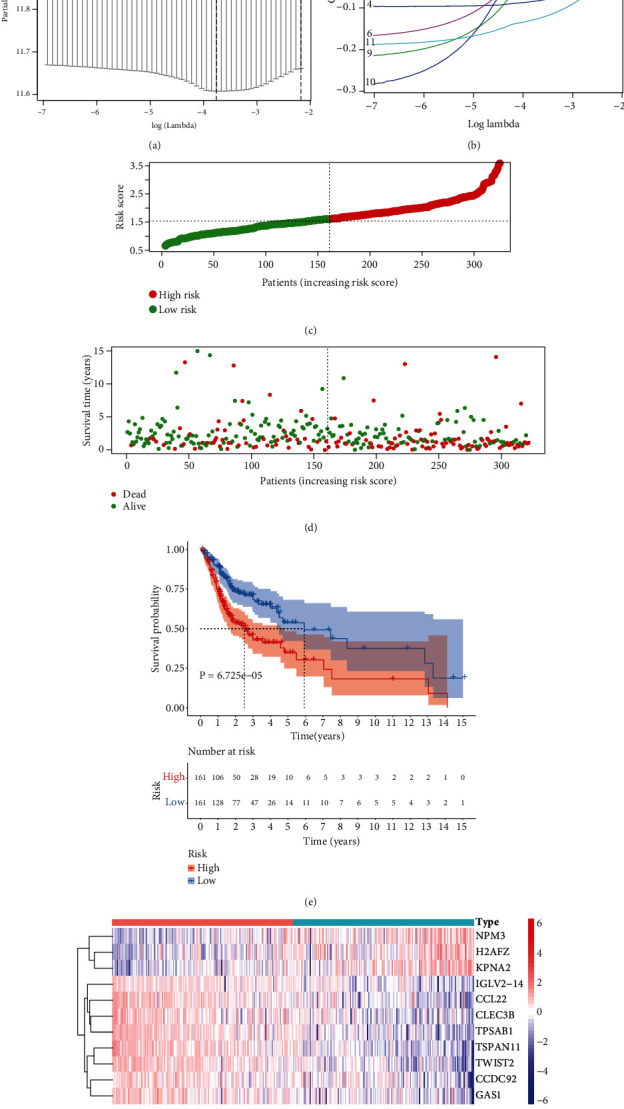
Establishment of a prognosis-associated mRNAsi-related signature for OSCC. (a) Tenfold cross-verification for tuning parameter selection in the LASSO regression model. Two dotted vertical lines were plotted for the optimal values through the minimum criteria. (b) LASSO coefficient profiles of selected mRNAsi-related signatures. (c) Visualization of risk scores of OSCC patients. The median mRNAsi score was utilized as the cutoff value (dotted vertical line). All patients were clustered into high- and low-risk score groups. Red dots: high-risk specimens and green dots: low-risk specimens. (d) Visualization of dead and alive OSCC patients with high- and low-risk score. Red dots represented dead status, and green dots represented alive status. (e) Survival analyses of OSCC subjects with high- and low-risk scores with Kaplan-Meier curves. (f) Heat map of the expression of the mRNAsi-related signatures in specimens with high- and low-risk scores. Red represented upregulation and purple represented downregulation.

Through univariate Cox regression analyses, 14 mRNAsi-associated genes were distinctly correlated to prognosis of OSCC patients (Table 1). With the LASSO method, 11 optimal candidate biomarkers were selected for constructing a prognosis risk score model (Figures 3(a) and 3(b)). The risk score of each OSCC patient was determined, as follows: H2AFZ expression∗0.0150584034611393 + KPNA2 expression∗0.171144144263817 + CCDC92 expression∗(−0.08377988547353) + GAS1 expression∗(−0.0247182891302941) + NPM3 expression∗0.0519125455291411 + CCL22 expression∗(−0.0581447729360218) + TSPAN11 expression∗(−0.0616269466917819) + CLEC3B expression∗(−0.033743441760667) + TWIST2 expression∗(−0.127555711866237) + TPSAB1 expression∗(−0.0345953977371839) + IGLV2 − 14 expression∗(−0.0208527855597802). All patients were clustered into two groups based on the median mRNAsi (Figure 3(c)). More death cases were found in the high-mRNAsi group than the low-mRNAsi group (Figure 3(d)). In Figure 3(e), high-mRNAsi patients displayed more unfavorable survival than low-mRNAsi patients. NPM3, H2AFZ, and KPNA2 were upregulated in low-mRNAsi samples while CCDC92, GAS1, CCL22, TSPAN11, CLEC3B, TWIST2, TPSAB1, and IGLV2-14 were upregulated in low-mRNAsi samples (Figure 3(f)).

### 3.4. Assessment of the Predictive Accuracy of the mRNAsi-Associated Signature for OSCC Prognosis

**Figure 4:**
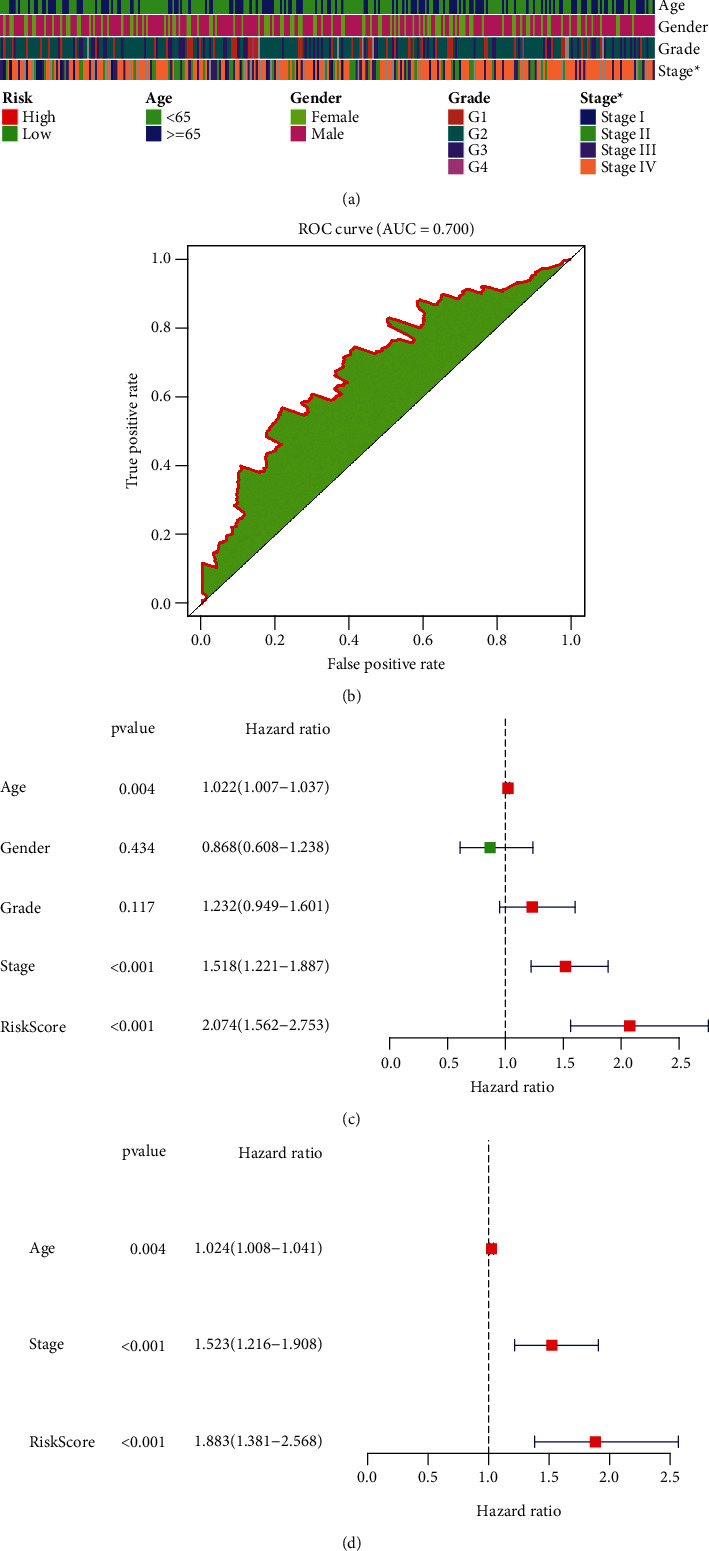
Estimation of the relevant clinicopathological characteristics and predictive capacities of the mRNAsi-related gene signature in OSCC. (a) Heat map of the correlation of risk scores to clinicopathological features: age, gender, grade, and stage. ^∗^*p* < 0.05. (b) Estimation of the predictive capacity of the model in survival of OSCC patients. (c) Univariate Cox regression analyses of age, sex, grade, stage, and risk score for OSCC prognosis. (d) Multivariate Cox analyses for age, stage, and risk scores for OSCC prognosis.

The associations between the mRNAsi-associated signature and clinicopathological characteristics were evaluated in OSCC patients. In Figure 4(a), this signature was markedly associated with stage of OSCC. The AUC of the signature was 0.700, demonstrating the well-predictive accuracy of this signature in predicting survival (Figure 4(b)). As shown in uni- as well as multivariate Cox analyses, age, stage, and risk scores might be independently predictive of OSCC prognoses (Figures 4(c) and 4(d)).

### 3.5. Signaling Pathways Involving the mRNAsi-Associated Signature

**Figure 5:**
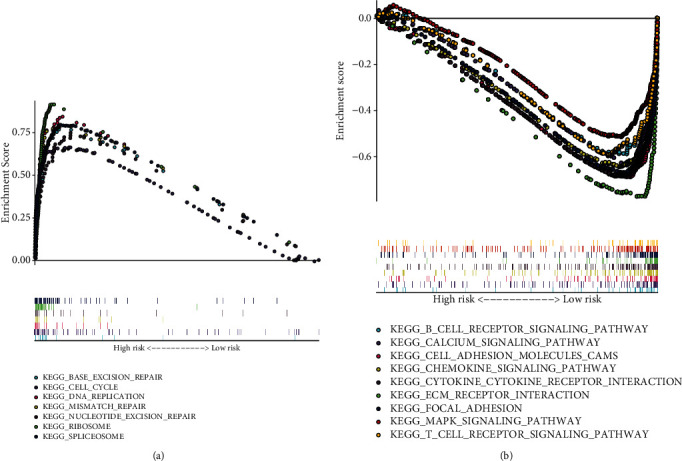
Signaling pathways involving the mRNAsi-related gene signature by GSEA. (a) Activated pathways in high-risk score OSCC samples: base excision repair, cell cycle, DNA replication, mismatch repair, nucleotide excision repair, ribosome, and spliceosome. (b) Activated pathways in low-risk score OSCC samples: B cell receptor, calcium, cell adhesion molecules cam, chemokine-chemokine receptor interaction, ECM receptor interaction, focal adhesion, MAPK, and T cell receptor pathways.

GSEA was carried out to attempt to explain the intrinsic mechanisms underlying the mRNAsi-associated signature. In Figure 5(a), base excision repair, cell cycle, DNA replication, mismatch repair, nucleotide excision repair, ribosome, and spliceosome were distinctly activated in high-risk OSCC. Meanwhile, B cell receptor, calcium, cell adhesion molecules cam, chemokine-chemokine receptor interaction, ECM receptor interaction, focal adhesion, MAPK, and T cell receptor pathways were markedly activated in low-risk samples (Figure 5(b)).

### 3.6. Generation of a Prognostic Nomogram for OSCC

**Figure 6:**
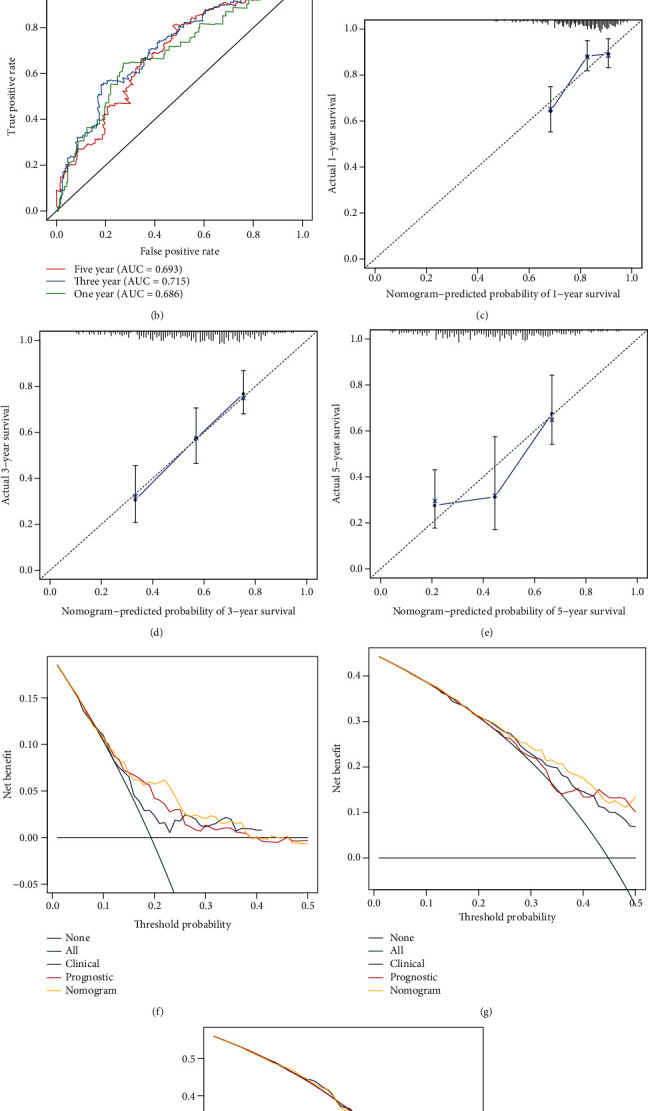
Generation of a prognostic nomogram for OSCC. (a) Establishment of a nomogram by incorporating age, stage, and risk score for predicting 1-, 3-, and 5-year survival. (b) Estimation of the predictive ability of the nomogram in OSCC prognosis using the ROC curves. (c–e) Calibration plots for showing the deviation between model-estimated and observed 1-, 3-, and 5-year survival. (f–h) Decision tree analyses for the nomogram in predicting 1-, 3-, and 5-year OS duration in OSCC patients.

Independent risk factors (age, stage, and risk score) were incorporated to generate a nomogram for prediction of OSCC patients' 1-, 3-, and 5-year OS probabilities (Figure 6(a)). The AUCs at 1-, 3-, and 5-year OS were 0.686, 0.715, and 0.693, confirming the well-predictive accuracy of the nomogram in predicting survival (Figure 6(b)). As demonstrated by calibration curves, nomogram-predicted 1-, 3-, and 5-year OS were highly similar to investigated survival (Figures 6(c)–6(e)). Moreover, DCA results confirmed that higher net benefit of the nomogram for 1-, 3-, and 5-year OS was investigated compared to other clinical factors (Figures 6(f)–6(h)). Hence, this nomogram exhibited the well-predictive efficacy in OSCC prognosis.

### 3.7. Validation of the Expression of the mRNAsi-Associated Genes in OSCC

**Figure 7:**
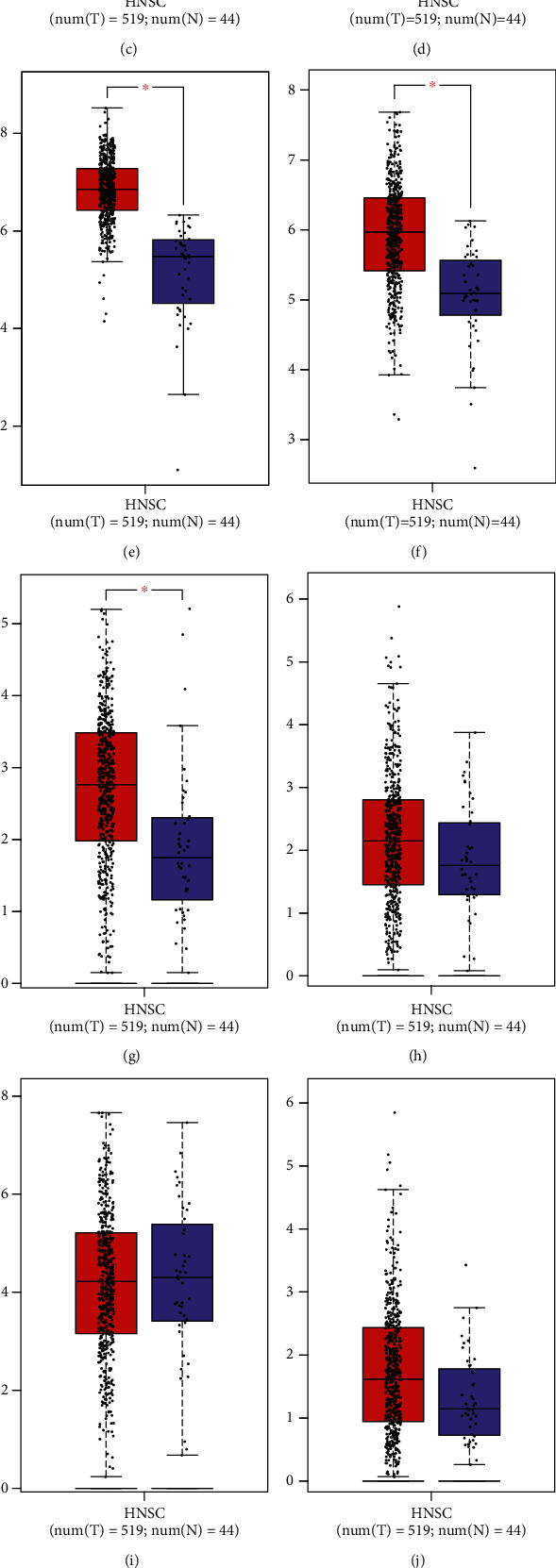
Visualization of the expression of the 11 genes in OSCC and normal tissue specimens: (a) CCDC92, (b) GAS1, (c) H2AFZ, (d) IGLV2, (e) KPNA2, (f) NPM3, (g) TWIST2, (h) CCL22, (i) TPSAB1, (j) TSPAN11, and (k) CLEC3B. (l, m) Western blot for validating the expression of H2AFZ in three paired OSCC and normal tissues. ^∗^*p* < 0.05; ^∗∗^*p* < 0.01.

**Figure 8:**
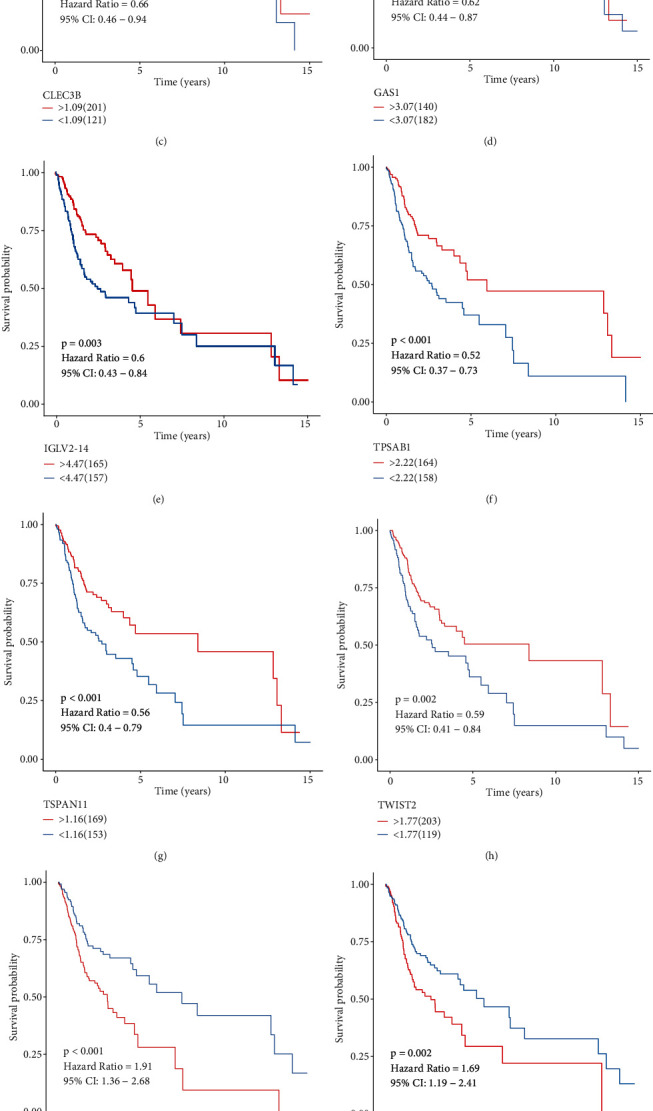
Prognostic significance of the 11 genes in the mRNAsi-related gene signature in OSCC. Kaplan-Meier survival curves were conducted between OSCC patients with high and low expression of (a) CCDC92, (b) CCL22, (c) CLEC3B, (d) GAS1, (e) IGLV2, (f) TPSAB1, (g) TSPAN11, (h) TWIST2, (i) H2AFZ, (j) KPNA2, and (k) NPM3 in TCGA dataset.

The expression of the 11 mRNAsi-associated genes was observed in OSCC and normal tissues. CCDC92, GAS1, H2AFZ, IGLV2, KPNA2, NPM3, and TWIST2 were markedly upregulated in OSCC compared to normal tissues (Figures 7(a)–7(g)). No significant differences in CCL22, TPSAB1, and TSPAN11 were investigated between OSCC and normal tissues (Figures 7(h)–7(j)). Also, CLEC3B displayed decreased expression in OSCC than normal tissues (Figure 7(k)). Among them, H2AFZ expression has been confirmed to be upregulated in several cancer types such as breast cancer [18] and hepatocellular carcinoma [19]. This study proposed the upregulation of H2AFZ in OSCC for the first time. The expression of H2AFZ was further validated in three paired OSCC and normal tissues through western blot. As expected, high expression of H2AFZ was found in OSCC compared with normal tissues (Figures 7(l) and 7(m)). Prognostic significance of the 11 mRNAsi-associated genes was further analyzed across OSCC patients. Our results demonstrated that patients with high expression of CCDC92, CCL22, CLEC3B, GAS1, IGLV2, TPSAB1, TSPAN11, and TWIST2 exhibited the prominent survival advantage (Figures 8(a)–8(h)). In contrast, high expression of H2AFZ, KPNA2, and NPM3 indicated poorer prognosis compared with their low expression (Figures 8(i)–8(k)).

### 3.8. H2AFZ Upregulation Facilitates Proliferation and Lowers Apoptosis in OSCC Cells

**Figure 9:**
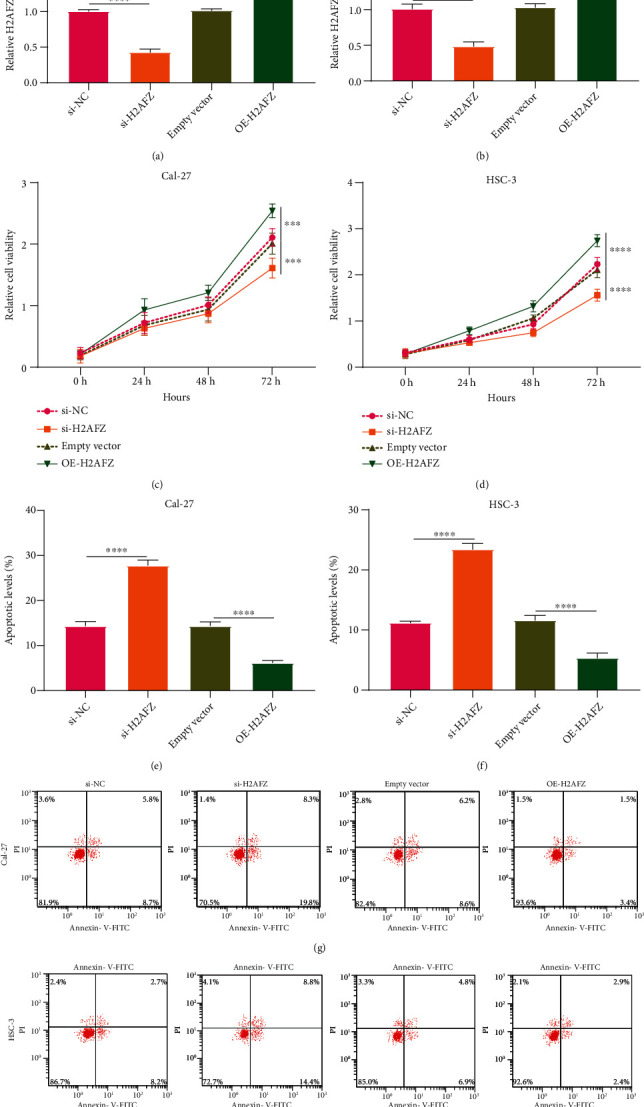
H2AFZ upregulation facilitates proliferation and lowers apoptosis of OSCC cells. (a, b) The mRNA expressions of H2AFZ in Cal-27 as well as HSC-3 cells under transfection with si-H2AFZ and H2AFZ overexpression via RT-qPCR. (c, d) Cell viability of Cal-27 and HSC-3 cells transfected with si-H2AFZ and H2AFZ overexpression by CCK-8. (e–h) Flow cytometry for the apoptotic levels of Cal-27 and HSC-3 cells after transfection with si-H2AFZ and H2AFZ overexpression. ^∗∗∗^*p* < 0.001; ^∗∗∗∗^*p* < 0.0001.

The biological implications of H2AFZ were observed in two OSCC cells. H2AFZ expression was distinctly suppressed by si-H2AFZ as well as was markedly elevated by pcDNA3.1-H2AFZ in Cal-27 as well as HSC-3 cells (Figures 9(a) and 9(b)). CCK-8 assay was carried out for investigating the cell viability of Cal-27 and HSC-3 cells after transfection. Our data demonstrated that si-H2AFZ markedly suppressed cell viability compared to si-NC for Cal-27 as well as HSC-3 cells (Figures 9(c) and 9(d)). Meanwhile, cell viability of Cal-27 as well as HSC-3 cells was significantly heightened under transfection with pcDNA3.1-H2AFZ than empty vector. By flow cytometry, apoptotic levels of Cal-27 as well as HSC-3 cells were observed. As a result, increased apoptotic levels were found in OSCC cells following transfection with si-H2AFZ in comparison to si-NC (Figures 9(e)–9(h)). Also, pcDNA3.1-H2AFZ transfection markedly lowered apoptotic levels of Cal-27 as well as HSC-3 cells than empty vector.

### 3.9. H2AFZ Upregulation Promotes Invasion and EMT in OSCC Cells

**Figure 10:**
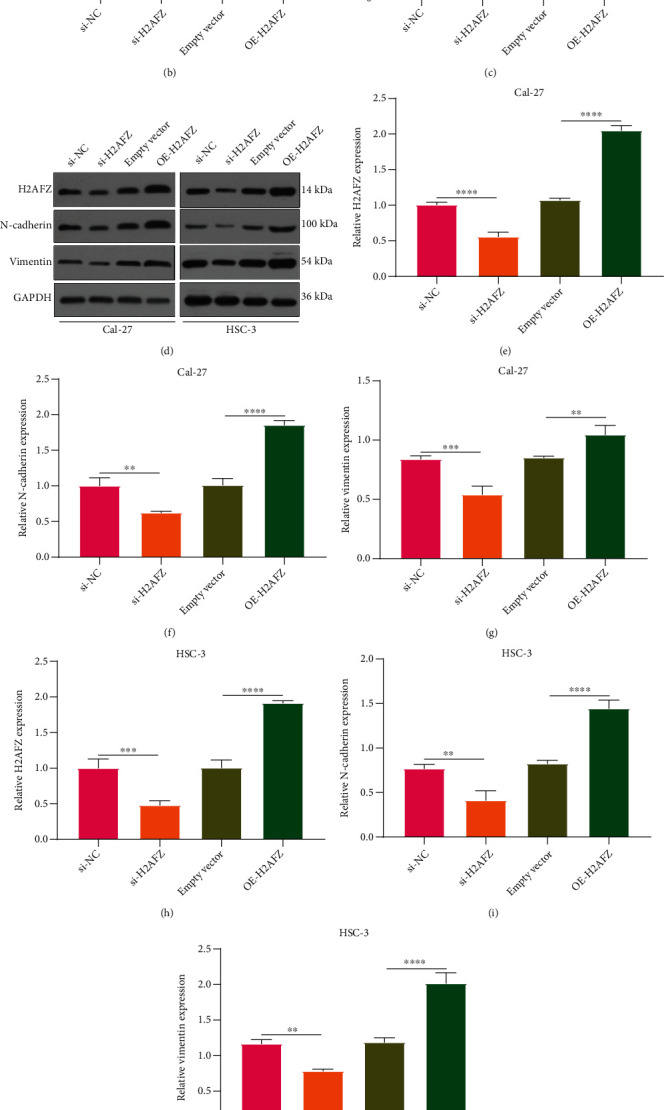
H2AFZ upregulation promotes invasion and EMT process in OSCC cells. (a–c) Transwell assay of the invasive ability of H2AFZ in Cal-27 as well as HSC-3 cells with si-H2AFZ and H2AFZ overexpression transfections. (d) Representative images of western blot. Western blot for quantifying the expression of (e) H2AFZ, (f) N-cadherin, and (g) Vimentin in Cal-27 cells after transfection with si-H2AFZ and H2AFZ overexpression. Western blots of the expressions of (h) H2AFZ, (i) N-cadherin, and (j) Vimentin in HSC-3 cells under transfection with si-H2AFZ and H2AFZ overexpression. (k) Morphology of Cal-27 as well as HSC-3 cells with si-H2AFZ and H2AFZ overexpression transfections. ^∗∗^*p* < 0.01; ^∗∗∗^*p* < 0.001; ^∗∗∗∗^*p* < 0.0001.

Transwell assays were performed for observing invasive ability of OSCC cells. In comparison to si-NC, the number of invasive Cal-27 as well as HSC-3 cells with si-H2AFZ transfection was markedly decreased (Figures 10(a)–10(c)). Inversely, pcDNA3.1-H2AFZ transfection significantly enhanced invasive ability of Cal-27 as well as HSC-3 cells in comparison to empty vector. Western blot showed that H2AFZ protein was distinctly decreased by si-H2AFZ as well as was distinctly increased by pcDNA3.1-H2AFZ in Cal-27 cells (Figures 10(d) and 10(e)). Also, EMT-related proteins were detected by western blot. We observed that N-cadherin as well as Vimentin expressions were distinctly lowered in Cal-27 cells transfected with si-H2AFZ (Figures 10(f) and 10(g)). However, their expressions were markedly elevated in Cal-27 cells under transfection with pcDNA3.1-H2AFZ. The similar findings were confirmed in HSC-3 cells (Figures 10(h)–10(j)). We further investigated the morphology changes of Cal-27 as well as HSC-3 cells after si-H2AFZ or pcDNA3.1-H2AFZ transfections. Our results showed that compared with si-NC, Cal-27 and HSC-3 cells transfected with si-H2AFZ were in the form of epithelioid carcinoma and the cells became loose (Figure 10(k)). Moreover, after transfection with pcDNA3.1-H2AFZ, Cal-27 and HSC-3 cells changed from epithelioid carcinoma to spindle shape, and the cells became loose. Thus, H2AFZ upregulation promoted invasion and EMT in OSCC cells.

## 4. Discussion

Growing evidence suggests that CSCs exert critical roles in OSCC development [20–22]. Targeting OSCC CSCs is greatly important for clinical therapy [23]. Nevertheless, the regulatory mechanism involving CSCs is still ambiguous. Herein, CSCs were quantified by mRNAsi scores. High mRNAsi scores were distinctly associated with undesirable prognosis and tumor microenvironment of OSCC. Functional annotation analyses uncovered the critical roles of the mRNAsi-related genes in cancer progression.

Conventional clinicopathological factors have been utilized for reflecting and prognosticating OSCC progression [24]. TNM represents an effective clinical tool for predicting OSCC prognoses [25]. Moreover, molecular biomarkers could become a beneficial supplement to TNM for improving the predictive accuracy [26]. Also, these molecules exert key functions on OSCC progression as well as serve as novel therapeutic targets [27]. For overcoming the hinder of tumor heterogeneity, a panel of molecular biomarkers displays higher accuracy in reflecting OSCC prognoses in comparison to one [28–30]. By the LASSO method, we developed the mRNAsi-associated signature for OSCC prognosis. Patients with high risk displayed an undesirable prognosis in comparison to those with low risk. ROCs and uni- and multivariate Cox analyses confirmed the well-predictive accuracy of the signature for OSCC prognosis. We attempted to probe the intrinsic mechanisms underlying the mRNAsi-associated signature. Our data demonstrated that base excision repair, cell cycle, DNA replication, mismatch repair, nucleotide excision repair, ribosome, and spliceosome were distinctly activated in high-risk OSCC while B cell receptor, calcium, cell adhesion molecules cam, chemokine-chemokine receptor interaction, ECM receptor interaction, focal adhesion, MAPK, and T cell receptor pathways were markedly activated in low-risk samples, which demonstrated the key role of the mRNAsi-associated signature in OSCC progression.

Nomogram models that integrate a few prognostic factors such as molecules and clinicopathological factors have been widely utilized in clinical oncology for evaluating prognoses [31]. Survival probabilities could be estimated using relatively simple output. In comparison to traditional clinicopathological factors, nomogram models could be accurately predictive of prognoses [32]. Thus, nomogram models are beneficial for clinical decision-making and individualized therapy. This study created the nomogram that combined age, stage, and mRNAsi-associated risk score, which could be accurately predictive of 1-, 3-, and 5-year OS probabilities of OSCC based on ROC, calibration curves, and DCA. Among the 11 mRNAsi-associated genes, CCDC92, GAS1, H2AFZ, IGLV2, KPNA2, NPM3, and TWIST2 were markedly upregulated while CLEC3B displayed decreased expression in OSCC than normal tissues. Previously, H2AFZ expression was distinctly upregulated in hepatocellular carcinoma and associated with undesirable survival outcomes [19]. The upregulation elevated proliferative capacities of hepatocellular carcinoma cells. H2AFZ expression exhibited a distinct correlation to prognosis and recurrence for children with acute lymphoblastic leukemia. We found that H2AFZ upregulation could markedly accelerate proliferation, invasion, and EMT as well as weaken apoptosis for OSCC cells, which was indicative that H2AFZ possessed the potential as a promising therapeutic target for OSCC.

Several limitations of our study should be pointed out. Firstly, the predictive accuracy of the mRNAsi-associated signature will be confirmed in a larger and prospective OSCC cohort. Furthermore, the functions of H2AFZ on OSCC progression should be investigated in vivo. More experiments are required for observing the mechanisms involving H2AFZ in OSCC.

## 5. Conclusion

Collectively, these findings quantified CSCs based on mRNAsi scores, which were closely in relation to survival outcomes and tumor microenvironment. Furthermore, we developed an mRNAsi-related signature that could be applied for prognosis prediction and risk stratification in OSCC. Among the mRNAsi-related genes, H2AFZ was validated to facilitate proliferation, invasion, and EMT as well as suppress apoptosis for OSCC cells. Thus, H2AFZ might become a promising therapeutic target for OSCC.

## Figures and Tables

**Table 1:** Univariate Cox regression analysis identifies prognostic mRNAsi-associated DEGs in OSCC.

Genes	HR	HR.95L	HR.95H	*p*
FMOD	0.820255	0.708671	0.949409	0.007911
H2AFZ	1.395473	1.084497	1.795619	0.009582
KPNA2	1.480072	1.113269	1.967732	0.006967
CCDC92	0.683518	0.517963	0.901988	0.007168
AQP1	0.779629	0.651626	0.932778	0.006519
GAS1	0.785523	0.659077	0.936228	0.007021
NPM3	1.317112	1.072003	1.618263	0.008747
CCL22	0.743591	0.615479	0.898369	0.002134
TSPAN11	0.667706	0.52034	0.856809	0.0015
CLEC3B	0.715459	0.560058	0.91398	0.007365
TWIST2	0.721996	0.589909	0.88366	0.001579
CCR7	0.759354	0.619978	0.930064	0.007797
TPSAB1	0.776882	0.665709	0.90662	0.001355
IGLV2-14	0.911153	0.850275	0.976388	0.008359

## Data Availability

The data used to support the findings of this study are included within the supplementary information files.

## Abbreviations

OSCC: Oral squamous cell carcinoma
CSCs: Cancer stem cells
mRNAsi: mRNA expression-based stemness index
RNA-seq: RNA sequencing
TCGA: The Cancer Genome Atlas
OS: Overall survival
ESTIMATE: Estimation of STromal and Immune cells in MAlignant Tumours using Expression data
DEGs: Differentially expressed genes
FDR: False discovery rate
GO: Gene Ontology
KEGG: Kyoto Encyclopedia of Genes and Genomes
LASSO: Least absolute shrinkage and selection operator
AUC: Area under the curve
ROC: Receiver operator characteristic curve
GSEA: Gene set enrichment analysis
DCA: Decision curve analyses
FBS: Fetal bovine serum
NC: Negative control
RT-qPCR: Real-time quantitative polymerase-chain reaction
CCK-8: Cell counting kit-8.

## Supplementary Materials

Supplementary 1 Supplementary Table 1: clinical features of OSCC patients in TCGA dataset.

Supplementary 2 Supplementary Table 2: the mRNAsi of OSCC patients from TCGA dataset.

Supplementary 3 Supplementary Table 3: the mRNAsi-related DEGs for OSCC.

## References

[1] Ludwig N., Szczepanski M. J., Gluszko A. (2019). CD44(+) tumor cells promote early angiogenesis in head and neck squamous cell carcinoma. *Cancer Letters*.

[2] Qadir F., Lalli A., Dar H. H. (2019). Clinical correlation of opposing molecular signatures in head and neck squamous cell carcinoma. *BMC Cancer*.

[3] Yang Y., Chen D., Liu H., Yang K. (2019). Increased expression of lncRNA CASC9 promotes tumor progression by suppressing autophagy-mediated cell apoptosis via the AKT/mTOR pathway in oral squamous cell carcinoma. *Cell Death & Disease*.

[4] Dan H., Liu S., Liu J. (2020). RACK1 promotes cancer progression by increasing the M2/M1 macrophage ratio via the NF-*κ*B pathway in oral squamous cell carcinoma. *Molecular Oncology*.

[5] Peng Q. S., Cheng Y. N., Zhang W. B., Fan H., Mao Q. H., Xu P. (2020). circRNA_0000140 suppresses oral squamous cell carcinoma growth and metastasis by targeting miR-31 to inhibit Hippo signaling pathway. *Cell Death & Disease*.

[6] Zhang W., Ge H., Jiang Y. (2020). Combinational therapeutic targeting of BRD4 and CDK7 synergistically induces anticancer effects in head and neck squamous cell carcinoma. *Cancer Letters*.

[7] Ma Z., Zhang C., Liu X. (2020). Characterisation of a subpopulation of CD133^+^ cancer stem cells from Chinese patients with oral squamous cell carcinoma. *Scientific Reports*.

[8] Sun L., Xu Y., Zhang X. (2020). Mesenchymal stem cells functionalized sonodynamic treatment for improving therapeutic efficacy and compliance of orthotopic oral cancer. *Advanced Materials*.

[9] Malta T. M., Sokolov A., Gentles A. J. (2018). Machine learning identifies stemness features associated with oncogenic dedifferentiation. *Cell*.

[10] Zhang M., Wang X., Chen X., Guo F., Hong J. (2020). Prognostic value of a stemness index-associated signature in primary lower-grade glioma. *Frontiers in Genetics*.

[11] Bai K. H., He S. Y., Shu L. L. (2020). Identification of cancer stem cell characteristics in liver hepatocellular carcinoma by WGCNA analysis of transcriptome stemness index. *Cancer Medicine*.

[12] Newman A. M., Liu C. L., Green M. R. (2015). Robust enumeration of cell subsets from tissue expression profiles. *Nature Methods*.

[13] Ritchie M. E., Phipson B., Wu D. (2015). limma powers differential expression analyses for RNA-sequencing and microarray studies. *Nucleic Acids Research*.

[14] Yu G., Wang L. G., Han Y., He Q. Y. (2012). clusterProfiler: an R package for comparing biological themes among gene clusters. *OMICS*.

[15] Engebretsen S., Bohlin J. (2019). Statistical predictions with glmnet. *Clinical Epigenetics*.

[16] Subramanian A., Tamayo P., Mootha V. K. (2005). Gene set enrichment analysis: a knowledge-based approach for interpreting genome-wide expression profiles. *Proceedings of the National Academy of Sciences of the United States of America*.

[17] Vickers A. J., Elkin E. B. (2006). Decision curve analysis: a novel method for evaluating prediction models. *Medical Decision Making*.

[18] Qi L., Zhou B., Chen J. (2019). Significant prognostic values of differentially expressed-aberrantly methylated hub genes in breast cancer. *Journal of Cancer*.

[19] Tang S., Huang X., Wang X. (2020). Vital and distinct roles of H2A.Z isoforms in hepatocellular carcinoma. *Oncotargets and Therapy*.

[20] Chen J. H., Wu A. T. H., Bamodu O. A. (2020). Ovatodiolide suppresses oral cancer malignancy by down-regulating exosomal Mir-21/STAT3/*β*-catenin cargo and preventing oncogenic transformation of normal gingival fibroblasts. *Cancers*.

[21] Pai S., Bamodu O. A., Lin Y. K. (2019). CD47-SIRP*α* signaling induces epithelial-mesenchymal transition and cancer stemness and links to a poor prognosis in patients with oral squamous cell carcinoma. *Cell*.

[22] Shriwas O., Priyadarshini M., Samal S. K. (2020). DDX3 modulates cisplatin resistance in OSCC through ALKBH5-mediated m6A-demethylation of FOXM1 and NANOG. *Apoptosis*.

[23] You X., Zhou Z., Chen W., Wei X., Zhou H., Luo W. (2020). MicroRNA-495 confers inhibitory effects on cancer stem cells in oral squamous cell carcinoma through the HOXC6-mediated TGF-*β* signaling pathway. *Stem Cell Research & Therapy*.

[24] De Paz D., Kao H. K., Huang Y., Chang K. P. (2017). Prognostic stratification of patients with advanced oral cavity squamous cell carcinoma. *Current Oncology Reports*.

[25] Almangush A., Mäkitie A. A., Triantafyllou A. (2020). Staging and grading of oral squamous cell carcinoma: an update. *Oral Oncology*.

[26] Xu Y., Xu J., Feng J. (2018). Expression of CLIC1 as a potential biomarker for oral squamous cell carcinoma: a preliminary study. *Oncotargets and Therapy*.

[27] Gao J., Ulekleiv C. H., Halstensen T. S. (2016). Epidermal growth factor (EGF) receptor-ligand based molecular staging predicts prognosis in head and neck squamous cell carcinoma partly due to deregulated EGF- induced amphiregulin expression. *Journal of Experimental & Clinical Cancer Research*.

[28] Li Y., Cao X., Li H. (2020). Identification and validation of novel long non-coding RNA biomarkers for early diagnosis of oral squamous cell carcinoma. *Frontiers in Bioengineering and Biotechnology*.

[29] Wu X., Yao Y., Li Z., Ge H., Wang D., Wang Y. (2020). Identification of a transcriptional prognostic signature from five metabolic pathways in oral squamous cell carcinoma. *Frontiers in Oncology*.

[30] Zeng H., Luo M., Chen L., Ma X., Ma X. (2020). Machine learning analysis of DNA methylation in a hypoxia-immune model of oral squamous cell carcinoma. *International Immunopharmacology*.

[31] Hou C., Cai H., Zhu Y., Huang S., Song F., Hou J. (2020). Development and validation of autophagy-related gene signature and nomogram for predicting survival in oral squamous cell carcinoma. *Frontiers in Oncology*.

[32] Mattavelli D., Lombardi D., Missale F. (2019). Prognostic nomograms in oral squamous cell carcinoma: the negative impact of low neutrophil to lymphocyte ratio. *Frontiers in Oncology*.

