# Supplementary material for: Characterization of Cancer Stem Cell Characteristics and Development of a Prognostic Stemness Index Cell-Related Signature in Oral Squamous Cell Carcinoma
Source: Dis Markers. 2021 Nov 9;2021:1571421. doi: 10.1155/2021/1571421 (PMC8617564; doi:10.1155/2021/1571421)
Supplement: Supplementary 1 — Supplementary Table 1: clinical features of OSCC patients in TCGA dataset. [file 1571421.f1.pdf]

Supplementary table 1. Clinical features of OSCC patients in TCGA dataset.

ID	futime	fustat	age	gender	grade	stage
TCGA-CV-6433	641	0	57	MALE	G3	Stage II
TCGA-BA-A6DE	440	0	70	FEMALE	G2	Stage II
TCGA-MT-A51X	242	0	30	MALE	G1	Stage IVA
TCGA-CV-5439	546	1	62	MALE	G2	Stage IVA
TCGA-MT-A7BN	469	0	74	MALE	G3	Stage IVA
TCGA-D6-A4Z9	539	0	59	MALE	G2	Stage IVA
TCGA-CR-6471	1202	1	58	MALE	G2	Stage IVA
TCGA-BA-5556	179	0	58	FEMALE	G3	Stage II
TCGA-CR-7379	1036	0	78	FEMALE	G2	Stage IVA
TCGA-P3-A6T5	882	1	79	FEMALE	G2	Stage IVA
TCGA-MZ-A7D7	547	0	51	MALE	unknow	Stage IVA
TCGA-CN-4725	1157	0	60	MALE	G2	Stage II
TCGA-CQ-7069	1274	0	77	FEMALE	G2	Stage II
TCGA-CV-5971	701	0	60	MALE	G2	Stage IVA
TCGA-QK-A6IH	508	0	65	FEMALE	G2	Stage IVB
TCGA-CV-A45T	4856	1	64	FEMALE	G3	unknow
TCGA-CR-6472	1050	0	59	MALE	G3	unknow
TCGA-CR-7401	1077	0	64	MALE	G2	Stage I
TCGA-HD-8634	385	1	51	FEMALE	G2	Stage I
TCGA-CQ-A4CB	893	0	59	MALE	G2	Stage III
TCGA-CN-6995	112	1	78	MALE	G2	Stage IVA
TCGA-CV-7435	4680	1	57	FEMALE	G2	Stage IVA
TCGA-CN-6996	530	1	58	FEMALE	G2	Stage IVA
TCGA-CQ-A4CE	785	0	76	FEMALE	G2	Stage II
TCGA-QK-A6II	284	1	52	MALE	G2	Stage IVA
TCGA-CN-4730	817	0	62	MALE	G2	Stage IVA
TCGA-BA-5558	1636	0	65	MALE	G1	unknow
TCGA-CR-7390	1508	0	67	MALE	G2	Stage IVA
TCGA-CQ-A4CI	950	0	73	MALE	G3	Stage III
TCGA-BA-6871	63	0	75	MALE	G2	unknow
TCGA-CX-7086	573	0	53	MALE	G2	Stage III
TCGA-CV-5966	545	1	63	FEMALE	G3	Stage IVA
TCGA-CN-4733	1586	0	61	MALE	G3	Stage III
TCGA-CV-A6JU	110	0	61	FEMALE	G2	Stage IVB
TCGA-CV-7236	144	1	77	FEMALE	G3	Stage IVA
TCGA-CV-7568	927	1	48	FEMALE	GX	Stage IVA
TCGA-CV-A45U	1079	1	59	MALE	G1	Stage IVA
TCGA-QK-A6VB	450	0	66	MALE	G2	Stage IVA
TCGA-WA-A7H4	443	0	69	MALE	G3	Stage II
TCGA-CQ-6223	1428	0	69	MALE	G2	Stage II
TCGA-4P-AA8J	102	0	66	MALE	G2	Stage IVA
TCGA-UF-A7JS	680	1	59	MALE	G2	Stage IVA
TCGA-DQ-5630	1030	0	73	MALE	G2	unknow
TCGA-D6-6516	773	0	69	MALE	G2	Stage I
TCGA-CV-5976	1478	0	50	MALE	G2	Stage IVA
TCGA-CX-7085	321	0	77	FEMALE	G2	Stage I
TCGA-CR-7369	1090	1	59	MALE	G2	Stage IVA
TCGA-D6-6823	701	0	50	MALE	G2	Stage II
TCGA-CV-5436	584	1	65	MALE	G2	Stage IVA
TCGA-CV-6948	1289	1	79	FEMALE	G2	Stage IVB
TCGA-P3-A5QE	1559	0	49	MALE	G2	Stage III

TCGA-CQ-7068	966	0	80	FEMALE	G2	Stage II
TCGA-BB-A5HZ	827	0	65	MALE	G2	Stage IVA
TCGA-HD-7831	361	0	74	MALE	G2	Stage IVA
TCGA-T2-A6WZ	484	1	53	MALE	G2	unknow
TCGA-CV-A468	464	1	42	MALE	G2	Stage IVA
TCGA-UF-A71E	1278	0	63	MALE	G3	Stage IVA
TCGA-CR-7386	1430	0	69	MALE	G1	Stage IVA
TCGA-UP-A6WW	518	0	58	MALE	G2	unknow
TCGA-UF-A719	1663	0	54	MALE	G1	Stage II
TCGA-DQ-7592	516	0	57	MALE	G2	unknow
TCGA-CR-7395	930	0	80	FEMALE	G2	Stage II
TCGA-CV-7100	274	1	66	MALE	G3	Stage III
TCGA-CV-6951	915	1	57	MALE	G2	Stage IVA
TCGA-CN-A6V6	234	0	59	MALE	GX	Stage IVA
TCGA-CQ-5326	89	1	67	MALE	G3	Stage IVA
TCGA-CQ-5334	129	1	87	MALE	G2	Stage IVA
TCGA-CV-5973	2641	0	62	FEMALE	G3	Stage III
TCGA-CV-7253	361	1	58	MALE	G2	Stage IVA
TCGA-CV-7254	1459	1	55	MALE	G3	Stage II
TCGA-CR-7373	889	0	66	MALE	G2	Stage IVA
TCGA-CQ-7063	1750	0	59	FEMALE	G1	Stage I
TCGA-D6-A6EN	439	0	71	MALE	G3	Stage III
TCGA-CV-A45O	851	0	57	MALE	G2	unknow
TCGA-F7-A61S	576	0	62	MALE	G1	Stage III
TCGA-CV-A45P	639	0	82	FEMALE	G2	Stage I
TCGA-CN-4742	397	1	48	FEMALE	G3	Stage IVA
TCGA-KU-A6H8	327	1	41	MALE	G3	Stage IVA
TCGA-RS-A6TO	387	1	82	FEMALE	G2	Stage IVA
TCGA-CN-5364	493	1	55	MALE	G2	Stage IVA
TCGA-CQ-A4CG	430	1	78	FEMALE	G2	Stage III
TCGA-CV-7102	56	1	76	FEMALE	G3	Stage IVA
TCGA-CV-A463	23	1	82	FEMALE	G2	Stage IVA
TCGA-CN-A642	82	1	57	MALE	G3	Stage IVB
TCGA-BA-5152	846	0	56	MALE	G2	Stage IVA
TCGA-CV-A45V	32	1	87	FEMALE	G1	Stage IVA
TCGA-QK-AA3K	253	0	60	MALE	G2	Stage IVA
TCGA-H7-7774	407	0	75	FEMALE	G2	Stage IVA
TCGA-CR-5250	799	0	71	MALE	G3	unknow
TCGA-QK-A8Z9	352	0	56	MALE	G2	Stage IVA
TCGA-F7-A620	543	0	47	MALE	G1	Stage III
TCGA-CR-7367	1440	0	52	MALE	G1	Stage IVA
TCGA-CV-7413	294	1	74	FEMALE	G2	Stage II
TCGA-CN-4741	1268	0	75	MALE	G2	Stage IVA
TCGA-T2-A6X2	987	0	82	MALE	G1	Stage III
TCGA-CV-6952	185	1	65	FEMALE	G1	Stage IVA
TCGA-CN-A6UY	307	0	57	MALE	G2	Stage IVA
TCGA-CQ-5324	1593	0	59	MALE	G2	Stage III
TCGA-CQ-7071	877	0	76	FEMALE	G2	Stage III
TCGA-C9-A480	386	0	45	FEMALE	G1	Stage III
TCGA-CV-A6JN	906	0	53	MALE	G1	unknow
TCGA-UF-A71B	1319	0	50	MALE	G2	Stage IVA
TCGA-CV-6942	4282	0	73	FEMALE	G3	Stage II
TCGA-IQ-A6SH	471	0	55	MALE	G1	Stage III

TCGA-CR-6477	514	0	56	FEMALE	G2	unknow
TCGA-CN-4728	1151	0	56	MALE	G2	Stage IVA
TCGA-CN-A63V	484	0	59	MALE	G2	Stage IVA
TCGA-IQ-7631	1172	0	60	FEMALE	G1	Stage II
TCGA-CQ-A4C9	707	0	56	MALE	G2	Stage III
TCGA-BA-5151	190	0	72	MALE	G1	Stage IVA
TCGA-CQ-6219	479	1	50	FEMALE	G2	Stage IVA
TCGA-CR-6488	379	0	68	FEMALE	G2	Stage II
TCGA-CR-7377	279	1	58	MALE	G3	Stage IVA
TCGA-HD-8224	446	1	63	MALE	G3	Stage IVA
TCGA-CQ-5333	341	1	74	MALE	G3	Stage II
TCGA-D6-8569	770	0	52	MALE	G2	Stage II
TCGA-MZ-A6I9	489	1	68	MALE	G2	unknow
TCGA-C9-A47Z	191	1	72	FEMALE	G1	Stage III
TCGA-F7-A61V	384	0	54	MALE	G1	Stage II
TCGA-HD-A633	421	0	74	MALE	G2	Stage IVA
TCGA-F7-8489	658	0	48	MALE	G1	Stage II
TCGA-F7-A61W	14	0	51	MALE	G2	Stage IVA
TCGA-CV-A465	215	1	24	MALE	G1	Stage III
TCGA-CQ-6228	456	1	71	FEMALE	G2	Stage IVA
TCGA-CQ-6222	1407	0	63	MALE	G2	Stage IVA
TCGA-QK-A64Z	641	1	79	FEMALE	G1	Stage IVA
TCGA-IQ-A61J	1021	0	54	MALE	G1	Stage IVA
TCGA-CV-6954	2002	1	59	MALE	G2	Stage IVA
TCGA-CN-6019	432	0	61	MALE	G2	Stage IVA
TCGA-P3-A5QA	1726	0	41	MALE	G3	Stage I
TCGA-CV-7446	1093	1	66	MALE	G2	Stage IVA
TCGA-CR-7393	993	0	26	MALE	G2	Stage III
TCGA-CN-6017	629	0	55	MALE	G2	Stage IVA
TCGA-P3-A6T0	578	0	47	FEMALE	G2	Stage IVA
TCGA-DQ-5625	1133	1	52	FEMALE	G2	unknow
TCGA-CQ-6227	129	1	77	MALE	G2	Stage IVA
TCGA-P3-A6T3	577	1	49	MALE	G2	Stage IVA
TCGA-CQ-7065	1335	0	40	MALE	G2	Stage II
TCGA-CN-4731	993	0	63	FEMALE	G3	Stage IVA
TCGA-CV-A6K0	606	0	58	MALE	G3	Stage I
TCGA-CQ-A4CD	998	0	69	MALE	G3	Stage IVA
TCGA-CN-5359	377	1	59	MALE	G2	Stage IVA
TCGA-CQ-6218	1253	0	52	FEMALE	G2	Stage IVA
TCGA-UF-A71A	86	1	67	MALE	G1	Stage IVA
TCGA-CV-A6K2	317	1	79	MALE	G1	Stage IVA
TCGA-T2-A6WX	209	1	73	FEMALE	G1	unknow
TCGA-CQ-6225	403	1	65	MALE	G2	Stage III
TCGA-P3-A6T7	487	1	55	MALE	G2	Stage IVA
TCGA-CX-A4AQ	1555	0	56	MALE	G3	Stage IVA
TCGA-CV-6940	804	1	80	FEMALE	G2	Stage III
TCGA-CR-7376	972	0	83	MALE	G2	Stage III
TCGA-CV-6950	459	1	64	MALE	G2	Stage IVA
TCGA-CQ-A4C7	353	1	88	MALE	G3	Stage III
TCGA-MT-A67A	914	0	85	FEMALE	G2	Stage I
TCGA-CN-4729	392	0	73	MALE	G2	Stage III
TCGA-CV-7099	243	1	85	FEMALE	G2	Stage II
TCGA-CV-A45R	5480	0	46	MALE	G1	Stage III

TCGA-CN-4740	811	0	79	FEMALE	G2	Stage IVA
TCGA-CX-7082	11	1	82	MALE	G2	Stage IVA
TCGA-CR-7397	754	0	44	MALE	G2	Stage IVA
TCGA-QK-A6IG	222	1	69	MALE	G2	Stage III
TCGA-CV-6441	292	1	60	MALE	G3	Stage III
TCGA-CQ-6224	1350	0	52	MALE	G3	Stage IVA
TCGA-CV-5970	406	1	59	MALE	G2	Stage IVA
TCGA-CV-7180	327	1	34	MALE	G2	Stage II
TCGA-BA-A6DJ	407	1	62	MALE	G2	Stage IVA
TCGA-BA-6873	122	0	28	MALE	G2	Stage IVA
TCGA-UF-A7JA	1894	0	66	FEMALE	G2	Stage IVA
TCGA-CV-A6JO	197	1	69	MALE	G2	Stage IVA
TCGA-CV-A6JE	1075	0	78	MALE	G2	unknow
TCGA-CN-6998	46	0	53	MALE	G2	Stage IVA
TCGA-BA-A6D8	850	0	59	MALE	G2	Stage IVA
TCGA-CX-7219	1045	0	47	MALE	G2	Stage IVA
TCGA-D6-A6EM	232	0	65	FEMALE	G2	Stage III
TCGA-DQ-7588	427	1	66	MALE	G3	unknow
TCGA-CR-7391	913	0	36	FEMALE	G1	Stage I
TCGA-QK-A8Z7	198	0	59	MALE	unknow	Stage IVA
TCGA-CV-7414	14	1	78	MALE	G2	Stage IVA
TCGA-BA-5557	242	0	41	FEMALE	G2	Stage III
TCGA-CN-6018	580	1	85	FEMALE	G2	Stage IVA
TCGA-CR-6492	479	0	78	MALE	G3	Stage III
TCGA-CQ-6221	1000	0	79	MALE	G3	unknow
TCGA-F7-A624	378	0	73	MALE	G3	Stage II
TCGA-CV-A464	1722	0	48	MALE	G2	Stage III
TCGA-UF-A7JD	735	0	71	MALE	G3	Stage IVA
TCGA-DQ-5624	1778	0	43	FEMALE	G2	unknow
TCGA-CN-A498	443	0	61	FEMALE	G1	Stage III
TCGA-HL-7533	1057	0	65	MALE	G3	unknow
TCGA-CR-6493	282	1	69	MALE	G2	Stage IVA
TCGA-CR-6491	693	0	60	MALE	G2	Stage IVA
TCGA-CV-A45X	198	1	47	MALE	G2	Stage IVA
TCGA-CN-4736	395	1	70	FEMALE	G2	unknow
TCGA-CQ-A4C6	1353	0	63	MALE	G2	Stage II
TCGA-CQ-7072	1950	0	51	MALE	G3	Stage II
TCGA-D6-6827	568	0	55	FEMALE	G3	Stage I
TCGA-IQ-A61E	1147	0	55	FEMALE	G2	Stage III
TCGA-BA-A6DG	69	1	49	MALE	G2	unknow
TCGA-QK-A6IJ	387	0	71	MALE	G3	Stage III
TCGA-CV-A6JT	852	0	65	MALE	G2	Stage II
TCGA-CQ-A4CH	371	0	58	MALE	G2	Stage II
TCGA-CV-A45Q	5152	1	69	FEMALE	G1	unknow
TCGA-BA-A6DB	216	0	24	FEMALE	G1	Stage I
TCGA-CN-6013	628	0	56	MALE	G3	Stage IVA
TCGA-CR-7372	759	0	45	MALE	G1	Stage I
TCGA-CQ-5327	1660	0	61	FEMALE	G2	Stage IVA
TCGA-D6-6825	491	0	73	MALE	G2	Stage I
TCGA-BA-6872	384	1	47	MALE	G2	unknow
TCGA-HD-8314	670	0	58	MALE	G4	Stage III
TCGA-CQ-5330	1393	0	69	FEMALE	G3	Stage IVA
TCGA-QK-A652	357	0	60	MALE	G2	Stage III

TCGA-BB-8601	624	0	84	MALE	G2	Stage III
TCGA-CN-4737	625	0	19	MALE	G2	Stage IVA
TCGA-P3-A6T6	395	1	53	MALE	G3	Stage IVA
TCGA-CV-6436	1899	0	62	MALE	G1	Stage IVA
TCGA-BA-7269	1273	0	61	MALE	G1	Stage III
TCGA-CN-5373	1584	0	55	FEMALE	G1	Stage I
TCGA-D6-A4ZB	376	0	61	MALE	G2	Stage III
TCGA-CN-6011	933	0	57	MALE	G3	Stage IVA
TCGA-BB-A5HU	782	0	47	MALE	G2	Stage IVA
TCGA-CV-7263	560	1	64	MALE	G1	Stage II
TCGA-MT-A67D	56	0	55	MALE	G2	Stage II
TCGA-D6-A6EO	435	0	44	MALE	G2	Stage IVA
TCGA-CR-7382	796	0	49	MALE	G2	Stage IVA
TCGA-CV-7407	1081	1	67	FEMALE	G2	Stage II
TCGA-DQ-5631	548	1	52	MALE	G3	unknow
TCGA-CN-5369	1	1	90	FEMALE	G3	Stage IVA
TCGA-BA-A6DD	173	1	44	MALE	G2	Stage IVA
TCGA-CV-6955	334	1	74	FEMALE	G3	Stage II
TCGA-IQ-A61G	360	0	57	MALE	G2	Stage IVA
TCGA-DQ-7591	622	0	62	MALE	G4	unknow
TCGA-CV-A6JY	646	0	69	MALE	G1	Stage IVA
TCGA-CQ-6229	1179	0	61	MALE	G2	Stage II
TCGA-CQ-6220	985	1	69	MALE	G2	Stage III
TCGA-CV-7411	2717	1	64	FEMALE	G1	Stage IVA
TCGA-CR-7394	1346	0	70	MALE	G2	Stage IVA
TCGA-CN-A49A	391	0	60	MALE	G2	Stage IVB
TCGA-P3-A6T8	400	0	54	MALE	G3	Stage IVA
TCGA-IQ-A61H	1138	0	76	MALE	G2	Stage II
TCGA-CQ-5329	1422	0	46	FEMALE	G2	Stage II
TCGA-D6-6515	403	1	82	FEMALE	G3	Stage II
TCGA-CN-6994	735	0	67	MALE	G2	Stage IVA
TCGA-CR-7380	606	1	58	MALE	G2	Stage IVB
TCGA-UF-A7JO	631	1	79	FEMALE	G2	Stage IVA
TCGA-CV-7095	572	1	87	FEMALE	G2	Stage II
TCGA-CV-A6JZ	714	0	68	MALE	G2	Stage II
TCGA-CV-7090	5252	0	39	MALE	G2	Stage II
TCGA-CR-6484	354	0	67	FEMALE	G2	Stage IVA
TCGA-CN-6020	205	1	58	MALE	G2	Stage III
TCGA-CV-6945	366	1	41	MALE	G2	Stage IVA
TCGA-BA-4077	1134	1	45	FEMALE	G2	Stage IVA
TCGA-CQ-5331	1399	0	73	FEMALE	G2	unknow
TCGA-CN-5358	261	1	60	MALE	G2	Stage II
TCGA-BA-A4IG	855	0	77	MALE	G3	unknow
TCGA-KU-A66T	552	0	53	FEMALE	G2	Stage IVA
TCGA-P3-A5QF	330	1	49	MALE	G2	Stage IVA
TCGA-T3-A92N	95	1	79	MALE	G3	Stage IVA
TCGA-HD-7832	350	0	52	MALE	G2	Stage IVA
TCGA-P3-A6T2	2165	0	45	MALE	G2	Stage IVA
TCGA-IQ-7632	441	0	68	FEMALE	G1	Stage IVA
TCGA-F7-A50G	616	0	66	MALE	G1	Stage III
TCGA-CR-7392	1425	0	67	FEMALE	G1	Stage IVA
TCGA-CQ-5323	1466	0	82	MALE	G2	Stage I
TCGA-CV-6953	1641	1	80	FEMALE	G1	Stage III

TCGA-P3-A6T4	62	1	54	MALE	G2	Stage IVA
TCGA-CV-6003	1665	0	50	FEMALE	G2	Stage III
TCGA-CV-5979	1315	0	26	MALE	G2	Stage IVA
TCGA-BB-4224	278	0	52	MALE	G2	Stage IVA
TCGA-CV-6941	342	1	51	MALE	G2	Stage III
TCGA-CN-6024	224	0	66	MALE	G2	Stage IVA
TCGA-CN-6016	594	0	64	MALE	G2	Stage IVA
TCGA-BB-A6UO	268	1	61	FEMALE	G2	Stage IVA
TCGA-CR-7368	1245	0	54	MALE	G2	Stage IVA
TCGA-CQ-5325	654	1	65	MALE	G2	Stage I
TCGA-BB-4225	146	0	73	MALE	G3	unknow
TCGA-CV-A6JD	182	1	82	FEMALE	G3	unknow
TCGA-CV-7428	1671	1	47	MALE	G2	Stage IVA
TCGA-CV-7429	107	1	55	MALE	G3	Stage IVA
TCGA-CV-7427	4760	1	73	FEMALE	G1	Stage II
TCGA-UF-A7JT	993	1	72	FEMALE	G3	Stage IVA
TCGA-MT-A67F	384	0	60	FEMALE	G2	Stage IVA
TCGA-CR-7365	1191	0	60	MALE	G2	Stage IVA
TCGA-F7-A50J	585	0	67	FEMALE	G2	Stage III
TCGA-CN-5370	259	1	78	MALE	G3	Stage III
TCGA-CV-7104	393	1	61	FEMALE	G2	Stage IVA
TCGA-HD-A4C1	11	0	41	FEMALE	G1	Stage IVA
TCGA-UF-A7JC	546	1	42	MALE	G1	Stage IVA
TCGA-CN-5367	352	1	60	FEMALE	G2	Stage IVA
TCGA-BA-4074	462	1	69	MALE	G3	Stage IVA
TCGA-BB-4228	558	0	50	MALE	GX	Stage III
TCGA-H7-8502	458	0	50	MALE	G2	Stage IVA
TCGA-CN-4726	142	1	68	MALE	G2	Stage IVA
TCGA-CV-5442	2327	0	76	FEMALE	G3	Stage IVA
TCGA-CV-5977	1840	0	66	MALE	G2	Stage IVA
TCGA-CV-6937	624	1	71	MALE	G2	Stage II
TCGA-CQ-5332	317	1	87	MALE	G2	Stage III
TCGA-H7-8501	461	0	54	MALE	GX	Stage IVA

T	M	N
T2	unknow	N0
T2	M0	N0
T1	M0	N2b
T3	unknow	N2b
unknow	unknow	unknow
T2	M0	N2
T4a	M0	N1
T2	unknow	N0
T4a	M0	N2b
T4a	MX	N0
T3	MX	N2b
T2	unknow	N0
T2	M0	N0
T4a	unknow	N2a
T4b	M0	N2b
T1	M0	N0
unknow	unknow	unknow
T1	M0	N0
T1	MX	N0
T1	M0	N1
T4a	unknow	N0
T3	unknow	N2b
T3	unknow	N2b
T2	M0	N0
T4a	M0	N2c
T4a	unknow	N1
TX	unknow	NX
T4a	M0	N0
T2	M0	N1
TX	unknow	NX
T3	unknow	N1
T4a	unknow	N1
T1	unknow	N1
T4b	M0	N0
T3	unknow	N2c
T4a	unknow	NX
T4	M0	N2b
T4a	M0	N0
T2	unknow	N0
T2	unknow	N0
T2	MX	N2c
T4a	M0	N2b
TX	unknow	NX
T1	unknow	N0
T4a	unknow	N2b
T1	unknow	N0
T4a	M0	N2b
T2	unknow	N0
T3	unknow	N2b
T4a	unknow	N3
T2	MX	N1

T2	unknow	N0
T4a	M0	N1
T2	unknow	N2
T3	unknow	N2b
T4a	M0	N2a
T4a	M0	N0
T4a	M0	N2c
unknow	unknow	unknow
T2	M0	N0
TX	unknow	NX
T2	M0	N0
T2	unknow	N1
T4a	unknow	N2c
T2	MX	N2b
T4a	unknow	N2c
T3	unknow	N2b
T3	unknow	N1
T4a	unknow	N0
T2	unknow	NX
T4a	M0	N1
T1	M0	NX
T2	M0	N1
unknow	unknow	unknow
T3	M0	N0
T1	M0	N0
T4a	M0	N2b
T2	M0	N2b
T4	M0	N2c
T4a	M0	N2c
T3	M0	N0
T3	unknow	N2
T4a	M0	N0
T4a	M0	N3
T4a	M0	N0
T4	M0	N0
T3	MX	N2b
T4a	unknow	N0
TX	unknow	NX
T4a	M0	N2b
T3	M0	N1
T4a	M0	N1
T2	unknow	N0
T4a	M0	N0
T3	MX	N0
T3	unknow	N2b
T3	MX	N2b
T3	unknow	N0
T2	M0	N1
T3	M0	N0
unknow	unknow	unknow
T4	M0	N0
T2	unknow	NX
T2	M0	N1

unknow	unknow	unknow
T3	unknow	N2b
T4a	MX	N0
T2	unknow	N0
T2	M0	N1
T4a	unknow	N0
T3	unknow	N2a
T2	unknow	N0
T4a	M0	N2c
T3	M0	N2c
T2	unknow	N0
T2	M0	N0
unknow	unknow	unknow
T2	M0	N1
T2	M0	N0
T4a	MX	N0
T2	M0	N0
T2	M0	N2b
T3	M0	N0
T1	unknow	N2b
T2	unknow	N2b
T4	MX	NX
T2	M0	N2b
T4a	unknow	NX
T4a	unknow	N0
T1	MX	NX
T2	unknow	N2b
T1	M0	N1
T3	unknow	N2b
T4a	MX	N0
TX	unknow	NX
T3	unknow	N2c
T3	MX	N2
T2	unknow	N0
T4a	unknow	N2c
T1	M0	N0
T4a	M0	N0
T4a	M0	N2b
T3	unknow	N2b
T4a	M0	N2c
T4	M0	N0
unknow	unknow	unknow
T3	unknow	N0
T3	MX	N2b
T2	M0	N2b
T2	unknow	N1
T2	M0	N1
T3	unknow	N2c
T3	M0	N1
T1	MX	N0
T2	unknow	N1
T2	unknow	NX
T2	M0	N1

T4a	M0	N0
T2	unknow	N2b
T3	M0	N2b
T2	M0	N1
T3	unknow	N0
T2	unknow	N2b
T4a	unknow	N2b
T2	unknow	NX
T4a	M0	N2c
T4a	unknow	N2b
T4a	M0	N1
T3	M0	N2c
unknow	unknow	unknow
T3	unknow	N2b
T4a	M0	N2c
T4a	unknow	N2c
T2	M0	N1
TX	unknow	NX
T1	M0	N0
T4a	M0	N2c
T4a	unknow	N2b
T1	unknow	N1
T4a	unknow	N2b
T2	M0	N1
T2	unknow	NX
T2	M0	NX
T3	M0	N0
T4	M0	N2b
TX	unknow	NX
T3	MX	N0
unknow	unknow	unknow
T3	unknow	N2b
T4a	M0	N2b
T2	M0	N2b
T1	unknow	NX
T2	M0	N0
T2	M0	N0
T1	unknow	N0
T3	MX	N0
TX	MX	NX
T2	M0	N1
T2	M0	N0
T2	M0	N0
unknow	unknow	unknow
T1	M0	N0
T4a	unknow	N2b
T1	M0	N0
T3	unknow	N2c
T1	unknow	N0
TX	unknow	NX
T1	unknow	N1
T3	unknow	N2b
T1	MX	N1

T3	MX	N1
T2	unknow	N2b
T4a	MX	NX
T4a	unknow	N0
T2	unknow	N1
T1	unknow	N0
T3	M0	N0
T4a	unknow	N0
T4	M0	N2b
T2	unknow	NX
T2	M0	N0
T4a	M0	N0
T2	M0	N2c
T2	unknow	NX
TX	unknow	NX
T4a	M0	N0
T4a	M0	N2c
T2	unknow	NX
T4a	MX	N2c
TX	unknow	NX
T4a	M0	N0
T2	unknow	N0
T3	unknow	N0
T4a	unknow	N0
T4a	M0	N0
T4b	MX	N0
T4a	MX	N2
T2	MX	N0
T2	unknow	N0
T2	unknow	N0
T4a	unknow	N0
T4b	unknow	N0
T4a	M0	N0
T2	unknow	NX
T2	M0	N0
T2	unknow	NX
T4a	M0	N2
T2	unknow	N1
T4a	unknow	N2
T4a	M0	N0
T2	unknow	NX
T2	unknow	N0
T1	MX	NX
T4	MX	N0
T4	MX	N2b
T2	MX	N2c
T4a	unknow	N0
T3	MX	N2b
T4a	M0	N0
T3	M0	N1
T3	M0	N2b
T1	unknow	N0
T3	unknow	N0

T4a	MX	N1
T2	unknow	N1
T2	unknow	N2b
T2	unknow	N2b
T3	unknow	N0
T4a	M0	N2c
T4a	unknow	N1
T4a	MX	N2b
T4a	M0	N1
T1	unknow	N0
TX	unknow	NX
unknow	unknow	unknow
T4a	unknow	N2b
T4a	unknow	N2b
T2	unknow	NX
T4a	M0	N0
T4	MX	N0
T4a	M0	N0
T3	M0	N0
T3	M0	N1
T2	unknow	N2b
T4a	MX	N2b
T3	M0	N2b
T4a	M0	N2b
T2	M0	N2c
T3	unknow	NX
T4a	M0	N2b
T3	unknow	N2b
T4a	unknow	N2b
T3	unknow	N2b
T2	unknow	N0
T3	unknow	N0
T4a	M0	N1
