# Supplementary material for: Characterization of Cancer Stem Cell Characteristics and Development of a Prognostic Stemness Index Cell-Related Signature in Oral Squamous Cell Carcinoma
Source: Dis Markers. 2021 Nov 9;2021:1571421. doi: 10.1155/2021/1571421 (PMC8617564; doi:10.1155/2021/1571421)
Supplement: Supplementary 2 — Supplementary Table 2: the mRNAsi of OSCC patients from TCGA dataset. [file 1571421.f2.pdf]

Supplementary table 2. The mRNAsi of OSCC patients from TCGA dataset.

ID	futime	fustat	mRNAsi	Group
TCGA-4P-AA8J	0.279452055	0	0.248501314	Low
TCGA-BA-4074	1.265753425	1	0.482600686	High
TCGA-BA-5151	0.520547945	0	0.414790184	High
TCGA-BA-5152	2.317808219	0	0.446315722	High
TCGA-BA-5556	0.490410959	0	0.420790696	High
TCGA-BA-5557	0.663013699	0	0.284066202	Low
TCGA-BA-5558	4.482191781	0	0.463803031	High
TCGA-BA-6871	0.17260274	0	0.421300583	High
TCGA-BA-6872	1.052054795	1	0.409216338	High
TCGA-BA-6873	0.334246575	0	0.449307057	High
TCGA-BA-7269	3.487671233	0	0.381771922	High
TCGA-BA-A6D8	2.328767123	0	0.457323605	High
TCGA-BA-A6DB	0.591780822	0	0.319091299	Low
TCGA-BA-A6DD	0.473972603	1	0.401408946	High
TCGA-BA-A6DE	1.205479452	0	0.477370936	High
TCGA-BA-A6DG	0.189041096	1	0.181420174	Low
TCGA-BA-A6DJ	1.115068493	1	0.417325265	High
TCGA-BB-4224	0.761643836	0	0.516092804	High
TCGA-BB-4225	0.4	0	0.464270494	High
TCGA-BB-4228	1.528767123	0	0.466980536	High
TCGA-BB-8601	1.709589041	0	0.509804919	High
TCGA-BB-A5HU	2.142465753	0	0.33592277	Low
TCGA-BB-A5HZ	2.265753425	0	0.468802037	High
TCGA-BB-A6UO	0.734246575	1	0.38818362	High
TCGA-C9-A47Z	0.523287671	1	0.42049737	High
TCGA-C9-A480	1.057534247	0	0.40445427	High
TCGA-CN-4725	3.169863014	0	0.431896882	High
TCGA-CN-4726	0.389041096	1	0.341255452	Low
TCGA-CN-4728	3.153424658	0	0.231948409	Low
TCGA-CN-4729	1.073972603	0	0.389659043	High
TCGA-CN-4730	2.238356164	0	0.592758542	High
TCGA-CN-4731	2.720547945	0	0.351175751	Low
TCGA-CN-4733	4.345205479	0	0.319579673	Low
TCGA-CN-4734	2.076712329	0	0.398018892	High
TCGA-CN-4736	1.082191781	1	0.288511405	Low
TCGA-CN-4737	1.712328767	0	0.267817758	Low
TCGA-CN-4740	2.221917808	0	0.308002688	Low
TCGA-CN-4741	3.473972603	0	0.451789526	High
TCGA-CN-4742	1.087671233	1	0.325254842	Low
TCGA-CN-5358	0.715068493	1	0.283068408	Low
TCGA-CN-5359	1.032876712	1	0.383073403	High
TCGA-CN-5364	1.350684932	1	0.456904431	High
TCGA-CN-5367	0.964383562	1	0.471430162	High
TCGA-CN-5369	0.002739726	1	0.395206641	High
TCGA-CN-5370	0.709589041	1	0.345929983	Low
TCGA-CN-5373	4.339726027	0	0.292515321	Low
TCGA-CN-6011	2.556164384	0	0.367672528	High
TCGA-CN-6013	1.720547945	0	0.355989637	Low
TCGA-CN-6016	1.62739726	0	0.141820934	Low
TCGA-CN-6017	1.723287671	0	0.262573659	Low
TCGA-CN-6018	1.589041096	1	0.376492473	High

TCGA-CN-6019	1.183561644	0	0.165547097	Low
TCGA-CN-6020	0.561643836	1	0.376420375	High
TCGA-CN-6024	0.61369863	0	0.38160446	High
TCGA-CN-6994	2.01369863	0	0.325693122	Low
TCGA-CN-6995	0.306849315	1	0.45660008	High
TCGA-CN-6996	1.452054795	1	0.380533156	High
TCGA-CN-6998	0.126027397	0	0.382467857	High
TCGA-CN-A498	1.21369863	0	0.37683874	High
TCGA-CN-A49A	1.071232877	0	0.315668747	Low
TCGA-CN-A63V	1.326027397	0	0.299339348	Low
TCGA-CN-A642	0.224657534	1	0.420310662	High
TCGA-CN-A6UY	0.84109589	0	0.494321526	High
TCGA-CN-A6V6	0.64109589	0	0.433988492	High
TCGA-CQ-5323	4.016438356	0	0.506750702	High
TCGA-CQ-5324	4.364383562	0	0.379479067	High
TCGA-CQ-5325	1.791780822	1	0.371842777	High
TCGA-CQ-5326	0.243835616	1	0.443893533	High
TCGA-CQ-5327	4.547945205	0	0.456935725	High
TCGA-CQ-5329	3.895890411	0	0.402710186	High
TCGA-CQ-5330	3.816438356	0	0.343667304	Low
TCGA-CQ-5331	3.832876712	0	0.466562406	High
TCGA-CQ-5332	0.868493151	1	0.421028633	High
TCGA-CQ-5333	0.934246575	1	0.366237455	High
TCGA-CQ-5334	0.353424658	1	0.404002581	High
TCGA-CQ-6218	3.432876712	0	0.401069958	High
TCGA-CQ-6219	1.312328767	1	0.417014506	High
TCGA-CQ-6220	2.698630137	1	0.462011917	High
TCGA-CQ-6222	3.854794521	0	0.352079171	Low
TCGA-CQ-6223	3.912328767	0	0.439196094	High
TCGA-CQ-6224	3.698630137	0	0.243180708	Low
TCGA-CQ-6225	1.104109589	1	0.531438364	High
TCGA-CQ-6227	0.353424658	1	0.298803154	Low
TCGA-CQ-6228	1.249315068	1	0.452205836	High
TCGA-CQ-6229	3.230136986	0	0.405267654	High
TCGA-CQ-7063	4.794520548	0	0.389070648	High
TCGA-CQ-7065	3.657534247	0	0.370773559	High
TCGA-CQ-7068	2.646575342	0	0.427317219	High
TCGA-CQ-7069	3.490410959	0	0.265494047	Low
TCGA-CQ-7071	2.402739726	0	0.38769463	High
TCGA-CQ-7072	5.342465753	0	0.219006854	Low
TCGA-CQ-A4C6	3.706849315	0	0.369735504	High
TCGA-CQ-A4C9	1.936986301	0	0.469426215	High
TCGA-CQ-A4CB	2.446575342	0	0.421597682	High
TCGA-CQ-A4CE	2.150684932	0	0.457247927	High
TCGA-CQ-A4CG	1.178082192	1	0.384015874	High
TCGA-CQ-A4CH	1.016438356	0	0.391365433	High
TCGA-CQ-A4CI	2.602739726	0	0.521525996	High
TCGA-CR-5250	2.189041096	0	0.380824519	High
TCGA-CR-6471	3.293150685	1	0.406171906	High
TCGA-CR-6472	2.876712329	0	0.505887243	High
TCGA-CR-6477	1.408219178	0	0.34018274	Low
TCGA-CR-6484	0.969863014	0	0.385274127	High
TCGA-CR-6488	1.038356164	0	0.256991289	Low

TCGA-CR-6491	1.898630137	0	0.409621405	High
TCGA-CR-6492	1.312328767	0	0.387398012	High
TCGA-CR-6493	0.77260274	1	0.494508179	High
TCGA-CR-7365	3.263013699	0	0.355152717	Low
TCGA-CR-7367	3.945205479	0	0.354146451	Low
TCGA-CR-7368	3.410958904	0	0.470353871	High
TCGA-CR-7369	2.98630137	1	0.414267362	High
TCGA-CR-7372	2.079452055	0	0.354244645	Low
TCGA-CR-7373	2.435616438	0	0.465074684	High
TCGA-CR-7376	2.663013699	0	0.239109959	Low
TCGA-CR-7377	0.764383562	1	0.316003226	Low
TCGA-CR-7379	2.838356164	0	0.436914888	High
TCGA-CR-7380	1.660273973	1	0.405425798	High
TCGA-CR-7382	2.180821918	0	0.291687793	Low
TCGA-CR-7386	3.917808219	0	0.238367777	Low
TCGA-CR-7390	4.131506849	0	0.454297873	High
TCGA-CR-7391	2.501369863	0	0.308018368	Low
TCGA-CR-7392	3.904109589	0	0.305423182	Low
TCGA-CR-7393	2.720547945	0	0.254382301	Low
TCGA-CR-7394	3.687671233	0	0.340629753	Low
TCGA-CR-7395	2.547945205	0	0.391050099	High
TCGA-CR-7397	2.065753425	0	0.256485765	Low
TCGA-CR-7401	2.950684932	0	0.292564651	Low
TCGA-CV-5436	1.6	1	0.42329328	High
TCGA-CV-5439	1.495890411	1	0.429887189	High
TCGA-CV-5442	6.375342466	0	0.545508652	High
TCGA-CV-5966	1.493150685	1	0.516466113	High
TCGA-CV-5970	1.112328767	1	0.344088265	Low
TCGA-CV-5971	1.920547945	0	0.238394734	Low
TCGA-CV-5973	7.235616438	0	0.339633745	Low
TCGA-CV-5976	4.049315068	0	0.353666647	Low
TCGA-CV-5977	5.04109589	0	0.471748558	High
TCGA-CV-5979	3.602739726	0	0.339755522	Low
TCGA-CV-6003	4.561643836	0	0.478481221	High
TCGA-CV-6433	1.756164384	0	0.501382779	High
TCGA-CV-6436	5.202739726	0	0.48719423	High
TCGA-CV-6441	0.8	1	0.460132254	High
TCGA-CV-6933	7.509589041	1	0.400037397	High
TCGA-CV-6934	0.178082192	1	0.195570252	Low
TCGA-CV-6936	0.454794521	1	0.479814438	High
TCGA-CV-6937	1.709589041	1	0.358073552	Low
TCGA-CV-6938	0.394520548	1	0.460124886	High
TCGA-CV-6939	1.824657534	1	0.39640835	High
TCGA-CV-6940	2.202739726	1	0.407239435	High
TCGA-CV-6941	0.936986301	1	0.393454809	High
TCGA-CV-6942	11.73150685	0	0.383892753	High
TCGA-CV-6943	1.649315068	1	0.261716145	Low
TCGA-CV-6945	1.002739726	1	0.36551087	High
TCGA-CV-6948	3.531506849	1	0.529834459	High
TCGA-CV-6950	1.257534247	1	0.36648052	High
TCGA-CV-6951	2.506849315	1	0.378250379	High
TCGA-CV-6952	0.506849315	1	0.384416193	High
TCGA-CV-6953	4.495890411	1	0.416700326	High

TCGA-CV-6954	5.484931507	1	0.409537264	High
TCGA-CV-6955	0.915068493	1	0.4639956	High
TCGA-CV-6956	0.594520548	1	0.464316863	High
TCGA-CV-6959	0.701369863	1	0.374800324	High
TCGA-CV-6960	2.361643836	1	0.427184818	High
TCGA-CV-6961	0.208219178	1	0.352983435	Low
TCGA-CV-7090	14.3890411	0	0.354079533	Low
TCGA-CV-7091	9.263013699	0	0.423574165	High
TCGA-CV-7095	1.567123288	1	0.41283629	High
TCGA-CV-7097	1.054794521	1	0.438081501	High
TCGA-CV-7099	0.665753425	1	0.464860426	High
TCGA-CV-7100	0.750684932	1	0.437536865	High
TCGA-CV-7102	0.153424658	1	0.380334812	High
TCGA-CV-7103	4.35890411	1	0.253101224	Low
TCGA-CV-7104	1.076712329	1	0.318149766	Low
TCGA-CV-7178	5.934246575	1	0.402488982	High
TCGA-CV-7180	0.895890411	1	0.480559502	High
TCGA-CV-7183	10.90684932	0	0.380884415	High
TCGA-CV-7235	6.430136986	0	0.346063259	Low
TCGA-CV-7236	0.394520548	1	0.336949228	Low
TCGA-CV-7238	7.471232877	0	0.324248868	Low
TCGA-CV-7252	0.41369863	1	0.397229158	High
TCGA-CV-7253	0.989041096	1	0.478061857	High
TCGA-CV-7254	3.997260274	1	0.347224894	Low
TCGA-CV-7255	0.175342466	1	0.368310313	High
TCGA-CV-7263	1.534246575	1	0.416236343	High
TCGA-CV-7406	4.789041096	1	0.37822032	High
TCGA-CV-7407	2.961643836	1	0.418970951	High
TCGA-CV-7411	7.443835616	1	0.319304756	Low
TCGA-CV-7413	0.805479452	1	0.317147783	Low
TCGA-CV-7414	0.038356164	1	0.364014673	High
TCGA-CV-7416	2.090410959	1	0.352113742	Low
TCGA-CV-7423	8.380821918	1	0.385718885	High
TCGA-CV-7425	4.706849315	1	0.458773825	High
TCGA-CV-7427	13.04109589	1	0.434153069	High
TCGA-CV-7428	4.578082192	1	0.463415147	High
TCGA-CV-7429	0.293150685	1	0.419182688	High
TCGA-CV-7432	7.04109589	1	0.519984187	High
TCGA-CV-7434	0.597260274	1	0.4632422	High
TCGA-CV-7435	12.82191781	1	0.352046564	Low
TCGA-CV-7438	0.531506849	1	0.34853714	Low
TCGA-CV-7446	2.994520548	1	0.387266299	High
TCGA-CV-7568	2.539726027	1	0.334911772	Low
TCGA-CV-A45O	2.331506849	0	0.342053144	Low
TCGA-CV-A45P	1.750684932	0	0.408089169	High
TCGA-CV-A45Q	14.11506849	1	0.494471395	High
TCGA-CV-A45R	15.01369863	0	0.294236504	Low
TCGA-CV-A45T	13.30410959	1	0.404339073	High
TCGA-CV-A45U	2.956164384	1	0.416334574	High
TCGA-CV-A45V	0.087671233	1	0.380389969	High
TCGA-CV-A45X	0.542465753	1	0.414022899	High
TCGA-CV-A463	0.063013699	1	0.451587224	High
TCGA-CV-A464	4.717808219	0	0.311799279	Low

TCGA-CV-A465	0.589041096	1	0.439026501	High
TCGA-CV-A468	1.271232877	1	0.334821755	Low
TCGA-CV-A6JD	0.498630137	1	0.299953603	Low
TCGA-CV-A6JE	2.945205479	0	0.435547491	High
TCGA-CV-A6JM	0.531506849	1	0.508433743	High
TCGA-CV-A6JN	2.482191781	0	0.460485517	High
TCGA-CV-A6JO	0.539726027	1	0.422997036	High
TCGA-CV-A6JT	2.334246575	0	0.276199874	Low
TCGA-CV-A6JU	0.301369863	0	0.450512922	High
TCGA-CV-A6JY	1.769863014	0	0.501440721	High
TCGA-CV-A6JZ	1.956164384	0	0.443363398	High
TCGA-CV-A6K0	1.660273973	0	0.314229386	Low
TCGA-CV-A6K2	0.868493151	1	0.386145972	High
TCGA-CX-7085	0.879452055	0	0.237856583	Low
TCGA-CX-7086	1.569863014	0	0.540411834	High
TCGA-CX-7219	2.863013699	0	0.383633597	High
TCGA-CX-A4AQ	4.260273973	0	0.34204191	Low
TCGA-D6-6515	1.104109589	1	0.324327166	Low
TCGA-D6-6516	2.117808219	0	0.474804772	High
TCGA-D6-6823	1.920547945	0	0.56285343	High
TCGA-D6-6825	1.345205479	0	0.40218224	High
TCGA-D6-6827	1.556164384	0	0.453545918	High
TCGA-D6-8569	2.109589041	0	0.422283301	High
TCGA-D6-A4Z9	1.476712329	0	0.447262681	High
TCGA-D6-A4ZB	1.030136986	0	0.352275942	Low
TCGA-D6-A6EM	0.635616438	0	0.496012637	High
TCGA-D6-A6EN	1.202739726	0	0.484336548	High
TCGA-D6-A6EO	1.191780822	0	0.555474027	High
TCGA-DQ-5624	4.871232877	0	0.250624516	Low
TCGA-DQ-5625	3.104109589	1	0.346535931	Low
TCGA-DQ-5630	2.821917808	0	0.261484572	Low
TCGA-DQ-5631	1.501369863	1	0.374493314	High
TCGA-DQ-7588	1.169863014	1	0.549616692	High
TCGA-DQ-7591	1.704109589	0	0.534783802	High
TCGA-DQ-7592	1.41369863	0	0.405889405	High
TCGA-F7-8489	1.802739726	0	0.312803618	Low
TCGA-F7-A50G	1.687671233	0	0.360224952	Low
TCGA-F7-A50J	1.602739726	0	0.38651791	High
TCGA-F7-A61S	1.578082192	0	0.428020999	High
TCGA-F7-A61V	1.052054795	0	0.496116287	High
TCGA-F7-A61W	0.038356164	0	0.469696308	High
TCGA-F7-A620	1.487671233	0	0.443173506	High
TCGA-F7-A624	1.035616438	0	0.541355459	High
TCGA-H7-7774	1.115068493	0	0.443669479	High
TCGA-H7-8501	1.263013699	0	0.46908971	High
TCGA-H7-8502	1.254794521	0	0.45805971	High
TCGA-H7-A6C4	1.134246575	0	0.328006315	Low
TCGA-HD-7831	0.989041096	0	0.152518918	Low
TCGA-HD-7832	0.95890411	0	0.53612597	High
TCGA-HD-8224	1.221917808	1	0.35493226	Low
TCGA-HD-8314	1.835616438	0	0.420550049	High
TCGA-HD-8634	1.054794521	1	0.43507574	High
TCGA-HD-8635	1.904109589	0	0.327592719	Low

TCGA-HD-A4C1	0.030136986	0	0.417721822	High
TCGA-HD-A633	1.153424658	0	0.435164125	High
TCGA-HD-A6HZ	0.304109589	0	0.388511701	High
TCGA-HD-A6I0	0.575342466	0	0.506542676	High
TCGA-HL-7533	2.895890411	0	0.498811897	High
TCGA-IQ-7632	1.208219178	0	0.44505689	High
TCGA-IQ-A61E	3.142465753	0	0.414151203	High
TCGA-IQ-A61G	0.98630137	0	0.550604544	High
TCGA-IQ-A61H	3.117808219	0	0.481638853	High
TCGA-IQ-A61J	2.797260274	0	0.270107311	Low
TCGA-IQ-A6SG	1.58630137	0	0.441715669	High
TCGA-IQ-A6SH	1.290410959	0	0.43908731	High
TCGA-KU-A66T	1.512328767	0	0.410374423	High
TCGA-KU-A6H8	0.895890411	1	0.326606649	Low
TCGA-MT-A51X	0.663013699	0	0.38398785	High
TCGA-MT-A67A	2.504109589	0	0.486699609	High
TCGA-MT-A67D	0.153424658	0	0.435604217	High
TCGA-MT-A67F	1.052054795	0	0.385655837	High
TCGA-MT-A7BN	1.284931507	0	0.412518494	High
TCGA-MZ-A7D7	1.498630137	0	0.464592208	High
TCGA-P3-A5QA	4.728767123	0	0.27620344	Low
TCGA-P3-A5QE	4.271232877	0	0.465158587	High
TCGA-P3-A5QF	0.904109589	1	0.554634817	High
TCGA-P3-A6T0	1.583561644	0	0.327740105	Low
TCGA-P3-A6T2	5.931506849	0	0.46894628	High
TCGA-P3-A6T3	1.580821918	1	0.383698369	High
TCGA-P3-A6T4	0.169863014	1	0.413306488	High
TCGA-P3-A6T5	2.416438356	1	0.375559675	High
TCGA-P3-A6T6	1.082191781	1	0.552952517	High
TCGA-P3-A6T7	1.334246575	1	0.446502133	High
TCGA-P3-A6T8	1.095890411	0	0.24812998	Low
TCGA-QK-A64Z	1.756164384	1	0.381261064	High
TCGA-QK-A652	0.978082192	0	0.323223842	Low
TCGA-QK-A6IG	0.608219178	1	0.499389611	High
TCGA-QK-A6IH	1.391780822	0	0.405229507	High
TCGA-QK-A6II	0.778082192	1	0.398384718	High
TCGA-QK-A6IJ	1.060273973	0	0.460912025	High
TCGA-QK-A6VB	1.232876712	0	0.534844118	High
TCGA-QK-A8Z7	0.542465753	0	0.564456289	High
TCGA-QK-A8Z9	0.964383562	0	0.236174582	Low
TCGA-QK-AA3K	0.693150685	0	0.300398719	Low
TCGA-RS-A6TO	1.060273973	1	0.390030073	High
TCGA-T2-A6WX	0.57260274	1	0.456851846	High
TCGA-T2-A6WZ	1.326027397	1	0.482156612	High
TCGA-T2-A6X2	2.704109589	0	0.373194094	High
TCGA-T3-A92N	0.260273973	1	0.476819246	High
TCGA-UF-A719	4.556164384	0	0.577942162	High
TCGA-UF-A71A	0.235616438	1	0.404750107	High
TCGA-UF-A71B	3.61369863	0	0.435456465	High
TCGA-UF-A71E	3.501369863	0	0.457186554	High
TCGA-UF-A7JA	5.189041096	0	0.362424095	Low
TCGA-UF-A7JC	1.495890411	1	0.427119574	High
TCGA-UF-A7JD	2.01369863	0	0.293161673	Low

TCGA-UF-A7JO	1.728767123	1	0.467419134	High
TCGA-UF-A7JS	1.863013699	1	0.46497219	High
TCGA-UF-A7JT	2.720547945	1	0.435714617	High
TCGA-UP-A6WW	1.419178082	0	0.457719979	High
TCGA-WA-A7GZ	1.712328767	1	0.432352881	High
TCGA-WA-A7H4	1.21369863	0	0.290953168	Low
