# Supplementary material for: Characterization of Cancer Stem Cell Characteristics and Development of a Prognostic Stemness Index Cell-Related Signature in Oral Squamous Cell Carcinoma
Source: Dis Markers. 2021 Nov 9;2021:1571421. doi: 10.1155/2021/1571421 (PMC8617564; doi:10.1155/2021/1571421)
Supplement: Supplementary 3 — Supplementary Table 3: the mRNAsi-related DEGs for OSCC. [file 1571421.f3.pdf]

Supplementary table 3. The mRNAsi-related DEGs for OSCC.

ID	logFC	AveExpr	t	P.Value	adj.P.Val
ACTA1	-2.973267019	3.393800472	-7.932649702	3.61E-14	2.22E-12
DES	-2.893186075	3.847984686	-7.496002617	6.48E-13	3.06E-11
MYL2	-2.517020692	2.468565	-8.184200354	6.53E-15	4.69E-13
MYL1	-2.51139507	2.581848172	-7.912104591	4.15E-14	2.51E-12
CKM	-2.464828111	2.940315849	-7.533850194	5.07E-13	2.46E-11
ACTC1	-2.338005424	2.471317456	-8.123673278	9.88E-15	6.87E-13
MYBPH	-2.335851887	2.460551226	-8.110924558	1.08E-14	7.38E-13
MMP13	-2.278244292	4.063717167	-8.195979338	6.02E-15	4.37E-13
TNNC1	-2.247054734	2.595426186	-7.678132579	1.97E-13	1.03E-11
SLN	-2.216828125	2.297512819	-7.803874507	8.56E-14	4.80E-12
SFRP2	-2.209232695	5.471274248	-11.13840145	1.41E-24	6.60E-22
POSTN	-2.206763232	4.942366099	-10.71500767	4.21E-23	1.50E-20
MB	-2.200712341	2.692784479	-7.802280879	8.65E-14	4.85E-12
COL3A1	-2.157575288	7.946179158	-11.15188386	1.26E-24	5.97E-22
THBS4	-2.14076334	2.200024514	-10.53159285	1.80E-22	5.69E-20
CSRP3	-2.120385015	2.086837148	-7.503783329	6.16E-13	2.93E-11
MYH2	-2.118639832	1.888823102	-7.173409965	5.09E-12	2.03E-10
COL1A2	-2.076557492	7.151146605	-11.50258872	7.23E-26	4.90E-23
FN1	-2.064679905	5.845101882	-9.943526229	1.75E-20	3.74E-18
TNNT3	-2.064385691	3.029286363	-7.091777959	8.50E-12	3.28E-10
COL1A1	-2.051294791	8.213109747	-11.29940845	3.80E-25	2.06E-22
MYLPF	-2.039157375	2.356674816	-7.751149313	1.21E-13	6.61E-12
KLHL41	-2.006218131	1.747704271	-8.475264648	8.65E-16	7.41E-14
TCAP	-1.987500609	2.124539221	-7.391254482	1.27E-12	5.67E-11
TNNI2	-1.978031216	3.067860478	-7.076794119	9.33E-12	3.54E-10
ACTN2	-1.96409005	1.689864847	-8.267665601	3.67E-15	2.77E-13
TNNC2	-1.946931665	2.425149898	-7.333329858	1.85E-12	7.92E-11
ASPN	-1.900298439	3.233258311	-10.80495697	2.05E-23	7.91E-21
MYH7	-1.893619882	1.637578315	-7.821197033	7.62E-14	4.33E-12
COX6A2	-1.82701259	1.676810991	-7.888383987	4.86E-14	2.89E-12
SFRP4	-1.815331845	2.269989888	-9.01737646	1.79E-17	2.10E-15
COL6A3	-1.811637739	4.600568023	-11.20190913	8.40E-25	4.13E-22
MYBPC2	-1.803861687	1.774118806	-7.831195549	7.13E-14	4.07E-12
HSPB7	-1.797346043	1.696874054	-8.324598461	2.48E-15	1.94E-13
TNNI1	-1.787215959	2.098950808	-8.205007236	5.66E-15	4.16E-13
COMP	-1.78106329	2.011573483	-9.960158122	1.54E-20	3.33E-18
COL5A2	-1.774842075	5.128427197	-11.78799564	6.87E-27	6.30E-24
MYH1	-1.751631029	1.524554468	-7.873853345	5.36E-14	3.14E-12
MMP2	-1.751002052	5.72225295	-12.6328315	5.75E-30	1.34E-26
MXRA5	-1.748846277	3.666162745	-12.20934715	2.05E-28	3.25E-25
NRAP	-1.746056296	1.402048769	-7.747207966	1.25E-13	6.77E-12
ANKRD1	-1.74057209	1.818680689	-7.750470167	1.22E-13	6.63E-12
ISLR	-1.735617748	4.090349074	-10.78528791	2.40E-23	9.12E-21
F13A1	-1.729108142	2.547164918	-11.28046183	4.44E-25	2.33E-22
MFAP4	-1.72660681	2.609953703	-11.29621562	3.90E-25	2.09E-22
LRRC15	-1.720514687	2.392335586	-10.57052307	1.32E-22	4.22E-20
CASQ2	-1.70592344	1.666244373	-8.783045596	9.75E-17	9.90E-15
VCAN	-1.697902706	2.795639198	-11.50891929	6.86E-26	4.71E-23
SPARC	-1.681665493	7.637681722	-12.21836661	1.90E-28	3.18E-25
COL5A1	-1.671966355	5.109297558	-11.22650376	6.88E-25	3.48E-22
SMPX	-1.669161481	1.59777848	-7.488568478	6.80E-13	3.18E-11

COL10A1	-1.656823258	2.388447087	-9.502753955	4.97E-19	7.71E-17
MYBPC1	-1.645836933	1.499839274	-7.243712868	3.27E-12	1.35E-10
FLNC	-1.643100497	2.469610121	-8.696166324	1.81E-16	1.76E-14
HSPB6	-1.617544673	1.938769758	-7.925098064	3.80E-14	2.32E-12
COL11A1	-1.613567042	2.069653562	-9.074804164	1.18E-17	1.45E-15
AEBP1	-1.61340136	5.539305243	-10.64392539	7.40E-23	2.51E-20
FBN1	-1.6108728	2.705588774	-11.78150501	7.25E-27	6.36E-24
MMP11	-1.610232392	3.658385306	-8.293489674	3.07E-15	2.36E-13
THBS2	-1.604384355	4.524422677	-9.577685527	2.83E-19	4.63E-17
EEF1A2	-1.588876438	2.759661732	-6.751521044	6.87E-11	2.21E-09
MYOG	-1.575856531	1.45954829	-8.298784756	2.96E-15	2.28E-13
MMP1	-1.574421563	7.429077775	-6.274294108	1.14E-09	2.96E-08
CTSK	-1.569180135	5.069368544	-10.49400336	2.42E-22	7.12E-20
MYOZ1	-1.566437339	1.491762352	-7.508471148	5.97E-13	2.85E-11
PDLIM3	-1.561843886	1.958395214	-9.18199821	5.38E-18	6.91E-16
HSPB3	-1.557509587	2.150766022	-8.864859848	5.41E-17	5.75E-15
TIMP2	-1.554793631	4.753330858	-12.56045946	1.06E-29	2.27E-26
STAC3	-1.553306122	2.391337743	-7.509569315	5.93E-13	2.83E-11
FBLN2	-1.552290859	4.048513748	-10.67502609	5.78E-23	2.02E-20
CTHRC1	-1.535508901	4.207125828	-9.956915938	1.58E-20	3.40E-18
SULF1	-1.533355705	3.213660082	-10.11399541	4.71E-21	1.11E-18
EMILIN1	-1.520607934	3.321047378	-10.85714538	1.35E-23	5.29E-21
ANGPTL2	-1.50320042	3.755809216	-12.302221	9.39E-29	1.62E-25
ADAMTS2	-1.492097971	3.197004826	-11.29421869	3.97E-25	2.10E-22
NEB	-1.491208139	1.209562436	-8.752695549	1.21E-16	1.21E-14
PCOLCE	-1.473741457	3.755306345	-11.16100118	1.17E-24	5.60E-22
FHL1	-1.470432011	3.030938064	-7.652917929	2.32E-13	1.20E-11
CRYAB	-1.468467805	4.349506457	-7.53750098	4.95E-13	2.41E-11
COL6A2	-1.468386462	6.772201156	-10.26938588	1.41E-21	3.57E-19
COL6A1	-1.464317992	6.565912145	-10.28070417	1.29E-21	3.29E-19
FNDC1	-1.460435601	1.931191658	-9.833176137	4.08E-20	7.97E-18
FIBIN	-1.456942695	1.535156285	-14.07042499	2.37E-35	3.17E-31
CHRNA1	-1.450388021	1.335901066	-9.088131377	1.07E-17	1.33E-15
LUM	-1.449073064	6.142560535	-9.029117449	1.65E-17	1.95E-15
COL8A1	-1.447901181	1.803404158	-12.75920651	1.96E-30	5.25E-27
LMOD2	-1.445228432	1.134889188	-8.095855046	1.19E-14	8.11E-13
CCDC80	-1.441059766	2.389271551	-12.09909301	5.16E-28	7.20E-25
MYOT	-1.435754046	1.260514498	-8.138447997	8.93E-15	6.26E-13
MGP	-1.433497227	3.182115545	-9.26504245	2.91E-18	3.91E-16
APOBEC2	-1.432778725	1.240379722	-8.070847982	1.42E-14	9.47E-13
OLFML2B	-1.429090363	3.35521466	-11.36780087	2.18E-25	1.28E-22
APOD	-1.4222237	2.257732648	-8.589195565	3.88E-16	3.53E-14
TAGLN	-1.419719224	4.412943697	-9.99158428	1.21E-20	2.65E-18
BGN	-1.417930039	7.216539068	-9.676447449	1.34E-19	2.37E-17
CCN2	-1.417459057	5.370194772	-9.088427522	1.07E-17	1.33E-15
DCN	-1.414991493	3.928840907	-9.92795519	1.98E-20	4.15E-18
DPT	-1.412911138	2.282874024	-9.625759705	1.97E-19	3.35E-17
ADAM12	-1.412270722	2.317096285	-11.48777392	8.16E-26	5.46E-23
IGFBP5	-1.398090226	4.307415869	-7.910194405	4.20E-14	2.53E-12
THY1	-1.357669067	3.614852289	-10.45459595	3.30E-22	9.55E-20
MMP10	-1.352968663	5.080106225	-4.787890597	2.58E-06	3.49E-05
SERPINF1	-1.352821546	4.601147172	-9.183710416	5.31E-18	6.85E-16
MMP7	-1.348831839	3.101649941	-6.313535163	9.08E-10	2.40E-08

IGF2	-1.342981065	3.001711897	-9.607724955	2.26E-19	3.77E-17
MXRA8	-1.342586362	3.493528061	-11.18729963	9.46E-25	4.60E-22
SYNPO2	-1.342155184	1.654205798	-7.559284859	4.29E-13	2.12E-11
TPM2	-1.338290076	4.94349412	-7.729793163	1.40E-13	7.48E-12
CHI3L1	-1.33594371	3.355695139	-6.657703214	1.21E-10	3.73E-09
OLFML3	-1.334016497	3.132984546	-10.95575915	6.13E-24	2.61E-21
MYF6	-1.334004219	1.17369794	-7.733060836	1.37E-13	7.35E-12
SPOCK1	-1.329739252	1.876864799	-10.29098116	1.19E-21	3.06E-19
XIRP2	-1.327736759	0.967792948	-7.451460979	8.64E-13	3.96E-11
FMOD	-1.323062819	3.756115607	-10.80212973	2.10E-23	8.03E-21
PDGFRB	-1.322535669	3.758613259	-10.69258208	5.03E-23	1.78E-20
CHGB	-1.314688443	1.458959772	-6.527560662	2.61E-10	7.61E-09
FSTL1	-1.311222643	4.57693567	-10.68408626	5.38E-23	1.89E-20
MYL9	-1.309970958	4.748074423	-9.513634861	4.58E-19	7.14E-17
MYH3	-1.305122004	1.452780416	-8.288845915	3.17E-15	2.42E-13
MMP3	-1.301016041	4.887578896	-5.21826607	3.25E-07	5.32E-06
CAVIN4	-1.298001234	1.257839725	-9.014419986	1.83E-17	2.14E-15
DIO2	-1.297434948	2.55487389	-9.6285604	1.93E-19	3.30E-17
HTRA3	-1.296576412	3.815510178	-8.844656691	6.26E-17	6.56E-15
CXCL12	-1.289704097	2.123809868	-9.92028547	2.10E-20	4.35E-18
PODN	-1.289087762	1.320166401	-11.98097592	1.38E-27	1.51E-24
AC245297.1	-1.288855775	1.567387635	-8.83454558	6.73E-17	7.01E-15
SPON1	-1.28843853	1.853320257	-11.12909605	1.52E-24	6.99E-22
RARRES2	-1.286534682	3.184143992	-8.400181889	1.46E-15	1.20E-13
PRRX1	-1.27799423	2.933872413	-9.873541585	3.00E-20	6.01E-18
AMTN	-1.273492174	2.804528297	-4.36153523	1.74E-05	0.000195737
FAP	-1.270127427	1.991707668	-11.99851527	1.20E-27	1.36E-24
RCN3	-1.263244265	3.877106854	-8.765933205	1.10E-16	1.11E-14
CMYA5	-1.263109618	1.105639114	-8.063436553	1.49E-14	9.88E-13
GASK1B	-1.257487287	2.096103426	-13.29784831	1.94E-32	1.04E-28
TGFB3	-1.256864956	2.845354117	-11.60615911	3.09E-26	2.23E-23
HRC	-1.246534499	1.099575412	-8.11521076	1.05E-14	7.21E-13
CPXM1	-1.240638265	2.906608143	-9.618482994	2.08E-19	3.52E-17
ENO3	-1.236427623	1.554551	-7.322801369	1.98E-12	8.41E-11
ANTXR1	-1.230700506	3.623879857	-11.11967056	1.64E-24	7.36E-22
CST1	-1.226527641	2.082174125	-5.218790048	3.24E-07	5.31E-06
TRDN	-1.223272033	0.937264995	-8.118530406	1.02E-14	7.08E-13
GREM1	-1.213868497	2.060631301	-9.91198438	2.23E-20	4.60E-18
ACTA2	-1.213588862	4.342228153	-9.071776971	1.21E-17	1.47E-15
MEF2C	-1.211951186	1.395532729	-11.08617635	2.14E-24	9.57E-22
LRRC17	-1.210195362	1.264265607	-10.5976953	1.07E-22	3.54E-20
NNMT	-1.208353501	4.45309744	-7.458278607	8.27E-13	3.81E-11
PDGFRL	-1.206098524	1.534288036	-10.7159159	4.18E-23	1.50E-20
LAMP5	-1.19919013	1.756605175	-10.90604892	9.14E-24	3.71E-21
FILIP1L	-1.199029693	2.330863819	-12.91502804	5.20E-31	1.74E-27
PADI2	-1.197586762	1.567191038	-7.625655563	2.78E-13	1.41E-11
CA3	-1.193000502	0.989090827	-8.127833769	9.60E-15	6.69E-13
PPP1R27	-1.190844385	1.217164822	-7.052693137	1.08E-11	4.04E-10
COL12A1	-1.19001222	4.575352651	-8.023004587	1.96E-14	1.27E-12
RCAN2	-1.180525441	1.450827871	-14.85654692	2.36E-38	6.32E-34
PRELP	-1.179978169	1.372031142	-9.201555601	4.66E-18	6.05E-16
TNS1	-1.178737543	2.306966353	-11.78668227	6.95E-27	6.30E-24
ELN	-1.178413749	1.646875531	-8.23072512	4.74E-15	3.52E-13

FABP3	-1.17539451	2.253255162	-7.467876284	7.77E-13	3.60E-11
TMEM119	-1.172965221	1.828038531	-9.482369547	5.79E-19	8.82E-17
ADAMTS12	-1.170145958	1.587050451	-11.31408099	3.38E-25	1.84E-22
SDC2	-1.167687136	2.741875683	-9.442233021	7.81E-19	1.16E-16
COL14A1	-1.165380067	1.725068363	-10.52705439	1.87E-22	5.84E-20
DACT1	-1.163428684	1.252497926	-12.65809509	4.64E-30	1.18E-26
A2M	-1.16056185	4.678329516	-9.63253522	1.87E-19	3.22E-17
HSPG2	-1.15882795	3.806957841	-8.54031983	5.47E-16	4.81E-14
MYL3	-1.156676247	0.898450489	-7.411041331	1.12E-12	5.02E-11
NID1	-1.155856192	3.718863618	-7.954726197	3.11E-14	1.93E-12
SCT	-1.154352706	1.318941523	-9.757304191	7.27E-20	1.37E-17
ATP1A2	-1.147754302	0.841064442	-8.475850028	8.61E-16	7.39E-14
CDH11	-1.144863913	1.972122122	-9.513994576	4.57E-19	7.14E-17
TUBA1A	-1.144703743	4.367757839	-10.52191594	1.94E-22	6.02E-20
LOXL1	-1.139868113	2.655379594	-9.877147731	2.92E-20	5.87E-18
SGCD	-1.139279886	0.874680034	-13.98083868	5.19E-35	5.55E-31
DDIT4L	-1.136675682	0.991247405	-8.445256299	1.07E-15	8.96E-14
CACNG1	-1.136431227	0.994215977	-7.927143045	3.75E-14	2.29E-12
CRISPLD2	-1.134969256	2.708030141	-11.17330599	1.06E-24	5.11E-22
S100A1	-1.127784503	1.388610252	-7.888592093	4.86E-14	2.89E-12
GPC4	-1.127366366	1.891282349	-7.935343365	3.55E-14	2.18E-12
EFEMP2	-1.124852865	2.363707727	-11.21478719	7.57E-25	3.75E-22
SGCA	-1.119605216	0.946125551	-8.927337918	3.45E-17	3.78E-15
ASB5	-1.114301154	0.872095772	-8.283213154	3.30E-15	2.51E-13
TNFAIP6	-1.112602384	2.475809273	-9.181900187	5.38E-18	6.91E-16
FBLN5	-1.108562058	1.957515237	-11.64410375	2.26E-26	1.66E-23
PXDN	-1.1076373	3.142734991	-8.167186045	7.34E-15	5.22E-13
LOXL2	-1.105758148	3.595043723	-9.022589116	1.73E-17	2.03E-15
COL8A2	-1.104913692	2.653263727	-9.450866445	7.32E-19	1.10E-16
ANXA6	-1.103898757	3.541104318	-9.751430677	7.60E-20	1.43E-17
ATP2A1	-1.103822679	1.091952817	-6.285213783	1.07E-09	2.80E-08
OMD	-1.103427215	1.032257732	-10.12043949	4.48E-21	1.06E-18
DAB2	-1.100322838	2.240302974	-12.11660043	4.46E-28	6.45E-25
CHRND	-1.100084603	0.83282902	-8.307074809	2.80E-15	2.16E-13
NES	-1.098870546	3.405148121	-7.680825868	1.93E-13	1.01E-11
PLPP4	-1.09677838	1.377844571	-9.926609439	2.00E-20	4.17E-18
PTGDS	-1.096559153	2.033275018	-8.323508835	2.49E-15	1.95E-13
MFAP5	-1.09388502	2.515614604	-6.250595649	1.30E-09	3.35E-08
CASQ1	-1.091882163	0.832478534	-6.835204076	4.14E-11	1.39E-09
DUSP26	-1.090266169	0.867404184	-8.160370841	7.69E-15	5.42E-13
LINC01614	-1.088504621	1.175865284	-9.010970481	1.88E-17	2.19E-15
DIRAS1	-1.088124953	1.036288355	-11.13573209	1.44E-24	6.69E-22
ADGRA2	-1.086180852	1.818725554	-11.72524915	1.16E-26	9.37E-24
GLT8D2	-1.085830547	1.794240042	-12.61241737	6.83E-30	1.52E-26
XIRP1	-1.085617852	1.076732242	-8.006704173	2.19E-14	1.41E-12
LRRC32	-1.083320127	2.389718862	-12.32537404	7.73E-29	1.43E-25
LMOD3	-1.083261703	0.836803712	-8.042034843	1.72E-14	1.13E-12
EDIL3	-1.080760626	2.088280214	-7.979855934	2.63E-14	1.66E-12
PMEPA1	-1.077585348	4.506108658	-8.731802255	1.41E-16	1.40E-14
ITGA7	-1.073931542	1.66783123	-9.24675712	3.34E-18	4.42E-16
ITGA11	-1.071941691	1.22820537	-10.35132564	7.43E-22	2.01E-19
LTBP2	-1.067840419	3.880383637	-9.005475714	1.96E-17	2.26E-15
MYOZ2	-1.066929048	0.820199744	-7.424929264	1.03E-12	4.63E-11

COLEC12	-1.065448349	1.350767039	-11.88361911	3.11E-27	3.03E-24
SHISA4	-1.065202263	2.220503182	-9.331174859	1.79E-18	2.49E-16
COL5A3	-1.061766536	3.583597212	-8.990416277	2.18E-17	2.49E-15
TNNT1	-1.061606902	4.408801672	-4.858602314	1.85E-06	2.59E-05
CREB3L1	-1.061142524	2.078908956	-8.320826264	2.54E-15	1.99E-13
CD248	-1.058756495	3.976596307	-7.610694196	3.07E-13	1.55E-11
AC080038.1	-1.05706177	4.100966991	-9.602498314	2.35E-19	3.89E-17
PMP22	-1.056944982	3.743662151	-10.26550414	1.45E-21	3.67E-19
SPARCL1	-1.055449534	3.983566786	-8.067008487	1.45E-14	9.69E-13
COX7A1	-1.050955605	2.452310593	-7.341954867	1.75E-12	7.54E-11
LIMCH1	-1.050409239	1.543608003	-10.32447351	9.16E-22	2.38E-19
LOX	-1.049838125	2.958795159	-7.323391359	1.97E-12	8.39E-11
COL15A1	-1.047099362	2.857585313	-8.277702968	3.43E-15	2.61E-13
TRIM54	-1.042960913	1.058643326	-7.114834299	7.36E-12	2.86E-10
NID2	-1.042559116	1.639374831	-9.954515391	1.61E-20	3.45E-18
DDR2	-1.039348136	1.340689347	-12.84334123	9.58E-31	2.85E-27
CPQ	-1.037851696	2.244858653	-11.42604852	1.35E-25	8.34E-23
ZCCHC24	-1.031835091	2.007205026	-14.87869452	1.94E-38	6.32E-34
EPDR1	-1.030357463	1.325247139	-10.94078142	6.92E-24	2.87E-21
SPON2	-1.028979777	3.638119209	-8.797944977	8.76E-17	9.00E-15
MEDAG	-1.027210952	1.978258126	-8.784334547	9.66E-17	9.83E-15
LDB3	-1.022530658	0.81871875	-7.556748111	4.36E-13	2.15E-11
CCN4	-1.021578487	1.911203577	-9.161963873	6.23E-18	7.86E-16
HTRA1	-1.021419039	6.086871884	-8.575885935	4.26E-16	3.85E-14
TPM1	-1.016403254	3.482040681	-9.034592504	1.58E-17	1.89E-15
MAP1A	-1.015400767	1.27413876	-11.44102428	1.20E-25	7.63E-23
LAMA4	-1.014578536	2.511557488	-10.34238115	7.97E-22	2.13E-19
CPE	-1.013999874	3.397798292	-7.362546396	1.53E-12	6.71E-11
NMRK2	-1.013095044	0.862330811	-7.116604909	7.28E-12	2.84E-10
LHFPL6	-1.008852779	3.245862537	-11.34701866	2.58E-25	1.47E-22
CERCAM	-1.008785073	3.518610099	-9.600475668	2.38E-19	3.94E-17
MSC	-1.007106879	3.030998831	-7.492062385	6.65E-13	3.12E-11
F2RL2	-1.006234565	2.004550656	-8.191356234	6.21E-15	4.49E-13
RGS5	-1.005245977	2.147586172	-8.953554219	2.85E-17	3.15E-15
MSRB3	-1.004039191	1.669239953	-11.78719048	6.92E-27	6.30E-24
CNN1	-1.002292496	1.598271817	-8.940531334	3.13E-17	3.45E-15
ZNF469	-1.000970803	1.230538733	-11.77839851	7.44E-27	6.43E-24
DPYSL3	-1.00074798	3.459408795	-6.78001496	5.78E-11	1.89E-09
F2R	-0.997554188	3.44945297	-9.46859852	6.41E-19	9.70E-17
PRUNE2	-0.996161932	0.747540607	-10.22643884	1.97E-21	4.88E-19
MYOM3	-0.996029578	1.48275298	-8.375926493	1.73E-15	1.39E-13
MMP9	-0.995617735	5.502670123	-5.317150757	1.98E-07	3.40E-06
LAMA2	-0.994124063	1.142846151	-11.66538373	1.89E-26	1.43E-23
VIM	-0.99312666	6.473265924	-8.25232697	4.08E-15	3.07E-13
CCL19	-0.988505463	2.577817681	-4.881019993	1.67E-06	2.35E-05
SOD3	-0.983653745	2.195279432	-9.861308382	3.29E-20	6.55E-18
HSPB8	-0.978335456	4.347206734	-5.921334334	8.22E-09	1.83E-07
MRAS	-0.978290911	1.510702467	-12.65300903	4.84E-30	1.18E-26
LYZ	-0.978030537	5.229908556	-4.483746599	1.02E-05	0.000121123
SRPX	-0.977953341	2.627701753	-6.840090464	4.02E-11	1.36E-09
ADRA2A	-0.977592669	0.895147073	-11.12458765	1.57E-24	7.13E-22
SLC24A3	-0.972175455	2.462549667	-8.283417615	3.29E-15	2.51E-13
SGCG	-0.971623447	0.808561312	-8.251129437	4.12E-15	3.09E-13

CCN1	-0.971315902	5.129459387	-7.435969004	9.55E-13	4.34E-11
SCARF2	-0.971015817	1.653061569	-10.48016324	2.70E-22	7.85E-20
FKBP10	-0.969414333	3.884711006	-6.023411517	4.68E-09	1.09E-07
INHBA	-0.967650666	3.847344717	-5.753174515	2.05E-08	4.22E-07
PLA2G2A	-0.966440694	1.349492293	-4.86022725	1.84E-06	2.57E-05
HLA-DQB2	-0.96581933	3.114471363	-5.513300199	7.25E-08	1.35E-06
TTN	-0.965646713	0.695357875	-7.864669594	5.70E-14	3.32E-12
CCL18	-0.963985549	3.542895955	-4.771487567	2.78E-06	3.74E-05
CLEC11A	-0.963915905	2.9540601	-8.185965161	6.45E-15	4.66E-13
C3	-0.963450621	3.874137781	-5.548569547	6.04E-08	1.15E-06
FBXO32	-0.960821132	3.040198609	-7.779134501	1.01E-13	5.58E-12
TNS3	-0.957489526	2.664849801	-10.19963706	2.43E-21	5.93E-19
IGFBP4	-0.95614803	6.726975944	-7.644606318	2.45E-13	1.26E-11
CILP	-0.954946608	0.611581141	-10.48760901	2.55E-22	7.45E-20
GGT5	-0.954368146	2.399752511	-10.05070118	7.69E-21	1.74E-18
MYADM	-0.952755411	4.746893184	-7.078699066	9.22E-12	3.50E-10
RBM24	-0.952599756	0.772309508	-9.391907735	1.14E-18	1.64E-16
CORO6	-0.952323956	1.654104637	-6.582615727	1.89E-10	5.62E-09
AQP1	-0.952079367	4.070584068	-8.986725036	2.24E-17	2.55E-15
GEM	-0.949344632	2.56935367	-7.767156237	1.09E-13	6.00E-12
CCL2	-0.945431795	3.011843425	-6.793572972	5.33E-11	1.76E-09
PDZRN3	-0.945055738	1.311460978	-11.35018941	2.51E-25	1.45E-22
DYSF	-0.943888054	2.131538437	-9.729090361	9.01E-20	1.67E-17
CKMT2	-0.943306319	0.845120809	-6.996647352	1.54E-11	5.56E-10
TMEM176B	-0.94286505	3.875789097	-6.632408815	1.40E-10	4.28E-09
UNC45B	-0.941644818	0.650921326	-9.072552828	1.20E-17	1.47E-15
BICC1	-0.939568201	1.207219766	-12.20858202	2.06E-28	3.25E-25
NDN	-0.934847874	2.681339221	-6.439111571	4.39E-10	1.23E-08
NEXN	-0.933272727	1.939768686	-7.342827871	1.74E-12	7.51E-11
FCER1A	-0.932184958	1.435696075	-7.519504424	5.56E-13	2.67E-11
SERPING1	-0.931781838	5.436652272	-6.370482211	6.54E-10	1.78E-08
SORCS2	-0.931661382	1.961311488	-8.103872878	1.13E-14	7.70E-13
PYGM	-0.929040748	0.720299078	-6.221095195	1.54E-09	3.92E-08
SYNPO2L	-0.927238711	0.919220149	-7.656710179	2.27E-13	1.17E-11
TNC	-0.925453857	6.128528276	-4.910793321	1.45E-06	2.07E-05
AGT	-0.923762206	0.991984001	-8.818626861	7.55E-17	7.82E-15
FOLR2	-0.923285984	2.228480899	-7.185567188	4.72E-12	1.89E-10
TIMP3	-0.923207863	1.239042965	-10.16625848	3.14E-21	7.51E-19
CACNA1S	-0.922470367	0.616375743	-8.388463669	1.59E-15	1.28E-13
CD207	-0.91846413	1.868616062	-6.127727556	2.61E-09	6.35E-08
MYOM1	-0.918359351	0.787267726	-7.564510171	4.15E-13	2.05E-11
COL18A1	-0.916035871	4.552647047	-9.263671721	2.94E-18	3.94E-16
CALD1	-0.915465021	4.410883256	-9.479907426	5.89E-19	8.96E-17
HMCN1	-0.914762067	0.931351883	-12.006414	1.12E-27	1.30E-24
TMEM47	-0.913722529	1.399337504	-11.98681176	1.32E-27	1.47E-24
PDK4	-0.912443823	1.194015359	-6.440669995	4.35E-10	1.22E-08
PDGFRA	-0.912387758	1.528388906	-8.979128527	2.37E-17	2.66E-15
RGS16	-0.912256852	2.173344809	-8.336475336	2.28E-15	1.80E-13
IGFBP7	-0.912227962	6.503235584	-9.763659736	6.93E-20	1.31E-17
HEYL	-0.908686848	1.838376701	-10.73793219	3.50E-23	1.28E-20
FZD1	-0.907241864	2.583043299	-10.24774423	1.67E-21	4.17E-19
CSPG4	-0.906314054	3.909869064	-5.492927132	8.06E-08	1.49E-06
CPXM2	-0.904532318	1.720625477	-8.104402477	1.13E-14	7.70E-13

PKIA	-0.903741435	1.293357434	-7.417685376	1.07E-12	4.83E-11
LAMB2	-0.903310809	3.982110097	-10.87908694	1.13E-23	4.50E-21
HEG1	-0.901652223	2.968164129	-9.18633772	5.21E-18	6.73E-16
P3H3	-0.897237456	2.268132897	-7.52992063	5.20E-13	2.52E-11
DEPP1	-0.895357709	3.233391934	-6.735601872	7.56E-11	2.42E-09
SMYD1	-0.895108957	0.624824961	-8.075952444	1.37E-14	9.21E-13
CYP27A1	-0.894639663	1.92969591	-6.443628292	4.27E-10	1.21E-08
TRIM63	-0.893986679	0.897694487	-6.204320634	1.69E-09	4.27E-08
PLN	-0.893436785	0.75514086	-9.105952408	9.39E-18	1.17E-15
SRL	-0.891826163	0.847549945	-6.883706733	3.08E-11	1.06E-09
MYPN	-0.891804459	0.649974061	-8.066852078	1.46E-14	9.69E-13
DUSP27	-0.891633873	0.635953053	-8.380085605	1.68E-15	1.36E-13
JAM3	-0.89125898	1.64673763	-11.68238802	1.65E-26	1.26E-23
ZEB1	-0.891080969	1.365423167	-13.06931012	1.39E-31	5.71E-28
SLC40A1	-0.890574326	2.872007512	-6.795715536	5.26E-11	1.74E-09
PLXDC2	-0.888507264	2.403505596	-8.896924377	4.29E-17	4.63E-15
WNT11	-0.888388048	1.214377376	-6.910530158	2.61E-11	9.13E-10
APLNR	-0.88781792	1.902016162	-8.054241279	1.59E-14	1.04E-12
SSPN	-0.886420342	1.487460175	-11.32584662	3.07E-25	1.70E-22
SSC5D	-0.886170244	0.985665452	-11.7176996	1.23E-26	9.82E-24
C11orf96	-0.884082522	2.222554529	-7.330654746	1.88E-12	8.02E-11
BHLHE41	-0.883950062	1.94757302	-7.74234104	1.29E-13	6.96E-12
SERPINE1	-0.882942473	6.36464232	-4.835346201	2.07E-06	2.86E-05
SCN1B	-0.88146801	1.137928727	-9.60322763	2.33E-19	3.88E-17
THBS1	-0.880660318	4.68888342	-5.033953647	8.03E-07	1.21E-05
LMCD1	-0.880387269	1.513572084	-11.23137207	6.62E-25	3.37E-22
OLFML1	-0.880332879	1.193465976	-12.04524686	8.09E-28	1.01E-24
AXL	-0.879882591	3.229122939	-7.004065373	1.47E-11	5.33E-10
NCAM1	-0.879772526	0.771652539	-9.362256869	1.42E-18	2.01E-16
AC093010.3	-0.878143817	1.712777006	-11.69107071	1.53E-26	1.19E-23
KLHL40	-0.87693266	0.673756672	-7.219401173	3.81E-12	1.55E-10
CSF1R	-0.876613873	2.989809283	-6.992390844	1.58E-11	5.69E-10
ALPK3	-0.876464278	0.96031676	-9.592065235	2.54E-19	4.18E-17
LRP1	-0.875850031	4.029226916	-8.839125502	6.51E-17	6.80E-15
FBLN1	-0.873445877	4.182477699	-6.74103538	7.32E-11	2.34E-09
GXYLT2	-0.873143867	1.326883575	-10.02673767	9.25E-21	2.04E-18
RGS4	-0.870775576	1.365427184	-8.395210565	1.51E-15	1.23E-13
IL1R1	-0.868815469	3.069576374	-9.175705549	5.63E-18	7.19E-16
GPC6	-0.867184322	1.045442693	-9.313599368	2.03E-18	2.81E-16
PLPP7	-0.866520966	0.965270189	-10.59756949	1.07E-22	3.54E-20
ZNF521	-0.86641377	0.91058737	-13.22384256	3.68E-32	1.65E-28
AKAP12	-0.865142554	1.309723601	-9.083182816	1.11E-17	1.37E-15
GPX7	-0.862021761	2.263960432	-7.59122768	3.48E-13	1.74E-11
TMEM204	-0.861189065	2.69456256	-9.440418216	7.92E-19	1.17E-16
EGR2	-0.860492352	1.915805287	-6.824015692	4.43E-11	1.49E-09
TXLNB	-0.859205129	0.71136391	-8.569597448	4.45E-16	4.00E-14
PPP1R3C	-0.858671161	1.712377522	-5.945206963	7.21E-09	1.62E-07
MAN1A1	-0.858363577	3.182764856	-7.697096067	1.74E-13	9.14E-12
RETREG1	-0.85675421	1.274648345	-9.6043752	2.31E-19	3.86E-17
KIF26B	-0.855337612	1.352938641	-12.09711307	5.25E-28	7.20E-25
P4HA3	-0.855146487	0.935176426	-12.14026909	3.66E-28	5.44E-25
GAS1	-0.852300326	2.890246217	-7.860890696	5.85E-14	3.40E-12
RUSC2	-0.850855493	2.041532868	-11.03858772	3.15E-24	1.38E-21

SLCO2A1	-0.84947059	2.555427558	-6.79415735	5.31E-11	1.75E-09
SMOC2	-0.848272541	1.421272095	-8.022688718	1.97E-14	1.27E-12
PKIG	-0.847370466	3.066911997	-9.82478957	4.35E-20	8.44E-18
PRSS23	-0.847225441	3.451717274	-7.148498129	5.96E-12	2.35E-10
C10orf71	-0.847118374	0.540094322	-8.104225136	1.13E-14	7.70E-13
SEMA3C	-0.846957307	3.732229554	-6.316979185	8.90E-10	2.36E-08
NPR2	-0.845076891	1.299674567	-8.885409614	4.67E-17	5.02E-15
IGLV2-14	-0.844254949	4.434208419	-2.700748044	0.007285856	0.037913767
CTSF	-0.843985505	2.934791287	-7.162173429	5.47E-12	2.17E-10
FERMT2	-0.843935029	1.574870086	-10.07782163	6.23E-21	1.41E-18
APOE	-0.843748771	5.234657455	-4.391922264	1.53E-05	0.000174256
ADAM19	-0.842350616	2.313455125	-7.078636394	9.23E-12	3.50E-10
ARMCX1	-0.842239524	1.973250243	-8.083143678	1.30E-14	8.82E-13
C1QTNF3	-0.842230873	0.750562109	-9.85386046	3.48E-20	6.91E-18
ECM2	-0.842027432	1.157390879	-12.04484844	8.12E-28	1.01E-24
RAI2	-0.840748779	1.014280309	-11.06060864	2.64E-24	1.17E-21
TWIST1	-0.839910085	2.348837336	-8.953585724	2.85E-17	3.15E-15
KANK4	-0.838828573	0.936525802	-8.802522678	8.47E-17	8.72E-15
TMEM176A	-0.83783563	2.407361912	-7.482640789	7.06E-13	3.30E-11
ZBTB47	-0.83735211	2.06348319	-12.02326373	9.72E-28	1.18E-24
HLA-DQA2	-0.836729563	3.152189511	-4.21340858	3.27E-05	0.000343965
TBX15	-0.836204503	1.085174512	-9.226283419	3.88E-18	5.10E-16
IP6K3	-0.835737575	0.741429032	-7.537783027	4.94E-13	2.41E-11
ARMCX2	-0.835190114	1.518064017	-8.182186893	6.62E-15	4.75E-13
ITGA5	-0.834859303	4.540811394	-6.338099981	7.88E-10	2.11E-08
MAMDC2	-0.834008322	0.967431371	-9.391706484	1.14E-18	1.64E-16
COL4A1	-0.833032968	5.497314909	-6.700456853	9.34E-11	2.93E-09
CNN3	-0.832534855	4.967832895	-8.713240868	1.61E-16	1.58E-14
HEPH	-0.831095409	1.151586871	-11.32533201	3.08E-25	1.70E-22
EFEMP1	-0.83060787	3.935364904	-6.163725769	2.13E-09	5.28E-08
CXCL14	-0.829745959	7.464443441	-3.205944932	0.001481816	0.00985778
ITGB2	-0.829740304	3.18554353	-6.070979959	3.59E-09	8.48E-08
COL16A1	-0.829632052	3.8581359	-7.850989565	6.25E-14	3.62E-12
DCHS1	-0.827159462	1.075768753	-11.21660003	7.46E-25	3.73E-22
PLPP3	-0.824775041	2.498762634	-7.608134897	3.12E-13	1.57E-11
CYS1	-0.824119571	0.956781803	-12.30561241	9.13E-29	1.62E-25
FGFR1	-0.823652015	1.681627833	-9.028961651	1.65E-17	1.95E-15
BMERB1	-0.82359854	1.260390482	-14.42500205	1.06E-36	1.89E-32
SYNPO	-0.823478553	3.834514791	-8.119591712	1.02E-14	7.05E-13
TGFBI	-0.823310595	6.447269361	-4.747571971	3.11E-06	4.13E-05
CYBRD1	-0.823310488	3.37334321	-6.558213803	2.18E-10	6.42E-09
MYO18B	-0.823088708	0.628194822	-8.558550506	4.81E-16	4.29E-14
CCL21	-0.822229408	3.81615169	-3.560368198	0.000426403	0.003330463
ARHGEF40	-0.820662385	1.908021211	-9.847225826	3.67E-20	7.24E-18
NREP	-0.81980378	2.544889868	-9.174382157	5.69E-18	7.25E-16
GUCY1A1	-0.818856323	1.537403671	-8.763080543	1.12E-16	1.13E-14
MYL4	-0.818612811	0.7100678	-7.473273751	7.51E-13	3.49E-11
DDN	-0.817522315	1.151964226	-5.654964496	3.45E-08	6.82E-07
CAV3	-0.815591895	0.636610212	-7.99194947	2.42E-14	1.54E-12
TSPAN7	-0.814843986	2.317061367	-4.98679477	1.01E-06	1.48E-05
CCL22	-0.814610499	2.0209022	-7.477365865	7.31E-13	3.41E-11
CH25H	-0.810306917	1.613817198	-7.205281317	4.17E-12	1.69E-10
CD93	-0.809964181	2.75259709	-7.785931075	9.64E-14	5.35E-12

PLXDC1	-0.809852308	1.459529735	-10.97380973	5.31E-24	2.27E-21
MFAP2	-0.809828166	4.231315599	-6.771960384	6.07E-11	1.97E-09
VCAM1	-0.809336896	1.689148805	-5.426692607	1.13E-07	2.05E-06
JCAD	-0.809317733	1.31622687	-11.35661114	2.39E-25	1.39E-22
BASP1	-0.809106107	5.010405174	-5.698857205	2.73E-08	5.52E-07
AC068506.1	-0.808860623	0.58373842	-7.126461133	6.84E-12	2.68E-10
C1QTNF1	-0.807297849	3.148788109	-7.879710839	5.15E-14	3.05E-12
SULF2	-0.805748603	5.401118638	-6.169599883	2.06E-09	5.12E-08
EDNRA	-0.805563657	2.422496283	-7.786662708	9.59E-14	5.34E-12
C1S	-0.804590078	5.716144669	-6.8673545	3.40E-11	1.16E-09
HJV	-0.804504229	0.578747398	-7.346232588	1.70E-12	7.37E-11
AC104083.1	-0.803953212	1.601315439	-9.421774736	9.10E-19	1.33E-16
CMTM3	-0.803408464	3.283954259	-9.202411685	4.63E-18	6.02E-16
MAGEH1	-0.803198268	2.609791539	-8.83905424	6.52E-17	6.80E-15
SLC16A2	-0.800528822	2.377776754	-6.77373683	6.01E-11	1.96E-09
LCP1	-0.798902469	4.22372531	-5.461222698	9.49E-08	1.74E-06
WNT5B	-0.798165983	1.646265003	-7.106920235	7.73E-12	3.00E-10
SYNDIG1	-0.797591661	0.725708958	-9.408222317	1.01E-18	1.47E-16
RASA3	-0.795546852	2.243469858	-9.061018921	1.30E-17	1.58E-15
TMOD1	-0.794856089	0.916571019	-7.026562006	1.28E-11	4.69E-10
ITGAV	-0.794800694	4.591788745	-6.680722362	1.05E-10	3.28E-09
SH3BGRL	-0.794713861	4.493587664	-7.872212767	5.42E-14	3.17E-12
OLFM2	-0.794090136	3.313254021	-6.022130021	4.71E-09	1.09E-07
CAP2	-0.793647465	1.881270003	-8.338764441	2.24E-15	1.78E-13
SRPX2	-0.793609863	2.808282222	-7.910617085	4.19E-14	2.52E-12
CYBB	-0.791886515	2.688996397	-5.501485601	7.71E-08	1.43E-06
KLF9	-0.791202937	2.619856231	-8.065623299	1.47E-14	9.75E-13
HLA-DQA1	-0.790885532	3.394880039	-4.851428606	1.92E-06	2.67E-05
GLIS2	-0.788836537	2.381086913	-8.701329093	1.75E-16	1.71E-14
WNT2	-0.788709067	1.144781578	-7.680287636	1.94E-13	1.01E-11
MRGPRF	-0.788708275	1.486305863	-9.287796852	2.46E-18	3.34E-16
LPL	-0.788553008	1.086587632	-8.252939525	4.07E-15	3.06E-13
CD4	-0.786056611	2.985083894	-6.120281063	2.72E-09	6.59E-08
RAPSN	-0.785431393	0.765315434	-8.336711409	2.28E-15	1.80E-13
TGFBR2	-0.78462974	3.990312587	-6.965452714	1.86E-11	6.65E-10
AC005747.1	-0.783782613	1.640092238	-8.722774891	1.50E-16	1.48E-14
EVA1A	-0.782636927	1.975269772	-6.393295368	5.73E-10	1.58E-08
ADAMTS14	-0.782254187	1.393375917	-10.57796368	1.25E-22	4.02E-20
PLXNC1	-0.781754893	1.321671805	-9.930825259	1.93E-20	4.07E-18
VSTM4	-0.781723372	1.191113347	-9.51832726	4.42E-19	6.94E-17
COL4A2	-0.779735969	5.663872496	-6.845711629	3.88E-11	1.31E-09
KDELR3	-0.779539427	3.211376761	-7.313189785	2.10E-12	8.88E-11
AOC3	-0.779282092	1.177857077	-10.50872195	2.16E-22	6.52E-20
CLIP3	-0.778800166	1.405599484	-8.545641068	5.27E-16	4.66E-14
GRP	-0.777862453	0.895074371	-7.096232785	8.27E-12	3.19E-10
GALNT15	-0.776670376	0.952400185	-9.923639121	2.04E-20	4.25E-18
SH3BGR	-0.77488349	1.370913767	-7.160325731	5.53E-12	2.19E-10
WIPF1	-0.773371455	2.321413533	-8.388849311	1.58E-15	1.28E-13
CD1A	-0.771452339	1.645483242	-6.113460604	2.83E-09	6.80E-08
TGM2	-0.771328503	3.066776024	-4.801284943	2.42E-06	3.30E-05
SELENOP	-0.769280389	2.407525188	-4.659428283	4.65E-06	5.97E-05
ACKR1	-0.768723986	1.726384926	-5.199975867	3.56E-07	5.79E-06
LMOD1	-0.768626751	1.006487251	-9.801524967	5.19E-20	1.00E-17

NTM	-0.76820237	1.128706067	-10.7178721	4.11E-23	1.49E-20
CCN5	-0.765848905	0.634315287	-8.695444619	1.82E-16	1.77E-14
GPX3	-0.765690482	3.830070782	-4.342806346	1.89E-05	0.000210108
PECAM1	-0.764855859	3.272847756	-7.392421118	1.26E-12	5.63E-11
AC084880.1	-0.763996177	1.655257584	-6.4310156	4.60E-10	1.29E-08
SYPL2	-0.762821742	0.55804305	-8.399251674	1.47E-15	1.20E-13
MLLT11	-0.761184065	2.007878073	-6.467086935	3.73E-10	1.06E-08
TPSB2	-0.760992734	2.239457541	-5.182478482	3.88E-07	6.27E-06
SNRPN	-0.760563386	2.367712284	-6.693956031	9.72E-11	3.05E-09
KCNE4	-0.759755495	1.077951398	-11.70648946	1.35E-26	1.06E-23
RYR1	-0.759541569	1.668343666	-5.733549092	2.27E-08	4.64E-07
PAPSS2	-0.759398059	2.032521279	-7.5484742	4.61E-13	2.26E-11
ID4	-0.758931653	1.925043012	-7.066307769	9.96E-12	3.75E-10
NUAK1	-0.757734153	2.121009429	-7.338786929	1.78E-12	7.69E-11
KLHL30	-0.757272793	0.703655237	-8.272110644	3.56E-15	2.70E-13
NRP1	-0.756320578	2.622792125	-8.433007882	1.16E-15	9.70E-14
GPRC5B	-0.755695882	1.35041509	-7.921101027	3.90E-14	2.37E-12
PTPRM	-0.755248454	1.335441238	-12.05390941	7.53E-28	9.83E-25
SELE	-0.755111313	1.285440554	-6.029685347	4.52E-09	1.05E-07
JAM2	-0.755063469	0.89051436	-13.8039094	2.43E-34	1.87E-30
TSHZ3	-0.754838316	1.823139697	-9.335787662	1.73E-18	2.42E-16
KCNJ8	-0.752446246	1.466334758	-10.36763973	6.54E-22	1.79E-19
CWH43	-0.749834572	1.881301934	-4.034066792	6.86E-05	0.000665011
ADAMTS7	-0.749832343	1.166020368	-11.78242128	7.20E-27	6.36E-24
ALOX5	-0.7498076	1.769471961	-7.921809518	3.88E-14	2.37E-12
JSRP1	-0.749140275	1.167711301	-6.375920296	6.33E-10	1.73E-08
SERPINA1	-0.748251745	2.743784315	-5.295984622	2.20E-07	3.74E-06
FBXL7	-0.747236892	1.084872457	-10.3256565	9.08E-22	2.37E-19
PBXIP1	-0.746924821	4.380277382	-9.982789196	1.30E-20	2.82E-18
MFGE8	-0.743543643	3.896863578	-9.376512401	1.28E-18	1.82E-16
RHOBTB1	-0.741533208	1.43119973	-11.37134355	2.12E-25	1.26E-22
DUSP13	-0.741290607	0.702133291	-7.11327659	7.43E-12	2.89E-10
JPH2	-0.741170119	1.056711464	-7.210994979	4.02E-12	1.63E-10
ITGBL1	-0.741161933	0.694472006	-8.790364473	9.25E-17	9.48E-15
CLMP	-0.74010484	2.453799196	-5.957505395	6.74E-09	1.52E-07
SCG2	-0.739981904	0.79268418	-8.451214017	1.02E-15	8.63E-14
RHOBTB3	-0.739206898	1.614717978	-8.057056758	1.56E-14	1.03E-12
S1PR1	-0.737603354	1.890127726	-8.682813346	1.99E-16	1.92E-14
MMP14	-0.737237912	6.966853484	-8.308520725	2.77E-15	2.15E-13
PAM	-0.736956878	3.787172979	-9.777968225	6.21E-20	1.19E-17
MRC1	-0.736850318	1.791806191	-5.791687467	1.66E-08	3.50E-07
KANK2	-0.736555373	2.400905061	-10.1389188	3.89E-21	9.24E-19
BIN1	-0.735682438	3.031669546	-6.202837905	1.71E-09	4.31E-08
LTBP1	-0.734256912	4.398325407	-5.154004319	4.47E-07	7.10E-06
GJA1	-0.734178429	7.171656077	-4.948987868	1.21E-06	1.76E-05
HHATL	-0.73305404	0.530104423	-7.190693591	4.57E-12	1.84E-10
STRIT1	-0.731744752	0.539262731	-5.939557398	7.43E-09	1.67E-07
PALM	-0.73122952	1.277018477	-7.198983949	4.34E-12	1.75E-10
CXCR4	-0.731145362	3.479668628	-5.039121512	7.83E-07	1.18E-05
HLA-DPB1	-0.730599503	5.471261986	-4.359999345	1.75E-05	0.000196914
VASH1	-0.730027876	1.68140461	-10.19313262	2.55E-21	6.21E-19
RAMP1	-0.729804074	2.791749828	-4.422791969	1.34E-05	0.000154262
MME	-0.729297515	1.295712307	-5.966425265	6.41E-09	1.45E-07

TAFA5	-0.729273325	0.869856579	-9.285609033	2.50E-18	3.38E-16
ENG	-0.727576857	4.280786643	-7.562875267	4.19E-13	2.07E-11
CDH2	-0.726366234	0.734031672	-7.874105325	5.35E-14	3.14E-12
CYP1B1	-0.725924541	1.288640207	-5.410737651	1.23E-07	2.21E-06
MEGF6	-0.723759622	2.069997538	-7.217904967	3.85E-12	1.56E-10
SMTNL2	-0.722922521	0.638288767	-6.149540392	2.31E-09	5.67E-08
ARHGEF6	-0.722408526	1.344221335	-9.684617676	1.26E-19	2.26E-17
SLC2A10	-0.722222501	1.230755164	-8.574523964	4.30E-16	3.88E-14
CHRNG	-0.721964857	0.526357641	-8.473977799	8.73E-16	7.45E-14
SPRY1	-0.720860651	2.324612681	-9.069000597	1.23E-17	1.50E-15
PTGIS	-0.720048334	0.693760758	-7.412437168	1.11E-12	4.98E-11
CYP7B1	-0.71993177	1.390001987	-7.819984123	7.68E-14	4.36E-12
LAPTM5	-0.719219204	5.020125912	-5.071541664	6.69E-07	1.03E-05
DAAM2	-0.719209797	0.834281784	-12.81459134	1.22E-30	3.45E-27
FAM20A	-0.717956897	1.69843697	-7.020337312	1.33E-11	4.85E-10
CFI	-0.717602478	1.896608064	-5.923524467	8.12E-09	1.81E-07
ARHGAP31	-0.717079924	1.464928997	-9.527288271	4.13E-19	6.58E-17
ACHE	-0.716717004	0.928611128	-6.905692633	2.69E-11	9.38E-10
SORBS1	-0.716553977	0.807763046	-10.58961578	1.14E-22	3.69E-20
CACNB1	-0.716502914	1.118563446	-7.393905651	1.25E-12	5.58E-11
WIPI1	-0.716310387	2.946371669	-8.645938175	2.59E-16	2.45E-14
ITM2A	-0.715622227	1.929535923	-6.619704055	1.51E-10	4.59E-09
MYH8	-0.714196307	0.487596006	-7.978844281	2.64E-14	1.67E-12
SEMA6C	-0.714067295	1.197975693	-7.905887064	4.32E-14	2.60E-12
PHLDB1	-0.713992552	1.475812767	-10.86360846	1.28E-23	5.06E-21
TXNIP	-0.713664636	5.65793607	-4.509827171	9.11E-06	0.000109285
AOC1	-0.713533946	1.083865229	-7.777393139	1.02E-13	5.64E-12
FKBP7	-0.71334686	1.473162021	-9.769733818	6.62E-20	1.26E-17
SLIT3	-0.712809075	1.081970298	-8.168739948	7.26E-15	5.17E-13
PAMR1	-0.712386828	1.215091145	-7.026287912	1.28E-11	4.69E-10
ADAMTS4	-0.712159452	1.716720686	-7.839695981	6.74E-14	3.88E-12
EPHA3	-0.712053043	0.580554741	-12.18403745	2.53E-28	3.88E-25
ENPP2	-0.7116152	1.351716847	-8.315914419	2.63E-15	2.04E-13
PTP4A3	-0.710681726	3.057598694	-5.688563282	2.89E-08	5.79E-07
CCDC3	-0.70970486	2.93702046	-5.532882881	6.55E-08	1.23E-06
HSPB2	-0.708304097	0.914875926	-7.792193884	9.25E-14	5.16E-12
TSPAN2	-0.708202693	0.942599945	-9.482958091	5.76E-19	8.81E-17
TOX2	-0.707617724	1.800069215	-6.593072069	1.77E-10	5.32E-09
ITGB6	-0.706410142	4.20761362	-4.472692161	1.07E-05	0.000126581
RASSF2	-0.705745487	1.648986379	-7.83556592	6.93E-14	3.96E-12
TIMP1	-0.705399687	6.514318076	-5.049987159	7.43E-07	1.13E-05
SNTB1	-0.705352869	1.186279284	-11.44154831	1.19E-25	7.63E-23
PDE4B	-0.705136784	1.389897975	-7.551544141	4.51E-13	2.22E-11
GPR183	-0.704581477	2.579861279	-6.108609267	2.91E-09	6.98E-08
TCF4	-0.702856801	1.680657585	-9.937602463	1.84E-20	3.88E-18
SPTB	-0.702796737	0.643865441	-7.165555183	5.35E-12	2.13E-10
SH3PXD2B	-0.702751397	3.409987292	-8.381502227	1.67E-15	1.35E-13
ANTXR2	-0.702366851	2.059637981	-6.911402666	2.60E-11	9.09E-10
PLPP1	-0.702323209	3.086641526	-8.600463257	3.58E-16	3.28E-14
RAB3IL1	-0.7016297	1.777898558	-8.880401403	4.84E-17	5.16E-15
GYPC	-0.700194681	1.703077586	-7.938656108	3.47E-14	2.14E-12
MAP1B	-0.699521867	1.712304905	-6.570038009	2.03E-10	6.02E-09
FGD5	-0.699140648	1.212579134	-10.49479403	2.41E-22	7.12E-20

RHOJ	-0.699015217	1.31868074	-10.9303442	7.52E-24	3.10E-21
HLA-DPA1	-0.696264539	4.614651119	-3.863033291	0.000135509	0.00121801
DLC1	-0.695257346	1.185701187	-10.65266052	6.90E-23	2.37E-20
ABI3BP	-0.695256477	0.653997078	-9.026961013	1.67E-17	1.97E-15
FPR3	-0.694400685	2.610993404	-5.231839768	3.04E-07	5.00E-06
SYT11	-0.694025526	1.381738714	-10.32793374	8.92E-22	2.34E-19
CDH5	-0.691295007	1.944029015	-8.077683749	1.35E-14	9.14E-13
ARSI	-0.688629574	3.283233462	-4.866064699	1.79E-06	2.51E-05
CPA3	-0.688461369	2.07460146	-4.720046137	3.53E-06	4.63E-05
C1QTNF6	-0.688166962	2.734186835	-6.797360557	5.21E-11	1.72E-09
MYLK	-0.687543426	1.786756333	-7.520282892	5.53E-13	2.66E-11
GPX8	-0.687164502	2.725519347	-7.940418665	3.43E-14	2.12E-12
GUCY1B1	-0.686667466	1.651400671	-7.40977419	1.13E-12	5.06E-11
PRKG1	-0.686659941	0.957005361	-11.73657713	1.05E-26	8.80E-24
GPNMB	-0.684516749	6.29619135	-3.958518025	9.29E-05	0.00087054
TNFSF12	-0.683545172	2.393161171	-8.6022082	3.54E-16	3.25E-14
SHISAL1	-0.682893432	1.481412614	-6.15209647	2.28E-09	5.60E-08
CCL11	-0.682590796	1.451296711	-5.295091756	2.21E-07	3.75E-06
ANPEP	-0.681716411	2.530736726	-6.542145246	2.40E-10	7.03E-09
AMPD1	-0.680706473	0.517741841	-6.800558142	5.11E-11	1.69E-09
ITGB5	-0.679683013	4.799256195	-8.461687311	9.51E-16	8.10E-14
RARRES1	-0.67965876	1.923210659	-4.418033151	1.36E-05	0.000157158
PLEK	-0.679552059	2.432423511	-4.912824115	1.43E-06	2.05E-05
XYLT1	-0.679349636	1.23612842	-7.997215255	2.34E-14	1.49E-12
QPCT	-0.678853364	1.457876667	-7.983144596	2.57E-14	1.63E-12
CACNG6	-0.678810839	0.499402021	-7.8812279	5.10E-14	3.02E-12
OGN	-0.678394905	0.49503078	-8.610090533	3.34E-16	3.10E-14
EML1	-0.67835754	1.513611161	-8.484313264	8.12E-16	7.00E-14
SMARCA1	-0.677514823	2.600600185	-5.601160005	4.58E-08	8.86E-07
CALHM2	-0.676403764	2.192955108	-8.602184901	3.54E-16	3.25E-14
RRAD	-0.676311473	4.392275594	-2.930864268	0.003622933	0.021110505
TPSAB1	-0.676185524	2.338420341	-4.934960097	1.29E-06	1.86E-05
VGLL3	-0.675912181	0.880640838	-9.245289821	3.37E-18	4.46E-16
TCEAL7	-0.675290757	0.798821966	-10.95369925	6.24E-24	2.63E-21
SYNC	-0.674529257	0.968651379	-10.25955598	1.52E-21	3.82E-19
GFPT2	-0.671922963	2.169864034	-6.157682971	2.21E-09	5.44E-08
ISM1	-0.671547022	1.225121857	-7.849522279	6.31E-14	3.65E-12
APBB1	-0.67117055	1.2185953	-9.290870005	2.41E-18	3.27E-16
ACTG2	-0.670270877	1.134615163	-6.262117894	1.22E-09	3.15E-08
SFRP1	-0.670050814	2.436188965	-3.27277774	0.00118108	0.008115228
FITM1	-0.669630422	0.73912567	-6.39133325	5.79E-10	1.59E-08
ETS1	-0.669507253	3.664924252	-6.544863317	2.36E-10	6.93E-09
CD53	-0.669275049	3.344195255	-4.82597664	2.16E-06	2.97E-05
RASD2	-0.6691893	1.236501331	-6.063881098	3.73E-09	8.80E-08
ATP8B2	-0.669086145	1.917819796	-7.370751153	1.45E-12	6.41E-11
CLSTN2	-0.668837728	0.54373242	-10.49753431	2.35E-22	7.00E-20
TGFB1I1	-0.668804998	2.257478839	-8.245296319	4.29E-15	3.20E-13
SLCO2B1	-0.668463103	1.690532133	-6.372284813	6.47E-10	1.76E-08
LAMB1	-0.668397993	4.474007429	-6.7081883	8.92E-11	2.81E-09
LTB	-0.668346909	2.325430138	-5.689545818	2.87E-08	5.77E-07
DCLK1	-0.668272622	0.540385583	-10.65335158	6.87E-23	2.37E-20
FYN	-0.668222807	2.00794796	-9.402166388	1.05E-18	1.53E-16
SYDE1	-0.6681655	1.983670195	-8.889269625	4.54E-17	4.89E-15

PDE4DIP	-0.666528178	1.356273794	-8.420682691	1.27E-15	1.05E-13
SLC2A5	-0.666115271	1.122320392	-8.373788823	1.76E-15	1.40E-13
DKK3	-0.664879182	4.378280355	-5.280341616	2.38E-07	4.01E-06
CTSO	-0.664838709	2.85039283	-6.84938125	3.80E-11	1.29E-09
SPI1	-0.664467886	3.180746648	-5.579820576	5.13E-08	9.85E-07
ADA2	-0.664127346	2.309788092	-5.185040434	3.83E-07	6.20E-06
VWF	-0.663250516	3.369587802	-6.019480915	4.78E-09	1.11E-07
PI16	-0.662646026	0.653531275	-5.270341602	2.51E-07	4.19E-06
DMPK	-0.662522493	2.555226157	-6.307748188	9.39E-10	2.48E-08
P3H1	-0.662212199	2.875013255	-8.027365933	1.90E-14	1.24E-12
TNFRSF19	-0.661512194	1.485987211	-6.965385783	1.86E-11	6.65E-10
COL22A1	-0.660796037	1.399695433	-4.270664018	2.57E-05	0.000275946
PTPRC	-0.659760514	1.985748313	-5.194747394	3.65E-07	5.93E-06
SESN3	-0.659701748	3.300669631	-5.257446731	2.67E-07	4.46E-06
PCDH18	-0.659521106	0.992148861	-10.2031814	2.36E-21	5.79E-19
CLEC14A	-0.658859234	2.24213569	-8.048306697	1.65E-14	1.08E-12
PAPLN	-0.65876122	1.912733476	-6.635356141	1.38E-10	4.22E-09
NFATC4	-0.657997569	1.364678164	-9.651475977	1.62E-19	2.81E-17
CD163	-0.656964177	2.31225209	-4.479962043	1.04E-05	0.000122998
LPXN	-0.656445616	2.598813404	-6.278923143	1.11E-09	2.89E-08
RAI14	-0.656163727	3.206959171	-6.041519203	4.23E-09	9.88E-08
CHRD	-0.65611236	0.653063684	-13.69494473	6.26E-34	4.19E-30
TIE1	-0.655788209	1.642680773	-8.834256369	6.75E-17	7.01E-15
BARX1	-0.655302171	1.677280477	-4.466668174	1.10E-05	0.000129382
AC015922.2	-0.654653742	1.218468892	-6.654985094	1.23E-10	3.78E-09
PIK3R1	-0.654037589	2.088421423	-7.305322771	2.21E-12	9.33E-11
FBP1	-0.653901656	2.08722639	-3.973302937	8.76E-05	0.000825434
ST6GAL1	-0.653829329	1.723665306	-6.046106042	4.12E-09	9.66E-08
ZEB2	-0.65178503	0.930161755	-11.91988379	2.30E-27	2.32E-24
NTN4	-0.650278902	1.961389864	-7.002324679	1.48E-11	5.38E-10
LXN	-0.650199273	1.945583782	-6.265828444	1.19E-09	3.09E-08
SLC2A3	-0.649311317	2.326908142	-5.359889787	1.59E-07	2.79E-06
FRZB	-0.648591154	0.785352831	-8.862450883	5.51E-17	5.82E-15
IL7R	-0.648239023	2.740979403	-4.880903357	1.67E-06	2.35E-05
ARHGEF25	-0.648159808	1.446869969	-8.055881033	1.57E-14	1.03E-12
IGHV4-31	-0.647479661	2.406746223	-2.663780796	0.008117123	0.041339404
CTSS	-0.647302902	3.616492291	-4.44517348	1.21E-05	0.000140978
NAPSB	-0.647067419	1.766685929	-5.670880475	3.17E-08	6.31E-07
ITGB1	-0.646912081	5.591019116	-5.249214153	2.79E-07	4.63E-06
GJA4	-0.646903892	1.865995103	-7.334608877	1.83E-12	7.88E-11
RNASE1	-0.646562521	5.156524799	-4.939889073	1.26E-06	1.82E-05
CELF2	-0.646294968	1.268791048	-8.157371148	7.85E-15	5.52E-13
LRRC39	-0.64595164	0.648292095	-7.535845803	5.00E-13	2.43E-11
PROS1	-0.64587039	2.310628998	-5.38938148	1.37E-07	2.44E-06
HSPA12B	-0.645653093	1.144517329	-10.53107598	1.81E-22	5.69E-20
STARD13	-0.645012938	1.355483644	-10.57197828	1.31E-22	4.19E-20
SH3RF3	-0.645000268	1.378647624	-9.616221863	2.12E-19	3.57E-17
TNS2	-0.644145275	1.765170328	-9.181047366	5.41E-18	6.93E-16
MNDA	-0.644088508	1.95503678	-5.824949736	1.39E-08	2.96E-07
EVI2B	-0.643855516	2.051388753	-5.698128506	2.74E-08	5.54E-07
MEOX2	-0.643337389	0.53032832	-11.94693322	1.84E-27	1.93E-24
TMEM45A	-0.64321953	3.871338524	-3.934389365	0.00010229	0.000951105
IL32	-0.643200085	4.494740289	-4.343627469	1.88E-05	0.000209498

TRAC	-0.642018202	3.152461967	-4.37291261	1.66E-05	0.000187492
MYLK2	-0.640158298	0.551110876	-7.499646145	6.33E-13	2.99E-11
VSIG4	-0.639673998	2.015572113	-4.412646666	1.40E-05	0.00016051
HLA-DRA	-0.639290947	8.225891008	-3.520408592	0.000493277	0.003785854
TGFB2	-0.638604419	1.48091405	-6.817695176	4.60E-11	1.54E-09
FGL2	-0.638152862	2.209951376	-4.74172615	3.19E-06	4.23E-05
LBH	-0.637860546	3.647605204	-5.821746056	1.41E-08	3.00E-07
PLVAP	-0.636996553	4.893291865	-7.052799439	1.08E-11	4.04E-10
ITGA1	-0.636647251	1.416708306	-9.714823398	1.00E-19	1.83E-17
LDOC1	-0.636132241	2.355221757	-3.678149893	0.000275413	0.002271453
IGFL3	-0.634984706	1.445244965	-3.455563046	0.000623069	0.004644257
MEIS3	-0.634578601	1.18827438	-8.694317731	1.84E-16	1.78E-14
RASL12	-0.634147528	1.112491652	-9.999177138	1.14E-20	2.51E-18
CD52	-0.634017764	3.411663559	-4.303343475	2.24E-05	0.000244188
CDK14	-0.633742751	1.588487496	-6.925673437	2.38E-11	8.40E-10
SGCB	-0.633708658	2.671469814	-8.171365963	7.13E-15	5.09E-13
RUNX2	-0.633660538	1.533508956	-9.391879782	1.14E-18	1.64E-16
C1QC	-0.63349106	5.808010446	-3.663315572	0.000291184	0.002384627
CALU	-0.63317346	5.400403467	-7.336075809	1.81E-12	7.81E-11
LZTS1	-0.632991791	1.216593392	-8.391665322	1.55E-15	1.26E-13
TLR4	-0.632735036	1.121644091	-7.716377055	1.53E-13	8.13E-12
MOCS1	-0.632245164	1.424997384	-9.676238713	1.34E-19	2.37E-17
SELL	-0.631593454	1.741899859	-5.200867598	3.54E-07	5.77E-06
CCL13	-0.631406321	1.983680347	-4.44501835	1.21E-05	0.000141043
ACKR3	-0.631254165	4.717493706	-4.159086583	4.11E-05	0.000421122
LTBP3	-0.6307781	2.516648478	-6.595061802	1.75E-10	5.27E-09
MAGED1	-0.630648094	4.260719542	-5.572974524	5.31E-08	1.02E-06
PLAC9	-0.630409649	1.143531016	-7.732101526	1.38E-13	7.38E-12
PLXND1	-0.629700326	2.942730352	-7.39951974	1.21E-12	5.39E-11
KCTD12	-0.628881078	3.222124019	-6.974058863	1.77E-11	6.33E-10
SYT7	-0.627833263	2.17150929	-4.953313796	1.18E-06	1.72E-05
CFH	-0.626724182	2.972369133	-5.261684705	2.62E-07	4.37E-06
G0S2	-0.626146343	3.11485751	-3.520012163	0.000493987	0.003790303
MICAL2	-0.625745831	2.395828306	-6.344850434	7.58E-10	2.04E-08
GALNT10	-0.625219362	2.489262205	-9.041477059	1.50E-17	1.81E-15
BOC	-0.624328457	1.030857387	-9.645386603	1.70E-19	2.93E-17
AC134312.5	-0.623915643	0.826797456	-9.74306063	8.10E-20	1.51E-17
ALDH1B1	-0.623900883	2.932383948	-5.541916355	6.25E-08	1.18E-06
C3orf80	-0.623609457	0.758815573	-11.535328	5.52E-26	3.84E-23
TENM3	-0.623412207	1.623624159	-6.265583621	1.20E-09	3.09E-08
EGFL6	-0.62331866	2.091642405	-5.246738937	2.82E-07	4.68E-06
CST6	-0.623100848	3.89001795	-3.105126272	0.002071655	0.013135204
TYROBP	-0.622926421	4.8583544	-4.296766715	2.30E-05	0.000249963
ESAM	-0.622116725	2.524767632	-8.604909778	3.47E-16	3.20E-14
PCDHGC3	-0.621600735	2.61591081	-4.849117	1.94E-06	2.70E-05
SYNM	-0.620235841	1.70810157	-5.376303217	1.47E-07	2.58E-06
CRTAC1	-0.617459518	0.559379021	-7.334429758	1.83E-12	7.88E-11
SNAI1	-0.616888923	1.812798244	-7.242468482	3.29E-12	1.36E-10
RASSF4	-0.616235536	1.480664566	-7.776846019	1.02E-13	5.66E-12
EVC	-0.616176498	2.017886183	-7.779708401	1.00E-13	5.57E-12
ANOS1	-0.616134211	1.065221154	-8.057299293	1.55E-14	1.03E-12
SCARA5	-0.615952665	0.857561846	-7.077327463	9.30E-12	3.53E-10
SERPINH1	-0.615820841	5.952261942	-5.673024989	3.14E-08	6.24E-07

ENC1	-0.615602119	2.430596283	-6.610113701	1.60E-10	4.83E-09
BMF	-0.61395059	1.716684036	-8.396986933	1.50E-15	1.22E-13
HLA-DOA	-0.613911746	2.089049585	-4.267048968	2.61E-05	0.000279648
CHRDL1	-0.613455332	0.549160264	-6.519768895	2.74E-10	7.93E-09
SLC39A14	-0.612706353	3.447535992	-5.860754879	1.14E-08	2.47E-07
PDGFC	-0.612685503	2.297253543	-6.139451565	2.45E-09	5.98E-08
IGFBP3	-0.61234309	5.276687168	-3.542279259	0.000455547	0.003524152
CACNA2D1	-0.612295777	1.10286241	-6.162432115	2.15E-09	5.31E-08
PDGFB	-0.611439892	2.61071303	-6.538435123	2.45E-10	7.18E-09
CDHR1	-0.611058952	1.863590296	-4.413623397	1.39E-05	0.000159966
INHBB	-0.610040616	1.792492503	-5.254880235	2.71E-07	4.51E-06
PODNL1	-0.609920572	1.868867886	-7.128315364	6.76E-12	2.65E-10
SELP	-0.609582104	0.918416396	-6.489554947	3.27E-10	9.38E-09
TSPAN11	-0.608957913	1.325798968	-6.585947384	1.85E-10	5.53E-09
IFITM2	-0.607950481	4.764165389	-5.156136975	4.42E-07	7.04E-06
CD34	-0.607568653	1.775177582	-8.510459777	6.76E-16	5.87E-14
OLR1	-0.606332352	1.531744224	-4.406260458	1.44E-05	0.000164645
CXXC5	-0.605928242	2.953878998	-7.269426119	2.78E-12	1.16E-10
TRIM55	-0.605923551	0.432429152	-8.987212509	2.23E-17	2.55E-15
CTSH	-0.605856581	3.6170012	-5.637174626	3.79E-08	7.43E-07
SLITRK6	-0.605726868	1.986417743	-3.832909847	0.0001524	0.001352797
MMP19	-0.605687524	1.814638467	-6.634045289	1.39E-10	4.25E-09
LDLRAD4	-0.605407989	0.774740287	-11.86855522	3.52E-27	3.37E-24
KLF2	-0.604951748	2.329652894	-6.342363276	7.69E-10	2.06E-08
S100B	-0.604620808	2.257798396	-5.033082812	8.06E-07	1.21E-05
MPEG1	-0.604584231	2.244882203	-4.633737731	5.23E-06	6.62E-05
CD1C	-0.603276528	0.989655399	-8.332346133	2.35E-15	1.85E-13
DEPTOR	-0.60302154	1.51171443	-5.525563968	6.81E-08	1.28E-06
RFLNB	-0.602531724	2.813017661	-6.730087153	7.82E-11	2.49E-09
SELPLG	-0.602480277	2.855664998	-5.462534972	9.43E-08	1.73E-06
LRRN1	-0.602463487	0.648992105	-6.205091636	1.69E-09	4.26E-08
ARHGEF17	-0.602391543	1.973946978	-8.075973796	1.37E-14	9.21E-13
MYOM2	-0.601937308	0.714963722	-6.080114763	3.41E-09	8.09E-08
MIR635	-0.601289147	2.133755915	-7.191103892	4.56E-12	1.83E-10
SLAMF8	-0.599405879	2.332983399	-5.168943316	4.15E-07	6.66E-06
TWIST2	-0.59929531	2.116418937	-5.877754917	1.04E-08	2.27E-07
MN1	-0.598972908	2.481421201	-5.870050868	1.09E-08	2.36E-07
TRPV2	-0.598799702	2.038572832	-6.616127544	1.55E-10	4.68E-09
CDH13	-0.598653018	2.529915331	-5.423704045	1.15E-07	2.08E-06
DOK5	-0.597288968	0.531649004	-11.23412344	6.47E-25	3.33E-22
BNC2	-0.597145907	0.477300318	-12.34380776	6.62E-29	1.27E-25
LAMC1	-0.596431268	4.741902452	-6.4128816	5.11E-10	1.42E-08
ARMCX3	-0.596248766	2.450002518	-5.367196129	1.54E-07	2.70E-06
H6PD	-0.596064948	3.159183092	-7.594013946	3.42E-13	1.71E-11
HCK	-0.595951066	2.761637955	-5.538026471	6.38E-08	1.20E-06
CNRIP1	-0.595705457	1.014587058	-10.09064249	5.65E-21	1.30E-18
C1R	-0.595650285	5.782469241	-5.886158872	9.96E-09	2.18E-07
ABCC9	-0.595403196	0.569069677	-12.54883185	1.17E-29	2.41E-26
CSGALNACT	-0.594817001	2.935511475	-8.161098311	7.65E-15	5.40E-13
SEMA5A	-0.594808157	1.256095172	-6.365238125	6.74E-10	1.83E-08
ACVRL1	-0.59465262	1.868638415	-8.48395514	8.14E-16	7.00E-14
STAB1	-0.594186835	2.238033437	-6.177318553	1.97E-09	4.92E-08
FCGR3A	-0.594090069	3.31996856	-3.638947249	0.00031895	0.002579653

CHIT1	-0.593721533	0.846349887	-4.265176455	2.63E-05	0.000281547
EHD3	-0.593695803	2.394655609	-6.785136135	5.61E-11	1.84E-09
ABLIM2	-0.593417851	1.77947988	-5.759266427	1.98E-08	4.10E-07
PGM2L1	-0.592601101	1.336830805	-7.528428674	5.25E-13	2.54E-11
UBE2QL1	-0.592374321	0.751658263	-8.423077759	1.25E-15	1.04E-13
CYYR1	-0.591758796	1.774241312	-7.501222739	6.26E-13	2.97E-11
SGCE	-0.591685837	1.71178178	-5.141798776	4.74E-07	7.50E-06
ICAM1	-0.591551806	4.180014287	-4.264310691	2.64E-05	0.000282354
BMP1	-0.590857097	3.665081334	-7.974926089	2.72E-14	1.71E-12
NATD1	-0.590854034	1.906766037	-9.303112106	2.20E-18	3.03E-16
SORT1	-0.590290561	2.912952102	-5.928365448	7.91E-09	1.77E-07
RHOQ	-0.58987967	2.897851803	-9.205970939	4.51E-18	5.88E-16
CCDC92	-0.589210618	2.177192201	-9.340655285	1.66E-18	2.34E-16
SELENOM	-0.588487968	3.433435658	-4.589320017	6.39E-06	7.92E-05
CLEC3B	-0.588003491	1.396814678	-6.541695116	2.40E-10	7.05E-09
ANKH	-0.587164638	2.918939984	-6.689492933	9.98E-11	3.12E-09
FAM171A1	-0.58688	1.469617832	-5.002351273	9.35E-07	1.39E-05
MS4A6A	-0.586542018	2.060590851	-5.201126169	3.54E-07	5.77E-06
ENDOD1	-0.586365412	4.225228952	-6.775428704	5.95E-11	1.94E-09
MTURN	-0.586289851	1.970106057	-7.657781732	2.25E-13	1.16E-11
ZNF423	-0.586167542	0.527124981	-13.80320241	2.44E-34	1.87E-30
LHFPL2	-0.585796455	2.561464453	-7.938506065	3.47E-14	2.14E-12
JAK1	-0.58535326	4.664864295	-8.777948203	1.01E-16	1.02E-14
ST3GAL2	-0.58496622	1.636070221	-10.32857669	8.87E-22	2.34E-19
IRF8	-0.584833471	1.47459137	-5.810746659	1.50E-08	3.18E-07
CREB3L2	-0.584687309	2.963769989	-7.833008924	7.04E-14	4.03E-12
GNG11	-0.58437949	2.301159741	-7.127423844	6.80E-12	2.66E-10
AKT3	-0.584137516	1.401922288	-7.097162588	8.22E-12	3.18E-10
PALLD	-0.583039252	4.468981069	-7.043619197	1.15E-11	4.26E-10
CHI3L2	-0.582032463	1.580017713	-4.216060924	3.24E-05	0.000340357
ADGRF5	-0.581894404	1.57400303	-7.875161878	5.31E-14	3.12E-12
RECK	-0.581833183	0.898128785	-11.42578069	1.36E-25	8.34E-23
ADAMTS1	-0.581260206	2.469749147	-4.663636061	4.57E-06	5.86E-05
NOX4	-0.581259311	0.828894424	-10.7434301	3.35E-23	1.24E-20
CCR7	-0.581234354	1.420485931	-5.350292301	1.67E-07	2.92E-06
EVI2A	-0.581094154	1.542040044	-6.49593371	3.15E-10	9.05E-09
TMEM38A	-0.580908797	1.498855676	-4.611324688	5.79E-06	7.23E-05
GPSM3	-0.580618274	3.024271483	-5.732287532	2.29E-08	4.67E-07
LGALS1	-0.580229078	8.175953921	-4.195904777	3.52E-05	0.000366678
MAPRE3	-0.580142392	2.342555416	-5.712947648	2.54E-08	5.15E-07
SNHG25	0.5812132	1.425524006	5.66753892	3.23E-08	6.41E-07
PPIH	0.582399869	4.139530309	7.908435277	4.25E-14	2.56E-12
ATP5MC3	0.582752725	4.743175611	7.8894628	4.83E-14	2.87E-12
UQCRH	0.583498548	6.226047959	8.527554644	5.99E-16	5.26E-14
STAP2	0.583999847	4.460107295	6.847913147	3.83E-11	1.30E-09
KIF18B	0.586822945	2.386642944	7.629836052	2.70E-13	1.37E-11
CENPU	0.587096587	2.949470973	6.493080893	3.20E-10	9.20E-09
POLE2	0.587198322	1.778239738	9.615332944	2.13E-19	3.59E-17
C1QBP	0.587291036	5.344760249	8.787286588	9.45E-17	9.66E-15
EXO1	0.58755266	2.046394058	8.445194084	1.07E-15	8.96E-14
UPP1	0.587802946	4.536063882	4.43136643	1.29E-05	0.000148979
TACC3	0.588410963	3.450488307	7.828029891	7.28E-14	4.15E-12
EIF4EBP1	0.588526551	5.968803654	5.388487961	1.38E-07	2.45E-06

CSTB	0.590252746	8.199766351	3.847417981	0.00014403	0.001286182
AKR1C3	0.591232502	3.02535788	2.598014949	0.009809094	0.048186632
NCAPG	0.592924471	2.64301564	8.503896807	7.07E-16	6.12E-14
VRK1	0.593567642	3.008192739	8.986550365	2.24E-17	2.55E-15
CENPA	0.594625515	2.628397263	8.074342695	1.38E-14	9.27E-13
RPL39L	0.59484899	3.367790337	3.231503434	0.001359291	0.009162245
FOXM1	0.595035444	3.814007766	5.794637947	1.64E-08	3.44E-07
DCTPP1	0.597007626	4.562988506	7.901255557	4.46E-14	2.67E-12
RPL36A	0.597956944	3.919530307	6.177285358	1.97E-09	4.92E-08
RPL36	0.598337032	7.271492233	6.202671521	1.71E-09	4.31E-08
ARHGEF16	0.598942363	2.529971048	5.928406476	7.90E-09	1.77E-07
CDCA3	0.599763134	2.032099235	8.462552231	9.46E-16	8.06E-14
MIS18A	0.600265706	3.011059761	9.200113028	4.71E-18	6.10E-16
CENPM	0.602674843	2.798353889	6.543258763	2.38E-10	6.99E-09
BUB1B	0.605141325	2.54761528	7.725738865	1.44E-13	7.66E-12
POC1A	0.60582152	2.567657942	8.629001598	2.92E-16	2.72E-14
SPC25	0.606331831	2.332832727	8.747791541	1.25E-16	1.25E-14
LMNB2	0.607704991	5.070440249	8.498109894	7.37E-16	6.36E-14
CCDC58	0.610990747	3.093922059	8.697997842	1.79E-16	1.74E-14
NME1	0.613386769	4.2219881	8.333948429	2.32E-15	1.83E-13
NOP16	0.613503958	3.527198303	9.539484347	3.77E-19	6.04E-17
CENPH	0.613820425	2.594229516	7.643182343	2.48E-13	1.27E-11
LMNB1	0.613931681	3.844491636	6.485988629	3.34E-10	9.57E-09
ANXA1	0.614048744	8.076870934	4.217100292	3.22E-05	0.000339008
DLGAP5	0.615273427	3.169975374	7.816086789	7.89E-14	4.46E-12
SCD	0.620041554	6.035614585	4.443446829	1.22E-05	0.000141832
KIF11	0.620336867	3.312619592	8.134728393	9.16E-15	6.39E-13
IL36G	0.620816718	4.149385257	2.596383669	0.009854807	0.048349076
COA6	0.62210119	4.116493431	7.710113475	1.59E-13	8.44E-12
MRPL11	0.622206359	4.148433666	7.428443532	1.00E-12	4.53E-11
MRPL21	0.623008345	3.829470557	5.819647556	1.43E-08	3.04E-07
RPL35	0.623696668	8.379754627	6.901455625	2.76E-11	9.59E-10
ZMYND19	0.624209765	3.658467996	8.959476688	2.73E-17	3.03E-15
SNRPG	0.627600287	4.588739638	9.023728384	1.71E-17	2.01E-15
HSPD1	0.628858879	6.137192935	8.604962101	3.47E-16	3.20E-14
AURKA	0.629612578	3.457639302	7.707573213	1.62E-13	8.57E-12
PAICS	0.629791058	4.262859704	10.0446724	8.05E-21	1.80E-18
SLIRP	0.62992306	3.774419093	8.114894391	1.05E-14	7.21E-13
ATP5MC1	0.631484505	4.582163606	8.452984407	1.01E-15	8.55E-14
CRB3	0.633800425	3.232909979	6.321910873	8.65E-10	2.30E-08
TOMM40	0.634176807	4.669486814	10.59726892	1.07E-22	3.54E-20
SNRPGP2	0.634654638	3.437335038	9.080952654	1.13E-17	1.39E-15
ELF3	0.636618585	3.279766785	3.629942311	0.000329827	0.002657984
FKBP4	0.637641554	5.264523779	8.410136895	1.36E-15	1.13E-13
TRIP13	0.639971213	3.052595685	7.078547628	9.23E-12	3.50E-10
TXN	0.639978311	8.146705775	6.33028682	8.24E-10	2.20E-08
TKT	0.640557031	5.106804815	7.74089294	1.30E-13	7.02E-12
GAS5	0.641089074	4.499654535	5.487897583	8.27E-08	1.53E-06
NUF2	0.64237361	2.260796715	7.705738632	1.64E-13	8.66E-12
KPNA2	0.643063441	5.808945329	9.719634862	9.68E-20	1.77E-17
FXYD3	0.643658516	6.745985258	4.934687134	1.29E-06	1.87E-05
SNRPE	0.643853269	4.771748455	8.411568744	1.35E-15	1.12E-13
FAM83D	0.643932921	3.983064363	6.584211831	1.87E-10	5.58E-09

NUDT1	0.644546925	3.766186645	7.35089022	1.65E-12	7.17E-11
TUBB4B	0.645269688	8.068785214	9.163390062	6.16E-18	7.80E-16
MRPL12	0.647211024	4.833998076	7.205138459	4.17E-12	1.69E-10
GINS1	0.648404124	2.720332516	8.753708713	1.20E-16	1.20E-14
LINC01133	0.649029587	2.130617447	3.766927387	0.000196609	0.001694366
ANP32B	0.649667782	6.671496461	9.967868371	1.45E-20	3.15E-18
AC105460.1	0.649729801	2.108758732	2.597640811	0.009819561	0.048211506
KIF22	0.65106634	3.943496507	10.08347634	5.97E-21	1.37E-18
NMRAL2P	0.651859964	1.437713293	3.757701603	0.000203679	0.001746299
SLC25A10	0.653750511	2.735390015	7.651627109	2.34E-13	1.21E-11
H2AFX	0.653847694	5.110740939	7.225360609	3.67E-12	1.50E-10
E2F1	0.653872179	3.011899695	6.517049956	2.78E-10	8.05E-09
FANCI	0.653978541	2.85631218	8.881852047	4.79E-17	5.13E-15
SPAG5	0.654952216	2.79054521	8.590339783	3.84E-16	3.51E-14
SNHG1	0.655364088	3.258280977	6.290979209	1.03E-09	2.71E-08
ODC1	0.65636114	5.986271678	3.552775723	0.000438416	0.003405936
FBL	0.656362133	5.969235282	7.762819457	1.12E-13	6.17E-12
SNRPD1	0.658988142	3.60878967	9.888227692	2.68E-20	5.43E-18
OIP5	0.660041742	2.106972532	8.649620893	2.53E-16	2.40E-14
C17orf53	0.660126005	1.885356342	9.522305028	4.29E-19	6.75E-17
NUDT8	0.661522594	3.104746494	6.328658265	8.32E-10	2.22E-08
CDCA2	0.661569184	2.070624389	9.061077725	1.30E-17	1.58E-15
AC010503.4	0.662486537	3.073804716	5.954417324	6.85E-09	1.55E-07
SURF2	0.662643048	4.494908063	8.199412149	5.88E-15	4.29E-13
DTL	0.662840715	2.421054493	8.204873685	5.66E-15	4.16E-13
GGCT	0.664242402	4.836869846	9.827583975	4.26E-20	8.29E-18
SKA3	0.6651	2.306887677	10.08164666	6.05E-21	1.38E-18
SNHG15	0.66520658	2.901246173	7.354061262	1.62E-12	7.05E-11
RMI2	0.665553552	2.786033845	6.990728303	1.59E-11	5.75E-10
C16orf74	0.665583228	3.295916724	7.030226561	1.25E-11	4.60E-10
TYMS	0.665900148	3.553540302	6.925835997	2.38E-11	8.40E-10
F12	0.667112745	2.681827595	7.447859026	8.85E-13	4.05E-11
ZIC2	0.667123449	1.344307538	6.621612449	1.50E-10	4.55E-09
CEP55	0.668985434	3.814558984	7.648205596	2.40E-13	1.23E-11
CDKN3	0.672041157	3.210555643	8.181880127	6.63E-15	4.75E-13
SNHG19	0.672169669	4.019365041	4.53164414	8.27E-06	0.000100047
SERPINB1	0.672611755	6.284715783	5.108267376	5.59E-07	8.73E-06
RECQL4	0.679198918	2.979833396	7.1702192	5.20E-12	2.07E-10
MAD2L1	0.679801667	2.502732334	8.914037198	3.79E-17	4.14E-15
ADH7	0.680557944	2.349435171	2.681236678	0.007714517	0.039689277
GSTA1	0.680799116	1.012663964	3.157063782	0.001745077	0.011353713
TK1	0.681473645	5.774547594	7.169148184	5.23E-12	2.08E-10
MND1	0.682224386	1.851752574	9.012947048	1.85E-17	2.16E-15
WDR34	0.683113258	4.543600728	6.90442429	2.71E-11	9.45E-10
FEN1	0.683484539	4.448045572	8.803409213	8.42E-17	8.68E-15
PCNA	0.683940763	6.410526678	8.570921008	4.41E-16	3.97E-14
NDC80	0.684023054	2.703547527	8.184641687	6.51E-15	4.68E-13
NEK2	0.685108075	2.622839042	8.403103168	1.43E-15	1.18E-13
RPL22L1	0.687975353	4.002876336	6.034420569	4.40E-09	1.03E-07
SLPI	0.68887482	8.938989768	3.212251477	0.001450667	0.009684264
ORC1	0.689084241	2.341206715	9.128795626	7.95E-18	9.94E-16
KIF2C	0.689719562	3.593821381	8.647956675	2.56E-16	2.42E-14
SNRPF	0.690076344	4.01453942	9.937785426	1.83E-20	3.88E-18

RRM2	0.691592715	4.173454781	8.086417808	1.27E-14	8.63E-13
MAL2	0.691661909	6.18232288	5.074938321	6.58E-07	1.01E-05
TROAP	0.691727664	2.504277033	8.980055654	2.35E-17	2.65E-15
UBE2S	0.692507704	3.789477862	7.317955395	2.04E-12	8.65E-11
PTTG1	0.693578773	4.702466873	7.637067234	2.58E-13	1.32E-11
CDC25A	0.696141352	1.78180736	10.9510313	6.37E-24	2.66E-21
RAD51	0.69637006	2.497129055	10.3731701	6.26E-22	1.74E-19
CDC45	0.699391991	3.009026845	7.851700094	6.22E-14	3.61E-12
PLK1	0.699636419	3.636747275	8.882415078	4.77E-17	5.12E-15
TPX2	0.701387113	4.851099046	7.849605943	6.31E-14	3.65E-12
RFC4	0.701623478	3.226748928	7.477007262	7.33E-13	3.41E-11
NPM3	0.70350504	5.08359689	7.565189438	4.13E-13	2.04E-11
CHAF1A	0.705781418	3.15087221	9.585231238	2.67E-19	4.39E-17
HSPE1	0.706867433	5.213643494	8.517278477	6.44E-16	5.61E-14
S100A2	0.707944671	9.923603231	5.028528809	8.24E-07	1.24E-05
MCM2	0.708127871	4.313967508	6.916626405	2.52E-11	8.86E-10
CCNA2	0.708223308	3.822452137	8.554280396	4.96E-16	4.41E-14
MELK	0.712321973	3.378603702	7.959139692	3.02E-14	1.88E-12
HMGA1	0.71622939	7.21586477	8.141680033	8.74E-15	6.13E-13
RNASEH2A	0.719019353	4.194602572	8.669234192	2.20E-16	2.11E-14
NUSAP1	0.724251646	4.053476713	7.725725158	1.44E-13	7.66E-12
KRTCAP3	0.72455622	3.086390764	3.812001139	0.000165275	0.001454059
ZWINT	0.727302559	4.035494745	9.074834918	1.18E-17	1.45E-15
RPE65	0.727597223	0.97001266	5.750655919	2.07E-08	4.28E-07
TMEM97	0.7279449	2.910692651	7.813568197	8.02E-14	4.52E-12
AKR1C1	0.728662183	2.520462053	3.07221802	0.002306809	0.014401011
KIFC1	0.733280133	3.711250045	9.295031695	2.33E-18	3.18E-16
MCM7	0.733728864	5.242705026	8.075051981	1.38E-14	9.26E-13
PSAT1	0.734848835	4.18991131	6.6372755	1.36E-10	4.18E-09
MYBL2	0.736856293	4.822692222	7.933206954	3.60E-14	2.21E-12
H2AFZ	0.742733263	6.379367547	10.10111443	5.21E-21	1.22E-18
PKMYT1	0.742738176	2.971907631	9.30062347	2.24E-18	3.06E-16
HMGB2	0.742821976	5.027542168	7.693878082	1.77E-13	9.32E-12
GMNN	0.744257931	3.174969502	10.36611608	6.61E-22	1.81E-19
TGM1	0.750609936	5.403878134	3.015018964	0.002774772	0.016845011
CDCA5	0.752754269	3.633795235	9.302036683	2.22E-18	3.04E-16
ASF1B	0.756624945	3.688526777	8.460342248	9.60E-16	8.15E-14
VPS9D1-AS1	0.758959846	2.454168874	7.162783031	5.45E-12	2.16E-10
CDC6	0.76495289	2.754147909	9.895017233	2.54E-20	5.22E-18
CSTA	0.769571855	7.92005225	3.925654286	0.000105896	0.00097964
CXCL17	0.770319267	3.202322328	3.310949535	0.001035812	0.007237027
S100A14	0.774734356	8.535483097	5.531133396	6.61E-08	1.24E-06
SNORD104	0.779104478	2.893777788	4.595931363	6.20E-06	7.70E-05
CDK1	0.782692105	3.642360721	10.08146164	6.06E-21	1.38E-18
C19orf48	0.78272427	4.24790401	9.163558906	6.16E-18	7.80E-16
SPC24	0.784868066	2.406770052	9.000557133	2.03E-17	2.33E-15
CCNB2	0.788037738	3.738125544	10.21246492	2.19E-21	5.41E-19
PCLAF	0.790744973	2.905124315	10.23596244	1.83E-21	4.55E-19
CKS1B	0.794759963	3.917786329	9.524090046	4.23E-19	6.69E-17
BIRC5	0.796111534	4.159141522	9.265173077	2.91E-18	3.91E-16
SAPCD2	0.801703005	2.633206165	7.0010304	1.49E-11	5.42E-10
CLDN7	0.808826156	4.123011571	4.721896159	3.50E-06	4.59E-05
GINS2	0.815405184	2.705263787	9.750665911	7.65E-20	1.43E-17

UBE2C	0.823725467	5.233095892	8.945231192	3.03E-17	3.34E-15
CBLC	0.827093143	4.744244536	7.76113243	1.14E-13	6.23E-12
CENPW	0.83426716	4.677031715	8.40132425	1.45E-15	1.19E-13
UBE2T	0.839720376	3.84796213	9.054971336	1.36E-17	1.65E-15
SERPINB2	0.857723607	3.845454388	3.801791983	0.000171928	0.00150433
CDCA7	0.859502462	2.623460476	8.430551285	1.18E-15	9.85E-14
CDT1	0.860477476	3.2451728	10.37385291	6.23E-22	1.74E-19
S100P	0.868700118	4.203663298	3.624549959	0.000336506	0.002704479
AURKB	0.870046617	3.786244838	9.257976575	3.07E-18	4.10E-16
NMU	0.871496103	2.709591007	5.741407628	2.18E-08	4.47E-07
PBK	0.872040991	2.816216896	9.557401433	3.30E-19	5.36E-17
KRT18	0.877413502	5.364261171	5.949332612	7.05E-09	1.59E-07
AL138789.1	0.880278832	1.256049748	5.93617795	7.57E-09	1.70E-07
CCNB1	0.880709206	4.880925141	10.88058962	1.12E-23	4.48E-21
CDC20	0.889420294	5.415713296	10.17024594	3.05E-21	7.35E-19
SPRR2F	0.890456374	3.782170865	2.78815581	0.005617318	0.030599692
SPRR2A	0.902381649	8.051853906	2.736677987	0.006552273	0.034767294
EPCAM	0.917013712	3.481726258	5.136686567	4.86E-07	7.67E-06
CKS2	0.919790021	5.776434797	9.609538907	2.23E-19	3.73E-17
LGALS7	0.96795531	3.884464481	2.739461547	0.006498326	0.034551444
KRT24	0.976229487	1.340832748	4.241537932	2.91E-05	0.000308663
KRT8	1.018572826	4.286153805	3.932219396	0.000103175	0.000957774
AC006329.1	1.109263029	2.484680361	10.50322303	2.25E-22	6.73E-20
ALDH3A1	1.176395333	3.611693377	4.449326135	1.19E-05	0.000138767
FGFBP1	1.290538154	7.417448232	6.746294923	7.09E-11	2.28E-09

B
21.07080618
18.22808294
22.7575138
20.93459787
18.47007606
22.34848915
22.2625913
22.83734372
19.40036598
20.22100304
44.79207826
41.42026775
20.21054554
44.90041241
39.97859899
18.27776225
16.20061757
47.73814537
35.44022755
15.69774299
46.08953055
19.87578938
24.75180541
17.56280702
15.60589326
23.32479508
17.19776312
42.13157776
20.33476831
20.77763258
28.5815706
45.30287818
20.40051126
23.71388382
22.89857939
35.56673788
50.07427141
20.68163655
57.11498905
53.56360395
19.85004794
19.87135316
41.97579948
45.93643357
46.06372329
40.28359851
26.90828681
47.78970924
53.63879479
45.50103623
18.18065024

32.12922161
16.63703227
26.29473258
21.02071337
28.99565724
40.86014026
50.02088966
23.50106745
32.6862893
13.64738767
23.537255
10.90064905
39.68462141
18.30771109
29.77272222
27.48947364
56.50511627
18.31472878
41.10499592
35.5420675
36.7421263
42.54554568
54.33880608
46.04758402
26.69352479
44.97370472
19.2369087
18.49346382
37.93876517
38.02628664
34.60369242
69.44311992
29.09197617
28.6661031
58.18272202
22.16117117
52.64611375
22.44814866
30.37837226
21.99314457
46.64308402
25.54447722
35.80608393
33.42421115
29.09411742
35.3218877
33.04496582
47.61752144
20.92194594
39.37697697
3.399455341
29.78517754
11.12064347

32.91029421
45.18525968
18.63318062
19.73641691
13.09530432
43.33026638
19.75772502
38.10579801
17.94438442
42.10917798
41.2433638
12.33917141
41.17639038
32.2099627
23.46934343
5.398244575
28.56029494
33.06589204
27.34565133
35.26363388
51.6666317
27.27374707
44.71734106
24.23308355
34.90911635
1.567083081
51.81184729
26.78714069
21.94341199
62.77080228
48.58321351
22.2914608
32.99061139
17.13163148
44.64166736
5.400767081
22.31382701
35.20061145
28.97379062
44.37298477
40.496801
17.9877308
41.42743602
42.93429081
59.50396546
19.06059474
22.37654075
15.45845356
21.67263671
76.32110308
29.9150714
50.0634687
23.07326294

18.04879996
31.9780996
46.20816675
31.68107601
39.9430778
57.32815874
33.09559683
25.20361026
17.68797107
21.21743122
34.03142834
24.75586111
32.21263282
39.90286958
34.9364346
68.66454027
24.5441358
21.03427532
45.07266225
20.7790083
21.08868199
45.40661272
27.9354963
23.43087843
29.7720092
48.89359036
22.64233494
28.61909249
31.74490636
33.98722711
10.96175986
36.79156569
52.79159853
23.59394067
19.41784747
35.31166459
23.70642098
10.76830876
14.14471727
22.59624325
28.53547654
44.77063621
49.55870503
56.94284202
21.56372774
54.53238802
21.79997097
21.38466676
26.54594537
30.24474453
38.57347523
28.49595461
17.77596208

50.86205064
30.86292932
28.38771098
3.717724671
23.68805073
18.96402016
32.87129114
37.90875978
21.96737681
17.25198901
38.36519912
17.13533554
23.39326682
15.83935265
35.52380402
58.89548294
47.11564326
76.51594604
43.21087237
27.01388364
26.91741772
18.61689604
29.62708461
25.45153307
28.7055446
47.23730468
38.50406813
17.3816323
15.85024137
46.47472683
32.85620058
18.20293861
22.80600254
28.1232119
50.06764877
28.02992335
49.9953446
13.81621281
31.87610962
37.60710919
24.06614016
5.878060009
49.06783399
23.22026377
3.81946595
34.81648368
8.970622937
57.28523207
2.076961933
14.17389844
44.68114127
23.43227436
23.212108

17.84599048
39.57651037
9.519676721
8.08261075
3.725085771
6.852050874
20.62102616
3.32620347
22.76946968
7.030282677
20.0588213
37.40049099
19.18310892
39.63466377
36.25739439
15.61756257
31.30965737
12.65765701
28.36119571
19.98042457
13.89673032
46.50040468
33.81923646
15.11701282
12.94745536
28.97939453
53.55722639
11.83179997
17.25748016
18.37825048
11.44179806
22.21511694
10.60411588
19.26146876
3.955213775
27.16064384
16.27586567
37.14355757
24.15239166
10.08847878
18.66673636
30.3683498
31.95985871
51.8772704
51.71494008
11.8406935
28.30664818
23.7952693
34.07927331
41.60128794
37.77155103
6.749524662
22.21868144

17.73005182
42.71986477
29.80429229
18.44491122
13.55329965
22.0274144
11.85758033
10.5110241
29.22090309
14.43505862
21.96632728
24.09474429
49.20715962
60.81708991
13.90946546
27.71814209
14.59628018
21.88175141
46.30334636
49.49674662
17.18095377
19.81827378
3.612605287
32.87673285
4.524264417
45.54028242
52.199149
15.16209
31.0913506
49.27833309
16.48576749
15.09116308
32.79347082
27.30631059
13.58539488
36.0742847
24.19884213
29.72695837
30.73395949
40.49581325
62.13705796
29.05620202
18.8385621
31.66766261
14.07795955
25.40765053
9.098356593
19.52354317
32.88529579
52.62966549
52.98840628
20.59610028
43.99186054

13.9002035
21.67052488
34.54031969
16.04671174
22.21748782
11.14000245
27.63596708
-4.072131586
16.13115006
36.4649006
1.692695596
15.61717862
22.07571848
34.76011626
52.19584456
44.16812913
28.12343768
27.04634925
18.14285293
52.01688676
0.965888873
30.09530549
18.49527101
22.74387544
11.2589028
31.30817432
13.34617165
26.41501929
46.29918252
10.28655726
-2.618478361
9.778091417
20.53083015
45.42121953
18.94751323
54.3671535
28.66498097
72.53573977
22.32097886
3.219793124
12.51624357
25.33061122
-1.4597317
34.70992314
29.71733623
26.76695951
18.08316782
7.573603669
21.46527344
4.304945609
18.10923559
16.39808326
20.10334093

43.47425391
13.7684358
6.418378245
46.55241947
7.800209376
15.91089015
20.72031946
10.31896607
20.10813492
14.33700216
17.27890009
31.52995115
29.92130646
27.30580383
13.77896942
6.590599269
15.7907076
31.42994179
28.89611583
15.29900981
13.23022407
20.67080566
9.512737387
23.81096381
20.92474535
6.792556391
21.95808247
3.68525254
26.33108789
19.4143536
30.54487202
23.22443586
10.04761914
23.79688773
14.92783639
26.48224782
11.57108057
40.34195389
35.34369285
32.24479828
14.20748686
17.07131826
39.79967076
25.2406622
15.725078
35.28910293
16.1197346
24.15504606
10.01022908
3.459432661
2.831623459
5.310326836
34.36467361

41.44287635
26.28965151
1.49004817
17.57018015
11.78562496
24.22667545
11.99170041
5.226463972
13.30794838
49.40477541
7.980324569
18.56380838
15.54169704
17.23206674
23.35511112
24.45950634
20.9942125
52.27100454
9.553666636
67.13042797
30.89680133
38.70014547
0.262944154
50.0284248
20.99890923
11.47258329
5.774720049
38.37436922
35.73905945
31.19625906
46.67179638
15.82977483
16.43355018
26.96014445
9.164321648
24.58532837
21.90062616
26.20076506
23.60383128
34.18705017
8.284161867
36.9334298
10.50280506
5.090501706
4.130395717
16.30762352
9.068090644
16.35901652
4.548405421
1.56075466
37.35038908
1.821094478
9.21223337

30.52885297
18.65623565
20.68330023
6.339108635
16.4764702
10.20839365
33.48544279
25.44202721
24.74289032
28.95373955
17.69680954
20.32679697
4.700333489
58.65175446
15.26109264
8.982324359
32.31134996
14.56717002
40.4333789
17.57956357
25.94173172
12.87335541
21.37792752
20.89342423
42.59687603
2.187383052
20.04741912
34.12501514
22.65284763
15.29733967
20.45644869
53.35271718
23.65442429
7.746936321
6.950895103
20.14438767
31.98246028
12.71837575
2.030329539
20.42926552
4.599231763
47.24156304
18.58350122
9.983653886
35.39519541
16.15204876
24.10448913
14.60153217
25.62323245
27.60024608
21.11067175
12.58471693
39.69079981

43.12771667
-0.381662531
40.92887756
28.65057235
5.463659146
38.39202295
22.03904111
3.751547424
3.097894319
13.9192453
18.38323022
21.122374
17.67994853
49.65170246
-0.024913046
25.63543444
10.22246754
5.770368381
12.42334226
13.93826038
24.65778703
1.801248833
3.964498799
21.50039677
21.40657851
20.7303413
25.69057308
24.81451695
7.297784018
25.63527151
-3.439210232
4.065920964
30.23402823
43.31384162
37.86279243
10.25324316
20.52116226
30.56737707
10.83260403
-2.408641959
11.55994716
12.43904472
3.570377277
9.739424482
17.4333609
39.71221432
23.17239264
11.45200041
13.39166712
7.752017744
40.93431671
31.38527873
27.66350686

24.37442531
24.05144197
5.698568011
14.2294252
7.18899072
5.238728178
9.498396133
5.649985852
11.08813317
21.70180142
14.92743116
1.196072665
5.285242385
5.587454124
37.42779895
21.84198074
12.96466091
33.23723822
2.06098561
10.92654483
9.617855905
66.18806544
27.27169111
2.00496051
13.07939613
17.02199437
0.0310329
9.642762778
51.16145671
15.15150915
10.85332897
6.087777175
27.47231426
3.818935562
21.89274366
-4.169206833
1.914685219
7.655607947
5.547598853
17.20580151
4.088557735
22.5759641
18.4828594
6.233308129
39.97455324
40.29500989
32.97372617
29.76580597
8.459110803
7.796435634
51.3850086
-0.115809369
1.493419406

1.614023762
18.25134212
1.778808449
-1.596011295
14.04028299
3.193853257
8.442225204
15.45910306
33.71206382
-1.049796263
-1.814130603
26.28171834
35.8639715
1.328696127
14.68750345
22.67061649
31.30945138
-1.102102519
17.21502214
24.17443116
19.64899672
33.42264715
5.314607187
1.914035091
0.750070185
12.72993819
7.154160337
19.75146884
17.6150611
14.97996531
4.150310318
5.60799133
-1.597356162
11.29696977
28.75516006
33.19168444
33.92426308
6.996589356
48.00494049
10.85196123
5.535626201
-2.927329336
1.301933167
25.65432903
3.674797768
6.168689366
17.20467573
16.62928097
20.04383701
20.0625795
21.90225231
15.60916018
7.666671581

12.81749152
24.21107589
1.181457047
12.29426229
8.648338804
10.15288908
-1.521599005
10.27942315
12.40191671
1.782875765
5.575023654
15.92230669
12.1205079
12.67699558
5.100663037
24.99596322
1.752234569
16.79741454
28.364697
7.482167768
-0.492563973
12.95700786
50.7377847
11.28294059
4.520198505
2.719681052
23.76696575
6.91391878
13.5207446
6.597162203
10.51529851
22.02755778
9.827900765
16.31016602
5.16175543
8.738510242
8.697620433
12.85251501
6.403515287
45.56246605
54.68660429
11.68236112
6.123769256
18.85650538
6.976905516
36.56309738
8.783164084
56.40724164
22.60116197
11.41213035
24.81203416
10.36158866
-1.187603903

1.173890833
13.846612
8.114419082
18.43535917
24.39095231
18.26140908
5.032415214
1.170393565
21.3518314
30.65706982
9.008201028
29.94723296
30.93255488
2.527415645
12.42074232
13.28172263
4.377098248
5.315848455
13.78900348
19.26841007
67.12430755
21.10967564
26.87218503
38.39700761
8.384307974
20.41244079
15.91681732
15.73078489
15.40303823
0.976490721
20.69027643
47.11346801
2.850009674
41.64472841
6.040560822
12.15713937
2.6224619
7.973759139
0.896073121
7.873257486
7.638375544
20.91029634
20.78476447
25.11478504
14.22064729
19.08760298
12.14075302
32.96708868
26.93833299
24.54370573
1.856900161
20.37969004
6.22888915

-0.439250112
-4.338740387
24.95038572
28.35994115
22.01660548
-2.538710625
8.299647668
20.86276767
10.36140526
10.50188286
9.008420407
24.66377345
29.90456556
12.42977452
19.70998788
25.8229884
26.65886597
24.91021607
26.30762828
23.77794759
32.40198393
19.1738958
12.10003956
0.980646869
20.30118909
1.907450175
22.42304771
-4.342893947
19.60821852
17.79824618
8.431168734
14.54168592
28.16566401
28.62729492
25.654695
19.59168696
36.21130597
22.28932957
24.59757268
11.16773796
40.49345344
29.04008347
-1.219066709
24.30169041
15.61663481
11.21488235
19.80882237
6.724262103
19.57975017
33.74819772
4.064667923
24.31156254
12.66692065

17.30821387
29.63744601
16.39719674
26.70068676
-0.732712257
35.62542356
-4.339693237
36.50820286
-0.765986322
19.22855077
16.52281263
12.2786009
27.61059152
25.55247176
10.9940589
-1.485734742
19.95206104
35.02040372
25.96757062
32.27433608
11.20571215
28.89654025
9.147747912
22.86062308
22.89767321
34.56143227
36.49419028
17.32817904
15.08106962
15.32134363
14.68848351
17.92149452
12.88447915
19.20640154
22.74179772
2.280185766
4.873439593
16.18088334
27.8403861
-4.12352743
-2.769380291
16.17426075
28.5496968
14.55954018
27.05263785
25.41688497
22.7605035
24.25321074
9.579339438
-2.598850897
29.38638024
25.95589319
35.39658607

22.09772047
4.716299
28.31330404
17.10121566
19.13434453
43.29257067
38.74310832
20.53551224
27.61460713
20.52171348
18.1069508
18.67109964
32.7425214
25.04334016
4.498945734
14.63298627
25.30084897
21.24677698
22.46996548
26.10529749
19.70989854
-0.569075077
28.99587941
8.069468132
20.284645
-3.026131029
30.59786042
22.02136791
12.97586863
21.0745039
36.64335135
30.63883114
19.50262605
38.68831096
-3.195498664
30.64918793
24.64847813
16.13491678
35.07188276
-0.148591383
-2.286976672
6.942052749
2.555930622
36.49277335
29.63867274
28.46058916
37.49934818
37.68059398
32.28759336
30.37932742
15.1436431
3.10606787
33.98147291

28.06358107
19.94103042
24.24095373
28.85247525
-0.606294529
24.44254209
38.74841355
-1.237872952
30.32671735
8.021248534
32.53525002
9.120473156
9.049997225
42.73180903
37.17422869
-3.837522032
-3.976555668
5.008120755
32.92383331
-3.969100902
1.078630291
-0.123959256
39.75667877
1.932095924
13.61648103
